# Safety and Immunogenicity of a rAd35-EnvA Prototype HIV-1 Vaccine in Combination with rAd5-EnvA in Healthy Adults (VRC 012)

**DOI:** 10.1371/journal.pone.0166393

**Published:** 2016-11-15

**Authors:** Michelle C. Crank, Eleanor M. P. Wilson, Laura Novik, Mary E. Enama, Cynthia S. Hendel, Wenjuan Gu, Martha C. Nason, Robert T. Bailer, Gary J. Nabel, Adrian B. McDermott, John R. Mascola, Richard A. Koup, Julie E. Ledgerwood, Barney S. Graham, VRC012 Study Team

**Affiliations:** 1 Vaccine Research Center, National Institute of Allergy and Infectious Diseases, National Institutes of Health, Bethesda, Maryland, United States of America; 2 Biostatistics Research Branch, Division of Clinical Research, National Institute of Allergy and Infectious Diseases, National Institutes of Health, Bethesda, Maryland, United States of America; 3 Clinical Research Directorate/Clinical Monitoring Research Program, Leidos Biomedical Research, Inc., NCI Campus at Frederick, Frederick, Maryland, 21702, United States of America; Public Health England, UNITED KINGDOM

## Abstract

**Background:**

VRC 012 was a Phase I study of a prototype recombinant adenoviral-vector serotype-35 (rAd35) HIV vaccine, the precursor to two recently published clinical trials, HVTN 077 and 083. On the basis of prior evaluation of multiclade rAd5 HIV vaccines, Envelope A (EnvA) was selected as the standard antigen for a series of prototype HIV vaccines to compare various vaccine platforms. In addition, prior studies of rAd5-vectored vaccines suggested pre-existing human immunity may be a confounding factor in vaccine efficacy. rAd35 is less seroprevalent across human populations and was chosen for testing alone and in combination with a rAd5-EnvA vaccine in the present two-part phase I study.

**Methods:**

First, five subjects each received a single injection of 10^9^, 10^10^, or 10^11^ particle units (PU) of rAd35-EnvA in an open-label, dose-escalation study. Next, 20 Ad5/Ad35-seronegative subjects were randomized to blinded, heterologous prime-boost schedules combining rAd5-EnvA and rAd35-EnvA with a three month interval. rAd35-EnvA was given at 10^10^ or 10^11^ PU to ten subjects each; all rAd5-EnvA injections were 10^10^ PU. EnvA-specific immunogenicity was assessed four weeks post-injection. Solicited reactogenicity and clinical safety were followed after each injection.

**Results:**

Vaccinations were well tolerated at all dosages. Antibody responses measured by ELISA were detected at 4 weeks in 30% and 50% of subjects after single doses of 10^10^ or 10^11^ PU rAd35, respectively, and in 89% after a single rAd5-EnvA 10^10^ PU injection. EnvA-specific IFN-γ ELISpot responses were detected at four weeks in 0%, 70%, and 50% of subjects after the respective rAd35-EnvA dosages compared to 89% of subjects after rAd5. T cell responses were higher after a single rAd5-EnvA 10^10^ PU injection than after a single rAd35-EnvA 10^10^ PU injection, and humoral responses were low after a single dose of either vector. Of those completing the vaccine schedule, 100% of rAd5-EnvA recipients and 90% of rAd35-EnvA recipients had both T cell and humoral responses after boosting with the heterologous vector. ELISpot response magnitude was similar in both regimens and comparable to a single dose of rAd5. A trend toward more robust CD8 T cell responses using rAd5-EnvA prime and rAd35-EnvA boost was observed. Humoral response magnitude was also similar after either heterologous regimen, but was several fold higher than after a single dose of rAd5. Adverse events (AEs) related to study vaccines were in general mild and limited to one episode of hematuria, Grade two. Activated partial thromboplastin time (aPTT) AEs were consistent with an *in vitro* effect on the laboratory assay for aPTT due to a transient induction of anti-phospholipid antibody, a phenomenon that has been reported in other adenoviral vector vaccine trials.

**Conclusions:**

Limitations of the rAd vaccine vectors, including the complex interactions among pre-existing adenoviral immunity and vaccine-induced immune responses, have prompted investigators to include less seroprevalent vectors such as rAd35-EnvA in prime-boost regimens. The rAd35-EnvA vaccine described here was well tolerated and immunogenic. While it effectively primed and boosted antibody responses when given in a reciprocal prime-boost regimen with rAd5-EnvA using a three-month interval, it did not significantly improve the frequency or magnitude of T cell responses above a single dose of rAd5. The humoral and cellular immunogenicity data reported here may inform future vaccine and study design.

**Trial Registration:**

ClinicalTrials.gov NCT00479999

## Introduction

Despite three decades of research, the acquired immunodeficiency syndrome (AIDS) pandemic continues. Development of an effective human immunodeficiency virus (HIV) vaccine remains an urgent global health priority. The modest degree of protection reported in the RV144 vaccine trial demonstrated proof-of-concept that a vaccine can offer some protection against HIV infection [1]. Based upon subsequent analysis of the those results, which specified that the antibody component of the vaccine response was an important mediator of protection [2], the attention among the HIV vaccine field has been refocused on the quality and profile of antibody responses elicited by candidate vaccines. However, T cell-mediated immunity is still considered a useful complement to antibody neutralization for clearing HIV-infected cells and controlling HIV viral loads. Therefore, an important aspect of effective HIV vaccine development includes the creation of T cell immunogens delivered by recombinant vectors that can both elicit CD8 T cell responses and provide CD4 T cells capable of priming antibody responses. Thoughtfully designed recombinant adenovirus vectored vaccines have the potential to elicit such broad humoral and cellular immune responses [3, 4].

Adenovirus-based vaccines produce broad antibody responses and potent cellular immunity, and the Vaccine Research Center (VRC) has developed a recombinant adenoviral-vector (rAd) serotype-5-based preventive HIV vaccine that has been studied in numerous Phase I and II trials [3–7]. The utility of rAd5 vectors has been limited by the prevalence and magnitude of pre-existing, vector-specific neutralizing antibodies (NAbs), particularly in sub-Saharan Africa and Southeast Asia [8], which may reduce vaccine immunogenicity [3, 9]. Although two HIV vaccine efficacy studies employing rAd5 vaccines did not demonstrate efficacy in prevention of HIV [10–13], recombinant, non-replicating vaccine vectors remain of interest for the development of HIV vaccines, contingent upon development of methods to overcome known limitations of the rAd vector platforms. One such method is a prime-boost strategy with heterologous vectors, combining a low seroprevalent adenoviral serotype vector with another rAd vector that may or may not need to be a low seroprevalent serotype. Previous work has shown that vectors derived from immunologically distinct adenoviral serotypes may be able to overcome the suppressive effects of pre-existing anti-Ad immunity [14–16].

Previously, preclinical studies with adenoviral vector vaccines have included an observation of transient, mild elevations in the activated partial thromboplastin time (aPTT) noted in vaccinated rabbits compared to controls [17]. aPTT is a measure of blood clotting, specifically the intrinsic and common pathways of coagulation. A prolongation in the time of clot formation in this in vitro reaction suggests an individual has either a deficiency of one of several clotting factors or the presence of a clotting inhibitor, such as an antibody against a clotting factor. The normal range of aPTT depends upon the specific laboratory performing the test, but generally falls between 25 and 37 seconds. A contemporaneous Phase I clinical trial of the VRC’s Ebola-rAd5 vaccine observed that two subjects had prolonged aPTT measurements noted after vaccination, that were determined to be due to the transient induction of an anti-phospholipid antibody (APA) that interacts *in vitro* with the accelerant used to activate the partial thromboplastin time reaction [18]. This phenomenon has been reported previously in a study of an adenoviral vector product studied for the treatment of prostate cancer [19], and in both that trial and the series of Ebola-rAd5 vaccine trials performed at the VRC, there have been no clinically significant findings associated with this *in vitro* artifact [20, 21]. Although aPTT is not routinely performed in HIV vaccine studies, and was not required for evaluation of safety, post-vaccination aPTT assessments were added to VRC 012 by amendment prior to the beginning of Part II of the study. This would confirm whether the preclinical and clinical aPTT changes reported with other adenoviral vector vaccines would be observed in the present study, characterize the severity and duration of the phenomenon, and confirm that there were no clinically relevant consequences.

In 2005, the AIDS Vaccine Research Subcommittee (AVRS), chose a single antigen to carry forward in iterative vaccine development in order to facilitate the comparison of various vaccine vectors without the expense and time required to test multiple antigens iteratively [22]. Prior evaluation of a multiclade rAd5 HIV vaccine demonstrated EnvA immunogenicity and ease of manipulation [3–7]. Therefore, EnvA was selected as the gene-based antigen to evaluate a serotype 35 adenoviral vaccine vector (rAd35-EnvA) prototype in combination with rAd5 (rAd5-EnvA). The vaccine component described in the present study, an experimental HIV-1 EnvA-expressing recombinant human adenovirus B, rAd35, was one of the first of these alternative adenovirus serotypes to be studied in humans.

The VRC 012 study was designed to evaluate a prototype vaccine strategy using a then-novel rAd35 with the single encoded HIV-1 antigen, EnvA. The rAd35-EnvA vaccine was evaluated alone in a dose escalation evaluation (Part I) and then as a prime or boost for rAd5-EnvA (Part II), in seronegative adults. The study was the precursor to two recently published clinical trials, HVTN 077 and 083, that used the same vaccines with similar prime/boost regimens [23, 24]. Here, we report the results of the first Phase I study of this rAd35-EnvA HIV vaccine, evaluated alone and in a heterologous prime-boost combination with a rAd5 vector expressing the same vaccine antigen.

## Methods

### Study design

The protocol for this trial and supporting CONSORT checklist are available as supporting information; see S1 CONSORT Checklist and S1 Protocol.

VRC 012 (NIH 07-I-0167, NCT00479999) was a single-site, Phase I, two part study to examine dose, safety, tolerability and immunogenicity of a rAd35 prototype vaccine encoding for an HIV-1 envelope sequence from clade A (EnvA). This study was conducted at the National Institutes of Health (NIH), Bethesda, MD by the Vaccine Research Center (VRC) and approved by the National Institute of Allergy and Infectious Diseases Institutional Review Board (IRB) and was performed in accordance with 45 CFR Part 46, U.S. Food and Drug Administration regulations for investigational products. All subjects provided written informed consent. Study subjects were healthy adults, aged 18–50, recruited through an IRB-approved screening protocol (NIH 02-I-0127, NCT 00031304) with informed consent to be screened for an HIV vaccine clinical trial. All participants were tested for Ad35 antibody within 12 weeks of enrollment and, if enrolled in Part II, were tested for Ad5 antibody as well within the same timeframe. Funding for the conduct of this clinical trial was provided by the National Institute of Allergy and Infectious Diseases (NIAID) intramural research program.

In Part I, three open-label dosage groups of five participants each were sequentially enrolled. Subjects in Groups one, two, and three received one injection of rAd35-EnvA at 10^9^ particle units (PU), 10^10^ PU and 10^11^ PU, respectively and were followed for 24 weeks with no long-term follow-up requirements. In Part II, 20 subjects enrolled in Group four were blindly randomized to subgroups A, B, C, or D, and received a heterologous prime-boost vaccination series with a twelve week interval, as shown in the vaccination schema Table 1. The randomization sequence was obtained by computer-generated random numbers and provided to the study pharmacist by the statistician. The pharmacist and the statistician were responsible for maintaining security of the treatment assignments. To maintain blinding, any discussion of the treatment assignment between the VRC clinicians and the pharmacy staff or statistician was prohibited until after the assignments were permitted to be known to all. To decrease the potential for participant dropouts during the period between randomization and initial vaccination, randomization occurred on Day 0 after the study consent was signed and eligibility was confirmed. The study number was assigned through completion of the eligibility checklist in the electronic study database and was the next sequential number in the study number sequence. The Group four assignments were unblinded when safety data collection through 4 weeks after the booster injection for all subjects were completed. Full details of sample size determination and power to detect serious adverse events may be found in the clinical protocol located in the supplementary information, S1 Protocol.

**Table 1:** Study Schema.

Part I: Open Label Sequentially Enrolled Dose Escalation			
	**N**	Day 0 **rAd35-EnvA** VACCINATION	
Group 1	5	10^9^ PU IM	
Group 2	5	10^10^ PU IM	
Group 3	5	10^11^ PU IM	
Total	15		
Part II: Heterologous Prime-Boost Randomized and Completed Injection Schedules			
	**Randomized/Completed**	Day 0 **PRIME**	Week 12 (-7/+21 days) **BOOST**
Group 4A	5/5	**rAd35-EnvA** 10^10^ PU IM	**rAd5-EnvA** 10^10^ PU IM
Group 4B	5/3	**rAd5-EnvA** 10^10^ PU IM	**rAd35-EnvA** 10^10^ PU IM
Group 4C	5/5	**rAd35-EnvA** 10^11^ PU IM	**rAd5-EnvA** 10^10^ PU IM
Group 4D	5/5	**rAd5-EnvA** 10^10^ PU IM	**rAd35-EnvA** 10^11^ PU IM
Total	20/18	First 10 subjects blindly randomized to 4A or 4B; second 10 subjects blindly randomized to 4C or 4D.	

All rAd5-EnvA injections contained 10^10^ PU administered intramuscularly (IM). rAd35-EnvA was given at 10^10^ or 10^11^ PU to ten subjects each, also IM. The Part II follow-up plan included clinic visits through 52 weeks after enrollment, with subsequent annual contact through five years after enrollment. The primary endpoint was safety and tolerability of the different doses of rAd35-EnvA alone and in combination with rAd5-EnvA. Secondary objectives included evaluating the magnitude and frequency of the immune responses to the heterologous prime-boost vaccine regimen as measured by intracellular cytokine staining, ELISpot, vaccine antigen-specific ELISA, neutralization assays, and neutralizing antibody titers to Ad5 and Ad35 at Study Weeks four and 16.

Clinical safety evaluations included laboratory tests, physical assessments and solicited reactogenicity reports via diary card for 5 days post injection. Research blood samples were collected at enrollment, baseline, and Weeks four, 12, and 24 for Groups one-three (Part I), and for Group four at enrollment, baseline, Weeks four, 12, 16, 24, 36, 52, then yearly to five years.

### Vaccines

The same gp140(A) gene construct that was used in the rAd5 product VRC-HIVADV014-00-VP [3, 25], was used in the construction of both the rAd5-EnvA and rAd35-EnvA adenoviral vector vaccines in this study, as previously described [26]. In brief, the sequence from 92rw020 clade A strain (CCR5-tropic, GenBank accession number U08794) was used to create the synthetic gp140 versions of the clade A envelope gene truncated at the transmembrane domain of gp41. The cleavage site and fusion peptide at the junction of envelope gp120 and gp41 regions were deleted, and a portion of the interspace between the two heptad repeat regions in gp41 was deleted. The rAd35 adenovector (VRC-HIVADV027-00-VP) consisted of the Ad35 genome with a deletion of the E1 region, rendering the adenovector replication-deficient. The rAd5 adenovector (VRC-HIVADV038-00-VP) was similarly altered. Vaccines were tested in compliance with good manufacturing practices before release for use in clinical trials.

### T cell and antibody assays

Peripheral blood mononuclear cells (PBMC) were isolated for cryopreservation within six hours of each blood draw. All T cell assays were performed on cryopreserved cells. The antibodies, peptides, and methods used for cell stimulation have been previously reported [7]. Intracellular cytokine staining (ICS), IFN-γ ELISpot analysis, and analysis of serum antibody levels by ELISA were performed and analyzed by previously reported methods using stimulation with peptide pools (15-mers overlapping by 11) matched to the EnvA vaccine antigen, also as previously described [7].

### Statistical Methods

All data from participants who received at least one vaccination were analyzed. The analyses used for the primary objectives of safety and tolerability were solely descriptive, with percentages and exact confidence intervals reported. For the secondary analyses for immunogenicity, we used assay-specific pre-defined positivity criteria to categorize each individual as a responder or non-responder at each time point. For ELISA, a positive response was defined as any end point titer of 30 or greater, after raw OD correction based upon pre-vaccination samples. Volunteer sera were tested in duplicate serial 3-fold dilutions covering a dilution range from 1:30 to 1:21870. End point titers were based upon the mean sample OD for each dilution, which were corrected for the mean OD of the same dilution of the preimmunization sample. Endpoint titers for each sample/antigen were established as the last dilution with a pre-immunization corrected OD > 0.2. For ELISpot, a response was defined to be peptide-stimulated number of spots per million of at least 59 and greater than four fold above background. For ICS, a positive response was defined to be one in which the proportion of positive cells was statistically higher in the peptide-stimulated condition as compared to the background-stimulated condition by a one-sided Fisher’s exact test, and the difference in the percentages was at least 0.05% for all CD4 cytokine producers and IL-2 producing CD8s, and at least 0.08% for IFN-γ and TNF-α producing CD8s.

Analyses of immunological data included descriptive statistics as well as comparisons between the randomized subgroups of Group four. Between group comparisons use Fisher’s Exact Test for binary data and Wilcoxon Rank Sum tests for the magnitude of the response. Statistical analyses were performed using SAS 9.2 and R 3.1.1.

## Results

### Study conduct and population

**Fig 1:**
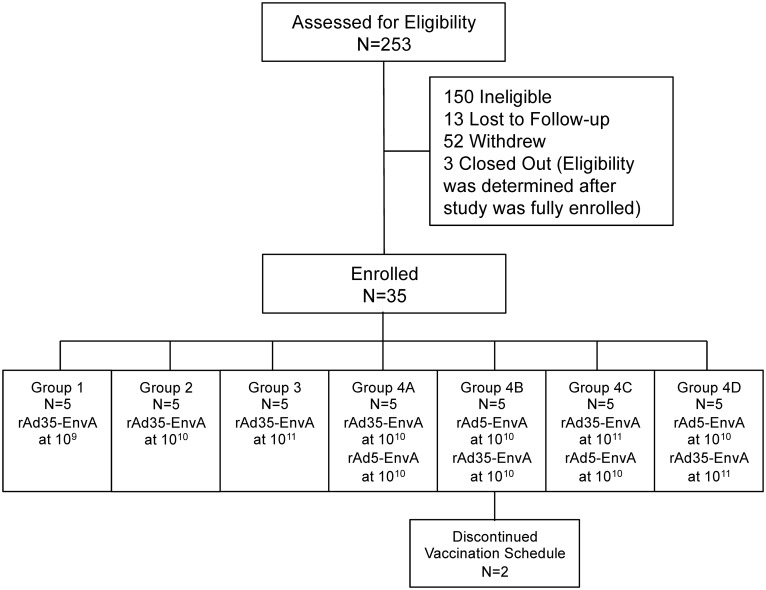
CONSORT Flow Diagram. Number of individuals assessed for eligibility, enrolled and followed up. In Part I of the study, the first 15 subjects were enrolled into the rAd35-EnvA dose escalation cohorts and in Part II, the last 20 subjects were randomized to the blinded prime-boost regimens.

The 15 subjects in Part I were enrolled between June 18, 2007 and March 31, 2008. The last Part I visit occurred September 15, 2008. The 20 subjects in Part II were enrolled between December 1, 2008 and January 6, 2010. The last Part II visit occurred 1/5/2011. The trial ended at the completion of the last long-term follow-up contact on 1/24/2014. Among the 15 subjects enrolled in Part I, all received the single planned study injection, and 14 completed the protocol through Week 24; one subject was lost to follow-up after the Study Week two visit (Table 1). Among the 20 subjects in Part II, 18 completed both planned study injections on their schedules and two received a prime but not a boost injection. Both subjects were in Group 4B and, therefore, neither received the 10^10^ rAd35-EnvA boost injection (Table 1). Neither participant discontinued due to adverse reaction. Fig 1 shows the disposition of subjects.

### Demographics

Study subject demographic characteristics are shown in Table 2. The 35 study enrollments included 14 (40%) male and 21 (60%) female subjects, with a mean age of 29.7 years (range 18 to 49 years). In addition to the 23 (66%) white subjects, eight (23%) were black or African American, one was American Indian/Alaskan native, one was Asian, and two were multiracial. All subjects were Non-Hispanic/Latino.

**Table 2:** Subject Demographics.

VRC 012 Subject Demographic Characteristics		Part I: Dose Escalation (N = 15)		Part II: Prime/Boost (N = 20)		Overall (N = 35)	
Gender	Male, N (%)	9	(60)	5	(25)	14	(40)
	Female, N (%)	6	(40)	15	(75)	21	(60)
Age	Years, Mean [Range]	27.7	[18, 49]	31.2	[23,46]	29.7	[18,49]
Race	American Indian/Alaskan Native, N (%)	0	(0)	1	(5)	1	(2.9)
	Asian, N (%)	1	(6.7)	0	(0)	1	(2.9)
	Black or African American, N (%)	4	(26.7)	4	(20)	8	(22.9)
	White, N (%)	8	(53.3)	15	(75)	23	(65.7)
	Multiracial, N (%)	2	(13.3)	0	(0)	2	(5.7)
Ethnicity	Non-Hispanic Latino, N (%)	15	(100)	20	(100)	35	(100)
	Hispanic/Latino, N (%)	0	(0)	0	(0)	0	(0)
B.M.I.	Kg/m^2^, Mean [Range]	25.3	[19.3, 32.7]	25.3	[19.0, 39.1]	25.3	[19.0, 39.1]

### Vaccine Safety

**Fig 2:**
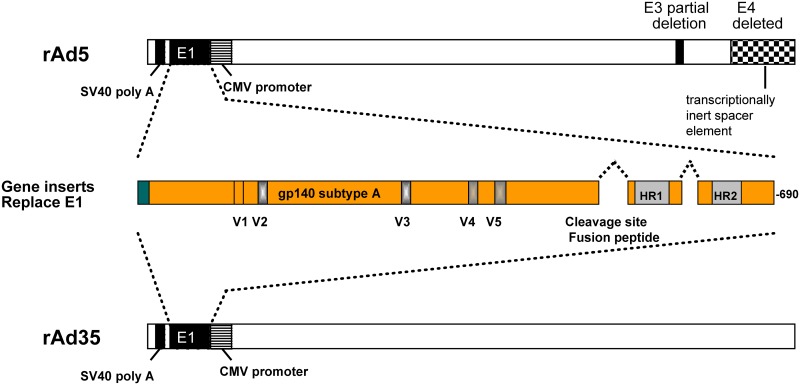
Schema of gp140(A) Gene Construct. The sequence from 92rw020 clade A strain (CCR5-tropic, GenBank accession number U08794) was used to create the synthetic gp140 versions of the clade A envelope gene truncated at the transmembrane domain of gp41. The cleavage site and fusion peptide at the junction of envelope gp120 and gp41 regions were deleted and a portion of the interspace between the two heptad repeat regions in gp41 was deleted. A portion of the E1 region was deleted from both vectors, rendering them replication-deficient.

The vaccine construct is depicted in Fig 2. Vaccinations were well tolerated. Across all subjects, the worst severity of local reactogenicity was reported as none by ten (28.6%) subjects, mild by 24 (68.6%) and moderate by one (2.9%), while the worst severity of systemic reactogenicity was reported as none by 17 (48.6%) subjects, mild by 15 (42.9%) and moderate by three (8.6%), as shown in Table 3 and S1 and S2 Tables. Each subject was counted once at worst severity for any reactogenicity parameter on five-day solicited diary cards. The top section of Table 3 summarizes across local parameters (pain/tenderness, erythema, induration). The lower section of Table 3 summarizes across systemic parameters (malaise, myalgia, headache, chills, nausea, fever). Some of the subjects who experienced systemic symptoms had a pattern of acute flu-like symptoms (malaise, myalgia, headache, chills) in the first 24 hours after injection with improvement over the next few days, consistent with that observed in previous adenoviral vector vaccine studies, although only two of 35 (5.7%) recorded a mildly elevated temperature. The three occurrences that were reported as moderate in severity were in association with rAd5-EnvA boosts. In general, rAd35-EnvA was perceived to be clinically less reactogenic than rAd5.

**Table 3:** Frequency of Local and Systemic Reactogenicity Following Injections.

	*Day 0030*	*Day 0*	*Day 0*	*Day 0*	*Week 12*	*Week 12*	*Week 12*	*All Subjects*
	*rAd35-EnvA 10*^*9*^	*rAd35-EnvA 10*^*10*^	*rAd35-EnvA 10*^*11*^	*rAd5-EnvA 10*^*10*^	*rAd35-EnvA 10*^*10*^	*rAd35-EnvA 10*^*11*^	*rAd5-EnvA 10*^*10*^	
*Symptoms Intensity*	*(N = 5)*	*(N = 10)*	*(N = 10)*	*(N = 10)*	*(N = 3)*	*(N = 5)*	*(N = 10)*	*(N = 35)*
**ANY LOCAL SYMPTOM (Pain/Tenderness, Swelling, Redness)**								
None	4 (80.0%)	6 (60.0%)	1 (10.0%)	2 (20.0%)	0 (0.0%)	1 (20.0%)	2 (20.0%)	10 (28.6%)
Mild	1 (20.0%)	4 (40.0%)	9 (90.0%)	8 (80.0%)	3 (100.0%)	4 (80.0%)	7 (70.0%)	24 (68.6%)
Moderate	0 (0.0%)	0 (0.0%)	0 (0.0%)	0 (0.0%)	0 (0.0%)	0 (0.0%)	1 (10.0%)	1 (2.9%)
**ANY SYSTEMIC SYMPTOM (Malaise, Myalgia, Headache, Chills, Nausea, Temperature)**								
None	3 (60.0%)	7 (70.0%)	5 (50.0%)	7 (70.0%)	1 (33.3%)	3 (60.0%)	5 (50.0%)	17 (48.6%)
Mild	2 (40.0%)	3 (30.0%)	5 (50.0%)	3 (30.0%)	2 (66.7%)	2 (40.0%)	2 (20.0%)	15 (42.9%)
Moderate	0 (0.0%)	0 (0.0%)	0 (0.0%)	0 (0.0%)	0 (0.0%)	0 (0.0%)	3 (30.0%)	3 (8.6%)

There were two serious adverse events during the study, neither of which was deemed related to the study vaccines. One was a case of breast cancer (described as moderately aggressive, BRCA negative, estrogen and progesterone receptor positive) in a 38-year-old female participant diagnosed 281 days after the second study injection and subsequently treated with outpatient lumpectomy, radiation and Tamoxifen. The other was a case of ductal carcinoma *in situ* in a 39-year-old female participant diagnosed 72 days after the second study injection and subsequently treated with mastectomy.

Transient prolonged aPTT was noted after five vaccinations in four of the 25 subjects in Part II, as shown in S1 Fig.[27] Three events occurred after rAd5-EnvA and two after rAd35-EnvA. In three of the four subjects, the aPTT elevation reached a grade one event and resolved within 14 days. The fourth subject experienced an initial grade one elevation in the aPTT after the rAd35-EnvA vaccination and then experienced a recurrence, with a grade three prolongation of aPTT, the highest recorded at 57.9 sec, after rAd5-EnvA boost and then slow normalization over the subsequent five months. This phenomenon has been documented before in a prior study evaluating another rAd5 vector [19] where the anti-phospholipid antibody response creates an artifact in the *in vitro* aPTT analysis. In the current study, the bleeding time and other coagulation parameters were not affected by rAd immunization. The single episode of grade 2 hematuria noted in a subject 14 days after rAd35-Env prime occurred a subject who did not have prolonged aPTT. The hematuria was noted on a single urinalysis and resolved without intervention.

One of 15 subjects in Part I and 16 of 20 in Part II had one or more test results indicating the presence of vaccine-induced seropositivity (VISP). These widely used clinical diagnostic HIV serological tests that measure antibody to HIV, including antibody to the vaccine antigen used in this trial, reverted to negative over the course of 9–12 months. A positive result on such a test should be interpreted as a false-positive and the subject’s HIV status confirmed by PCR. An example of the time course of VISP is shown as S2 Fig. All of the subjects remained HIV-negative by diagnostic PCR testing throughout the study.

### Vaccine-induced antibody responses

**Fig 3:**
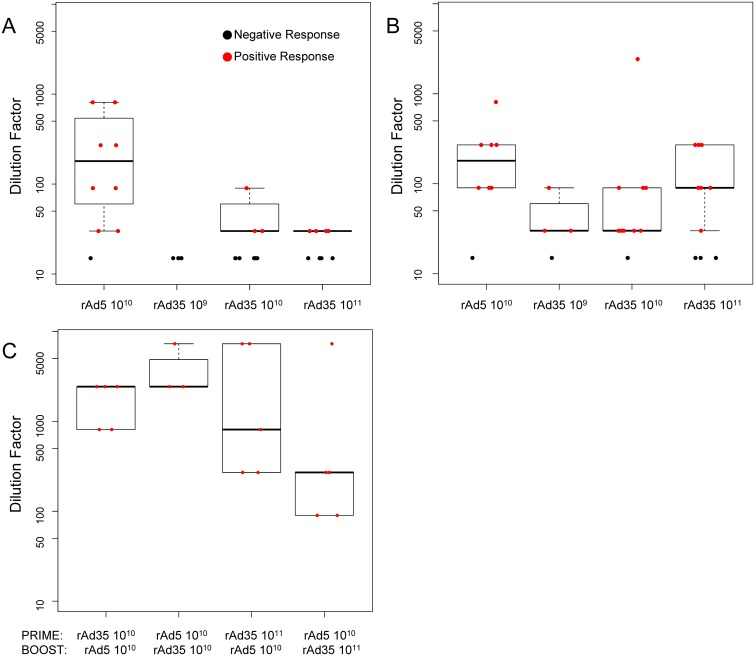
ELISA Responses Against EnvA Measured At Two Timepoints Post Prime and Four Weeks Post Boost. Endpoint titers/dilution were calculated after priming by vaccine and dose indicated on the X-axis (A & B), combined from Parts I and II, and each individual prime-boost combination indicated on the X-axis from Part II (C). Part A shows data 4 weeks post prime, while 4B shows data 12 weeks post prime. Part C shows data 4 weeks post boost. The responders are represented on the plot with red dots and are used to construct the boxplots showing the interquartile range; black dots represent the non-responders and are not included in the response range.

EnvA-specific antibody (Ab) responses measured by ELISA were detected at four weeks in 30% and 50% of subjects after single doses of 10^10^ or 10^11^ rAd35-EnvA, respectively, and in 89% after a single rAd5-EnvA 10^10^ PU injection, combining results from all subjects across parts 1 and 2 of the study. The magnitude of these Ab responses was low after a single dose of either vector and are shown in Fig 3A. At 12 weeks, positive ELISA responses were detected in 3/4 (75%), 9/10 (90%), and 7/10 (70%) subjects after single doses of 10^9^, 10^10^, or 10^11^ PU of rAd35-EnvA, respectively, as shown in Fig 3B. When measured after boosting, positive titers were seen in 5/5 (100%) subjects in Group 4A, 3/4 (75%) in Group 4B, 5/5 (100%) in Group 4C, and 5/5 (100%) in Group 4D, shown in Fig 3C.[28]

### Vaccine-induced T cell responses

**Fig 4:**
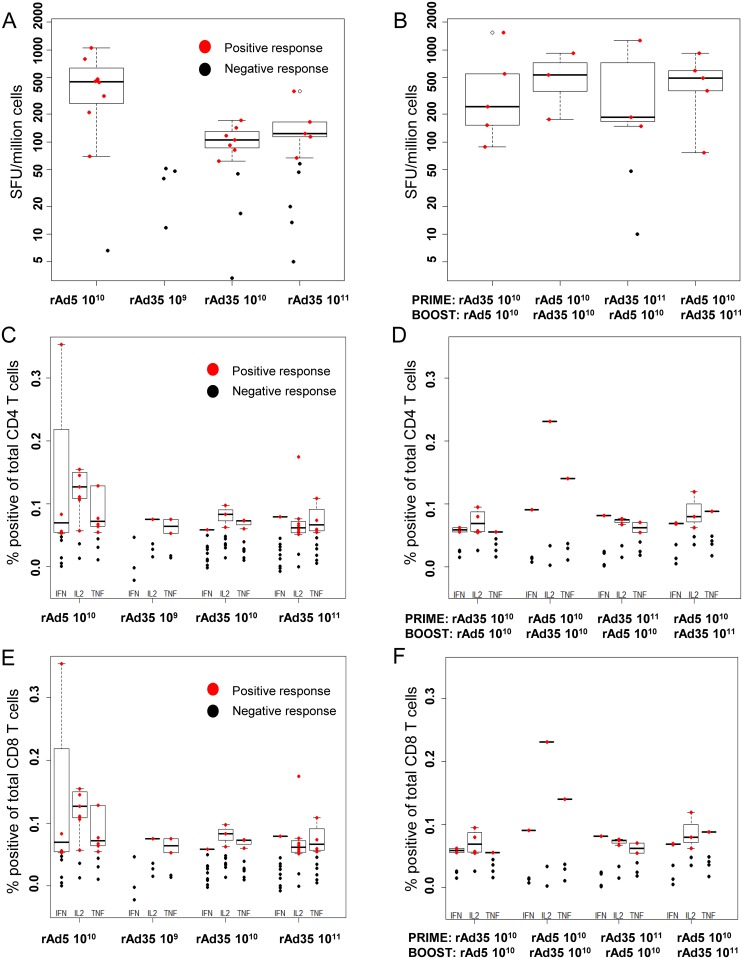
ELISpot and Intracellular Cytokine Staining for CD4 and CD8 T cell Responses Measured Four Weeks Post Prime and Boost. T cell assays were measured after priming by vaccine and dose indicated on the X-axis, combining subjects from Parts I and II in the left panels, and each individual prime-boost combination indicated on the X-axis from Part II in the right panels. A & B show ELISpot results in spot forming units (SFU) per million cells. The lower panels represent intracellular cytokine staining of CD4 T cells (C & D), and CD8 T cells (E & F) for the indicated cytokines, and are reported in percentage of positive cells of total CD4 or CD8 positive T cells. Analysis was performed on samples from 4 weeks post injection (prime or boost). The responders are represented on the plot with red dots and are used to construct the boxplots showing the interquartile range; black dots represent the non-responders and are not included in the response range.

T cell responses as measured by ELISpot were higher after a single rAd5-EnvA injection than after a single rAd35-EnvA injection at any dose, as shown in Fig 4A. IFN-γ ELISpot responses were detected in 0/5 (0%), 7/10 (70%), and 5/10 (50%) of subjects after single doses of 10^9^, 10^10^, or 10^11^ rAd35-EnvA, respectively, compared to 8/9 (89%) of subjects after a single rAd5-EnvA 10^10^ injection. T cell responses were detected in the majority of Group four rAd35-EnvA or rAd5-EnvA recipients after boosting with the heterologous vector (16/18, 89%), as demonstrated in Fig 4B. ELISpot and CD4 and CD8 T cell response magnitude was similar in both regimens and comparable to a single dose of rAd5-EnvA, as demonstrated in Fig 4C–4F. CD8 T cell polyfunctional responses shown in S3 Fig suggest that rAd5-EnvA is more effective than rAd35-EnvA as a CD8 T cell prime for a given antigen. Both dose groups of rAd35, 10^10^ and 10^11^, were combined for this analysis.

## Discussion

Here we show the rAd35-EnvA vaccine VRC-HIVADV027-00-VP was well tolerated and immunogenic. While effectively priming and boosting antibody responses when given in a reciprocal prime-boost regimen with rAd5-EnvA, the rAd35-EnvA vaccine did not significantly improve the magnitude of the T cell response above a single dose of rAd5 in seronegative healthy volunteers when given at a three-month interval. HVTN 077 used these vaccines with a six-month prime/boost interval and HVTN 083 used a three-month interval, and both showed similar antibody response levels [23, 24]. After two large clinical trials failed to demonstrate protection or reduction of viral load with Ad5 vectored HIV vaccines [10–13], it is unlikely that a similar Ad5-based regimen will be carried forward into further HIV vaccine trials [29]. However, regimens including alternate serotype Ad vectors continue to be studied both in HIV and other diseases, and several regimens induce equal or greater immunogenicity than that described in the present study [30].

In this proof-of-concept study, we used the EnvA insert as a prototype antigen because it is safe, immunogenic, and easily detected in evaluation assays. In future vaccine designs, expressing an antigen that presents a native trimer conformation, more similar to what is presented on the surface of HIV, would be more likely to induce antibody responses with sufficient potency and breadth for protection. One advantage of rAd vectors is the ability to induce both innate and adaptive immune responses; future investigations of alternative boosts, including protein boosts or alternate classes of viral vectors such as poxvirus vectors including modified vaccinia Ankara (MVA), NYVAC, or avipox vectors may help optimize antibody responses to maximize the protective effect [31–36].

As a Group B Ad vector, Ad35 binds to CD46, an inhibitory complement receptor expressed on all nucleated human cells [37]. The specificity of this binding has limited the study of rAd35 vectors in animal models, which lack endogenous CD46 expression. Animals expressing chimeric CD46, particularly transgenic mice, have been shown to be susceptible to other viruses that use CD46, including human herpesvirus 6 [38] and measles [39], but require modifications that complicate studying vaccines and immunogenicity. Previous work in humans has shown that rAd35, but not rAd5, stimulates maturation of dendritic cells and high interferon (IFN) α production [40], both important components of innate immunity. Because of the ability of Ad35 to induce dendritic cell maturation and facilitate antigen presentation, as well as the shortened duration of antigen production post-vaccination compared to rAd5 vectors, rAd35 vectors have been proposed as the boost in heterologous prime-boost vaccination strategies, taking advantage of the higher precursor frequency of B cells and T cells specific for the recombinant insert [41–43]. The current study does not include a rAd5-EnvA prime-only control, or a rAd5-EnvA/rAd5-EnvA prime/boost, therefore we are unable to make a direct comparison or draw firm conclusions about the utility of rAd35-EnvA as a boost. Several other groups have tested rAd35-EnvA as a boost, some with encouraging results.

Lower seroprevalent adenoviral vectors have been employed with some success in HIV, malaria, HCV, and Ebola vaccine trials [44]. HVTN 083 employed the same rAd35/rAd5 heterologous prime boost vaccines described in the present study, and they induced improved T cell responses by ELISpot as well as T cell responses to a larger number of epitopes over homologous regimens [24]. HVTN 077 demonstrated that a DNA/rAd35-EnvA prime/boost vaccination strategy did elicit detectable immune responses in individuals with pre-existing Ad5 immunity [23]. That data complements our finding that rAd35-EnvA is immunogenic as a boost in a heterologous regimen in subjects seronegative for Ad5, and suggests that rAd35 may indeed be useful in overcoming pre-existing adenoviral immunity. Adjuvants and alternate heterologous prime/boost regimens also show promise. Ad35 vectors were employed to study immune responses to multigenic HIV-1 vaccines with or without rAd35 EnvA vaccine and with or without an adjuvanted protein vaccine in Phase I clinical trials, and induced moderate immunogenicity with some dose-related reactogenicity [45–47]. In comparing prime-boost regimens using rAd35 followed by adjuvanted protein or the opposite regimen, Omosa-Manyonyi et. al. concluded that response rates as measured by IFN-γ ELISpot were improved when rAd35 was used as the prime rather than the boost [47]. A rAd35 vector was used in Phase Ia and Ib trials of a circumsporozoite surface antigen malaria vaccine, in the USA and sub-Saharan Africa, and was modestly immunogenic in both settings [48, 49]. A series of studies employing two doses of an Ad35 TB vaccine in BCG-primed individuals demonstrated vaccine-induced multifunctional CD4 and CD8 T cell responses by intracellular cytokine staining [50–52].

In addition to these efforts with rAd35, several groups have used other lower seroprevalent rAds. A rAd26-EnvA HIV vaccine demonstrated good safety and immunogenicity in a Phase I study, eliciting both humoral and T cell responses against EnvA [53–55]. When rAd26/rAd35 heterologous regimens, employing a different rAd35 vector and EnvA insert, were evaluated in the presence or absence of pre-existing vector immunity, using rAd26 as the prime proved more effective than priming with rAd35 at producing EnvA binding ELISA titers. Both heterologous regimens produced higher EnvA ELISA titers than homologous regimens, and there were no significant differences noted between vector-naïve and vector pre-immune individuals [56]. A unique strategy to circumvent pre-existing Ad5 immunity but still reap the immunological benefits of the Ad5 response involved creating a chimeric Ad5/Ad48 HIV EnvA immunogen [57]. Hypervariable regions from Ad48 replaced corresponding Ad5 sequences, and the vaccine induced EnvA ELISA and ELISpot responses while producing higher neutralizing antibody titers to Ad48 than to Ad5. Further efforts to avoid pre-existing vector immunity included use of chimpanzee adenoviral (ChAd) vectors. A ChAd63 vaccine utilizing conserved epitopes of multiple HIV proteins was used in several prime boost regimens with DNA or MVA, and elicited broadly specific, polyfunctional CD4 and CD8 T cell responses in humans. [58] A ChAd63 vaccine was given with or without MVA boost to successfully induce high levels of CD4 and CD8 T cells specific for the preerythrocytic malaria antigen, multiple epitope thrombospondin-related adhesion protein (ME-TRAP) [59]. A ChAd3 vectored vaccine was used in a heterologous prime/boost vaccine regimen with human Ad6 to elicit potent, long-lasting CD4 and CD8 T cell responses specific for non-structural proteins from Hepatitis C Virus (HCV)[60, 61].

Part II of this study was the first time that aPTT has been routinely evaluated after vaccinations in a clinical study with HIV adenoviral vector vaccines. The aPTT prolongation observed here was consistent with an *in vitro* effect on the laboratory assay for aPTT due to a transient induction of anti-phospholipid antibody (APA) as described previously in preclinical (rabbit) toxicology studies with both Ad5 and Ad35 vector vaccines and in humans with both a rAd5 Ebola vaccine and an investigational rAd5 prostate cancer vaccine [17–19, 62]. We were able to further describe the decay of this transiently expressed antibody, and confirm that in our trial there were no clinical manifestations associated with what was operationally an *in vitro* laboratory phenomenon, and not an *in vivo* coagulation abnormality.

As this expanding body of work on lower seroprevalence adenoviral vectored vaccines demonstrates, improvements in the combination of platform and strategy have, in some cases, produced more robust humoral and cellular immune responses to candidate vaccines than those observed in the present study. However, the goal of broad and potent protection against infection with multiple pathogens, including HIV, remains elusive. Thoughtful and detailed immunologic analysis of human clinical trial data from studies such as this one may inform the further refinement of immunogens, vectors, and vaccination strategies.

## Supporting Information

S1 CONSORT Checklist (DOC)

S1 Fig Abnormal aPTT Time Course in 4 Subjects. The time course of abnormal aPTT values in four subjects shows that abnormalities peaked around two weeks post prime and boost, and had begun to return to normal two weeks later. Subjects whose aPTT values met the definition of an adverse event (AE) were followed until resolution of the AE.
(PDF)

S2 Fig Representative Time Course of Vaccine-Induced HIV Seropositivity in One Subject. Antibody responses to EnvA detected by Abbot ELISA in study subject 019, who received rAd5-EnvA prime and rAd35-EnvA 10^10^ boost. The subject developed detectable EnvA responses at day 29 post prime, which declined prior to boost, and then peaked at day 112 (28 days after rAd35-EnvA boost), then declined slowly over the next 247 days to return to a level below the assay background.
(PDF)

S3 Fig CD8 T Cell Cytokine Production Capacity. Percentage of CD8 T cells elicited from trial volunteers immunized with either (A). rAd35 prime/rAd5 boost or (B). rAd5 prime/rAd35 boost liberating 1 (green), 2 (blue) or 3 (red) cytokines (IFNγ, IL2 or TNFα) following stimulation with EnvA specific overlapping 15mer peptides. Overall, the CD8 EnvA specific immune response was higher in those individuals following prime with rAd5. Moreover, the frequency of CD8 T cells producing 1, 2 or 3 cytokines was also greater following this regimen. Subjects receiving both doses of rAd35-EnvA were combined for this analysis.
(PDF)

S1 Protocol (PDF)

S1 Table Maximum Local Reactogenicity Summary by Vaccination Type. (PDF)

S2 Table Maximum Subject Self-assessed Systemic Reactogenicity (i.e., Solicited Adverse Events) Summary by Vaccination Type. (PDF)
