# Supplementary figures and images for: Safety and Immunogenicity of a rAd35-EnvA Prototype HIV-1 Vaccine in Combination with rAd5-EnvA in Healthy Adults (VRC 012)
Source: PLoS One. 2016 Nov 15;11(11):e0166393. doi: 10.1371/journal.pone.0166393 (PMC5112788; doi:10.1371/journal.pone.0166393)

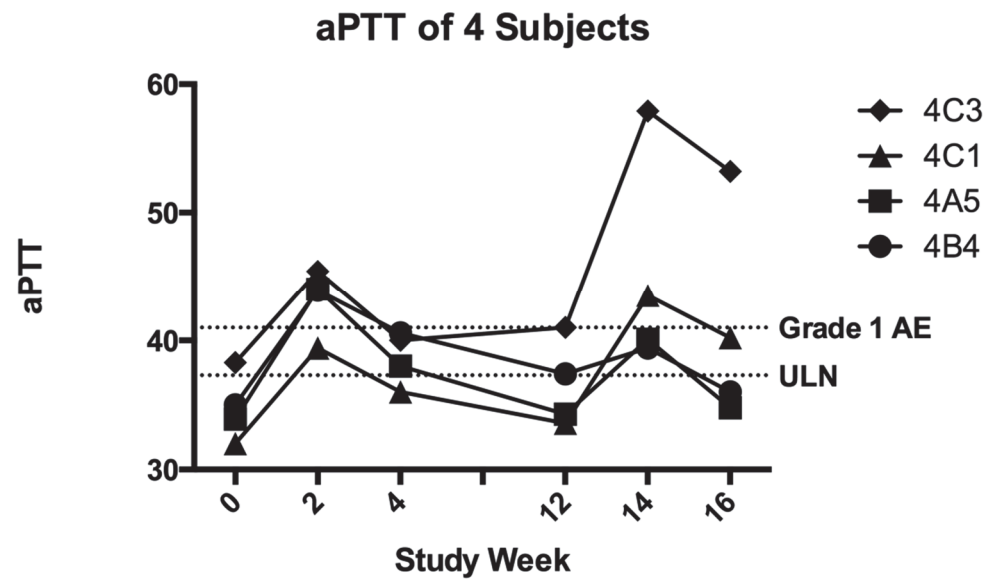

Supplement: S1 Fig — The time course of abnormal aPTT values in four subjects shows that abnormalities peaked around two weeks post prime and boost, and had begun to return to normal two weeks later. Subjects whose aPTT values met the definition of an adverse event (AE) were followed until resolution of the AE. (PDF) [file pone.0166393.s002.pdf]

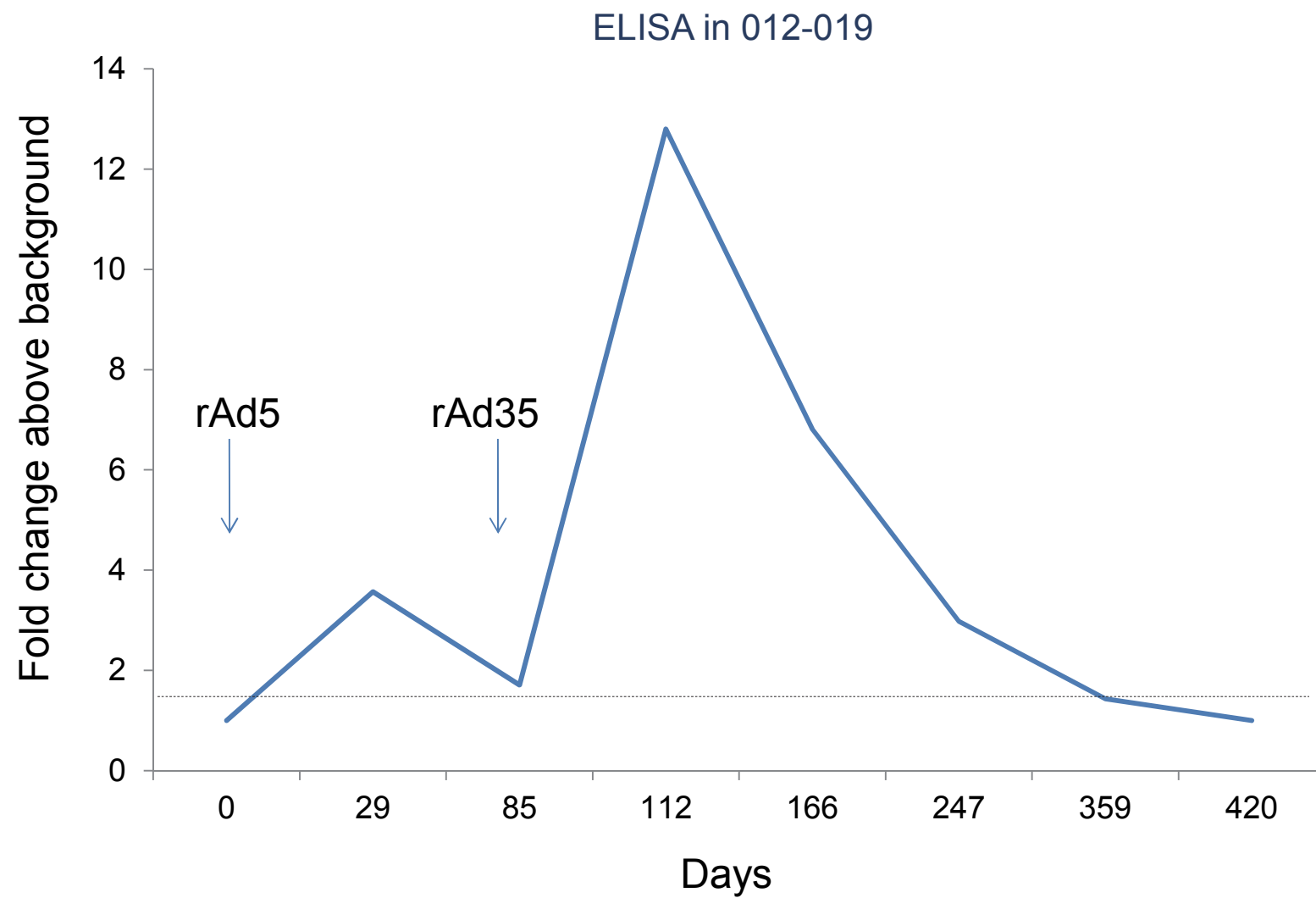

Supplement: S2 Fig — Antibody responses to EnvA detected by Abbot ELISA in study subject 019, who received rAd5-EnvA prime and rAd35-EnvA 1010 boost. The subject developed detectable EnvA responses at day 29 post prime, which declined prior to boost, and then peaked at day 112 (28 days after rAd35-EnvA boost), then declined slowly over the next 247 days to return to a level below the assay background. (PDF) [file pone.0166393.s003.pdf]

(A) Ad35 prime/Ad5 boost  
(EnvA)

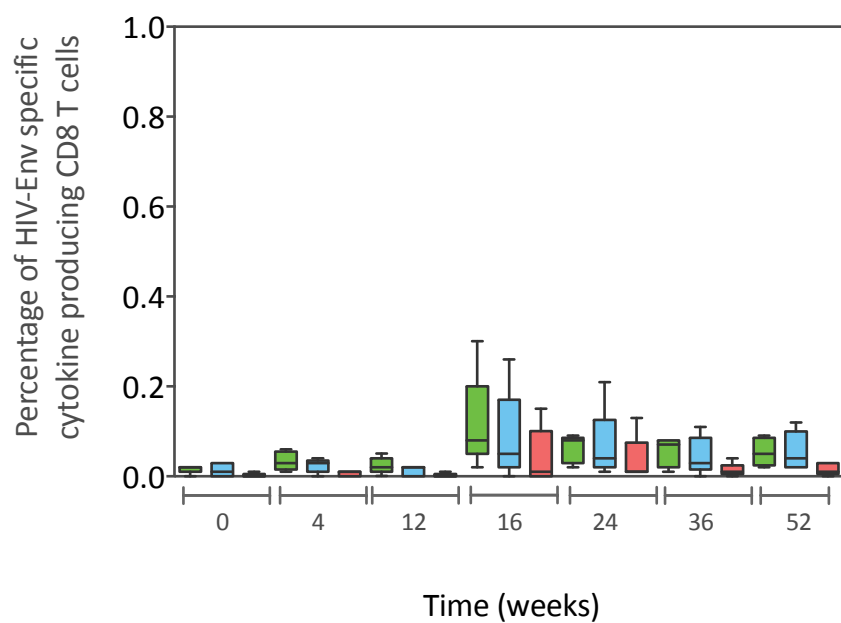

(B) Ad5 prime/Ad35 boost  
(EnvA)

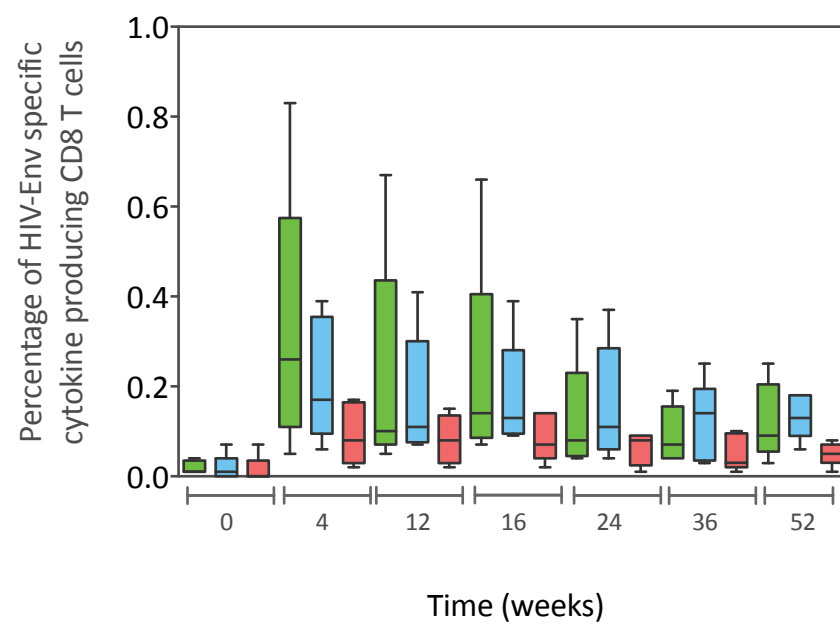

Supplement: S3 Fig — Percentage of CD8 T cells elicited from trial volunteers immunized with either (A). rAd35 prime/rAd5 boost or (B). rAd5 prime/rAd35 boost liberating 1 (green), 2 (blue) or 3 (red) cytokines (IFNγ, IL2 or TNFα) following stimulation with EnvA specific overlapping 15mer peptides. Overall, the CD8 EnvA specific immune response was higher in those individuals following prime with rAd5. Moreover, the frequency of CD8 T cells producing 1, 2 or 3 cytokines was also greater following this regimen. Subjects receiving both doses of rAd35-EnvA were combined for this analysis. (PDF) [file pone.0166393.s004.pdf]
