# Supplementary material for: Safety and Immunogenicity of a rAd35-EnvA Prototype HIV-1 Vaccine in Combination with rAd5-EnvA in Healthy Adults (VRC 012)
Source: PLoS One. 2016 Nov 15;11(11):e0166393. doi: 10.1371/journal.pone.0166393 (PMC5112788; doi:10.1371/journal.pone.0166393)
Supplement: S1 Protocol — (PDF) [file pone.0166393.s005.pdf]

Version 5.0
November 28, 2011

VACCINE RESEARCH CENTER

Protocol VRC 012
(NIH 07-I-0167)
(DAIDS-ES ID 10513)

A Phase I Clinical Trial of the Safety and Immunogenicity of an HIV-1 Adenoviral Vector Serotype 35 Vaccine, VRC-HIVADV027-00-VP (rAd35-EnvA): Dose Escalation as a Single Agent and Prime-Boost Schedules with an HIV-1 Adenoviral Vector Serotype 5 Vaccine, VRC-HIVADV038-00-VP (rAd5-EnvA), in Uninfected Adults

Vaccines Provided by
Vaccine Research Center/NIAID/NIH, Bethesda, MD

Clinical Trial Sponsored by:
National Institute of Allergy and Infectious Diseases (NIAID)
Vaccine Research Center (VRC)
Bethesda, Maryland

IND Sponsored by:
National Institute of Allergy and Infectious Diseases
Division of AIDS (DAIDS)
Bethesda, Maryland

BB-IND 13358 - *held by DAIDS*

Principal Investigator:

Barney S.Graham, M.D., Ph.D.
Vaccine Research Center, National Institute of Allergy and Infectious Diseases (NIAID)
National Institutes of Health (NIH)
Bethesda, MD 20892

IRB Initial Review Date: April 23, 2007

TABLE OF CONTENTS

Page
Abbreviations Used in VRC 012 5
Table of IND and Protocol Numbers Discussed in VRC 012 7
Précis....8

1. INTRODUCTION AND RATIONALE.... 10
1.1 HIV-1: Etiology, Disease Course, and Epidemiology 10
1.2 Rationale for the Study Vaccines and Study Plan 10
1.2.1 Adenoviral Vector Serotype 5 10
1.2.2 Adenoviral Vector Serotype 35 Selection 11
1.2.3 Study Plan 12
1.3 Previous Experience with Similar Adenoviral Vector Vaccines 13
1.4 Measures of Immunogenicity 16

2. BACKGROUND ON STUDY VACCINES.... 17
2.1 Construction of Adenoviral Vectors 17
2.1.1 Description of HIV Clade A Envelope Gene.... 17
2.1.2 VRC-HIVADV027-00-VP.... 17
2.1.3 VRC-HIVADV038-00-VP.... 18
2.2 Preclinical Safety Studies 18
2.2.1 Good Laboratory Practices (GLP) Toxicity Study in New Zealand White Rabbits 18
2.2.2 Pyrogenicity Study in Rabbits.... 20
2.2.3 GLP Biodistribution Study of VRC-HIVADV027-00-VP in Rabbits.... 20
2.3 Summary of Preclinical Immunogenicity Studies 20
2.3.1 Immune Responses to Viral Gene Products in Mice.... 21
2.3.2 Immunogenicity of Ad35-based Recombinant Adenoviral Vector Vaccines in Rhesus Monkeys 21

3. STUDY OBJECTIVES.... 22
3.1 Primary Objectives.... 22
3.2 Secondary Objectives.... 22
3.3 Exploratory Objectives 22

4. STUDY DESIGN.... 23
4.1 Study Population.... 24
4.1.1 Inclusion Criteria.... 24
4.1.2 Exclusion Criteria.... 27
4.2 Schedule of Clinical Procedures and Laboratory Evaluations.... 29
4.2.1 Screening.... 29
4.2.2 Day 0 through End of Clinical Follow-up 29
4.2.3 Long-term Follow-up Contacts for Part II Subjects:.... 32
4.3 Monitoring for HIV Infections.... 33
4.4 Intercurrent HIV Infection 33
4.5 Concomitant Medications 33
4.6 Criteria for Dose Escalation in Part I and Initiation of Part II 34

4.6.1 Part I Dose Escalation *34*
4.6.2 Initiation of Part II (Group 4) *34*
4.6.3 DSMB Reviews *34*
4.7 Criteria for Withdrawal of a Subject From the Injection Schedule 35
4.8 Criteria for Pausing Study and Resuming Study 35

5. SAFETY AND ADVERSE EVENT REPORTING 36

5.1 Adverse Events 36
5.2 Serious Adverse Events (SAE) 36
5.3 Adverse Event Reporting to the IND Sponsor 37
5.3.1 Reporting to the IND Sponsor through Study Week 24 in Part I and Study Week 52 in Part II *37*
5.3.2 Reporting to the IND Sponsor During Long-term Follow-up for Part II *38*
5.3.3 Attribution Categories *39*
5.4 Reporting to the Institutional Review Board 40
5.4.1 Adverse Event Reporting to the NIAID IRB *40*
5.4.2 Unanticipated Problem Reporting to the NIAID IRB: *40*
5.4.3 Protocol Violation Reporting to the NIAID IRB *41*
5.5 Serious Adverse Event Reporting to the Institutional Biosafety Committee 41

6. STATISTICAL CONSIDERATIONS 41

6.1 Overview 41
6.2 Objectives 41
6.3 Endpoints 41
6.3.1 Safety *41*
6.3.2 Immunogenicity *42*
6.3.3 Social Impacts *42*
6.4 Sample Size and Accrual 42
6.4.1 Randomization of Treatment Assignments *42*
6.4.2 Power Calculations for Safety *43*
6.4.3 Sample Size Calculations for Immunogenicity *44*
6.5 Statistical Analysis 44
6.5.1 Analysis Variables *44*
6.5.2 Baseline Demographics *45*
6.5.3 Safety Analysis *45*
6.5.4 Immunogenicity Analysis *45*
6.5.5 Social Impact Analysis *46*
6.5.6 Interim Analyses *46*
6.5.7 Unblinding Group 4 Assignments and Emergency Unblinding *46*

7. PHARMACY PROCEDURES 46

7.1 Study Agents 46
7.1.1 Adenoviral Vector Serotype 35 Vaccine, VRC-HIVADV027-00-VP *47*
7.1.2 Adenoviral Vector Serotype 5 Vaccine, VRC-HIVADV038-00-VP *47*
7.1.3 Diluent, VRC-DILUENT013-DIL-VP *47*
7.2 Preparation of Study Agent for Administration 47

7.2.1 Preparation of 10^9 PU Dosage of VRC-HIVADV027-00-VP for Administration by Needle and Syringe ... 48
7.2.2 Preparation of 10^{10} PU Dosage of VRC-HIVADV027-00-VP for Administration by Needle and Syringe ... 48
7.2.3 Preparation of 10^{11} PU Dosage of VRC-HIVADV027-00-VP for Administration by Needle and Syringe ... 48
7.2.4 Preparation of 10^{10} PU Dosage of VRC-HIVADV038-00-VP for Administration 48
7.3 Study Agent Labeling ... 48
7.4 Procedures to Preserve Blinding in Part II ... 48
7.5 Study Agent Accountability ... 49
7.5.1 Documentation ... 49
7.5.2 Disposition ... 49

8. HUMAN SUBJECT PROTECTIONS AND ETHICAL OBLIGATIONS ... 49

8.1 Informed Consent ... 49
8.2 Risks and Benefits ... 49
8.2.1 Risks ... 49
8.2.2 Benefits ... 50
8.3 Institutional Review Board ... 50
8.4 Protocol Registration ... 50
8.5 Subject Confidentiality ... 51
8.6 Plan for Use and Storage of Biological Samples ... 51
8.7 Subject Identification and Enrollment of Study Participants ... 52
8.7.1 Participation of Children ... 52
8.8 Compensation ... 52
8.9 Safety Monitoring ... 53

9. ADMINISTRATION AND LEGAL OBLIGATION ... 53

9.1 Protocol Amendments and Study Termination ... 53
9.2 Study Documentation and Storage ... 53
9.3 Study Monitoring, Data Collection, and Data Monitoring ... 54
9.3.1 Study Monitoring ... 54
9.3.2 Data Collection ... 54
9.4 Language ... 54
9.5 Policy Regarding Research-Related Injuries ... 54

10. REFERENCES ... 55

APPENDICES

I Study Informed Consent Form ... 59
II Contact Information ... 71
III Schedule of Evaluations ... 73
IV Table for Grading Severity of Adverse Events ... 76

Abbreviations Used in VRC 012

Abbreviation	Term
Ab	Antibody
Ad	Adenovirus
Ad5	adenovirus serotype 5
Ad35	adenovirus serotype 35
ADL	activities of daily living
AE	adverse event
A/G ratio	albumin to globulin ratio
AIDS	Acquired Immunodeficiency Syndrome
ALT	alanine aminotransferase
ANC	absolute neutrophil count
AoU	Assessment of Understanding
APA	anti-phospholipid antibody
aPTT	activated partial thromboplastin time
AST	aspartate aminotransferase
Biojector	Biojector®2000
BMI	body mass index
CAB	Community Advisory Board
CAVE	Capital Area Vaccine Effort
CBC	complete blood count
CDC	Centers for Disease Control and Prevention
cDNA	complementary deoxyribonucleic acid
cGMP	current Good Manufacturing Practices
CTL	cytotoxic T lymphocytes
DAIDS	Division of AIDS
DNA	deoxyribonucleic acid
DSMB	Data and Safety Monitoring Board
EAE	expedited adverse event
ELISA	enzyme-linked immunosorbent assay
ELISpot	enzyme-linked immunospot
Env	Envelope
FACS	fluorescence-activated cell sorter
FDA	Food and Drug Administration
FFB	final formulation buffer
GLP	Good Laboratory Practices
GMP	Good Manufacturing Practices
HBsAg	hepatitis B surface antigen
HCV	hepatitis C virus
HIV	human immunodeficiency virus
HLA	human leukocyte antigen
HVTN	HIV Vaccine Trials Network
IAVI	International AIDS Vaccine Initiative
IBC	Institutional Biosafety Committee

Abbreviation	Term
ICS	intracellular cytokine staining
IM	Intramuscular
IND	investigational new drug application
IRB	Institutional Review Board
LIMS	Laboratory Information Management System
MCB	master cell bank
MRK	Merck
NHP	non-human primate
NIAID	National Institute of Allergy and Infectious Diseases
NIH	National Institutes of Health
NSAID	nonsteroidal anti-inflammatory drug
NVITAL	NIAID Vaccine Immune T-Cell and Antibody Laboratory
PAVE	Partnership for AIDS Vaccine Evaluation
PBMC	peripheral blood mononuclear cells
PCR	polymerase chain reaction
p.i.	post immunization
Pol	polymerase
PSRT	protocol safety review team
PT	prothrombin time
PU	particle unit
rAd5	recombinant adenovirus serotype 5 vector vaccine
rAd5-EnvA	VRC-HIVADV038-00-VP (rAd5 vector encoding for clade A envelope)
rAd35	recombinant adenovirus serotype 35 vector vaccine
rAd35-EnvA	VRC-HIVADV027-00-VP (rAd35 vector encoding for clade A envelope)
RCA	replication-competent adenovirus
RCC	Regulatory Compliance Center
RPR	rapid plasma reagin
SAE	serious adverse event
SD	study day
SIV	simian immunodeficiency virus
UNAIDS	Joint United Nations Programme on HIV/AIDS
USMHRP	U.S. Military HIV Research Program
VRC	Vaccine Research Center
WBC	white blood cell
WFI	water for injection

Table of IND and Protocol Numbers Discussed in VRC 012

IND Number	Vaccine(s)	VRC Protocol Identifier	NIH Protocol Number
BB-IND 11661	VRC-HIVADV014-00-VP	**VRC 006**<br>*HVTN 054*	04-I-0172
BB-IND 11894	VRC-HIVDNA009-00-VP prime<br>VRC-HIVADV014-00-VP boost	**VRC 009**<br>*HVTN 057*<br>*HVTN 068*<br>*HVTN 069*<br>*RV 156A*	05-I-0081
BB-IND12326	VRC-HIVDNA016-00-VP prime<br>VRC-HIVADV014-00-VP boost	**VRC 008**<br>**VRC 010**<br>**VRC 011**<br>*HVTN 204*<br>*IAVI-V001*<br>*RV-172*	05-I-0148<br>05-I-0140<br>06-I-0149
BB-IND 13358	VRC-HIVDNA044-00-VP<br>VRC-HIVADV038-00-VP<br>VRC-HIVADV027-00-VP	**VRC 012**<br>*HVTN 072*	07-I-0167

Précis

Protocol VRC 012: A Phase I Clinical Trial of the Safety and Immunogenicity of an HIV-1 Adenoviral Vector Serotype 35 Vaccine, VRC-HIVADV027-00-VP (rAd35-EnvA): Dose Escalation as a Single Agent and Prime-Boost Schedules with an HIV-1 Adenoviral Vector Serotype 5 Vaccine, VRC-HIVADV038-00-VP (rAd5-EnvA), in Uninfected Adults

Study Design: The VRC recombinant adenoviral vector serotype 5 (rAd5) multiclade vaccine has been previously shown to elicit immune responses to HIV-1-specific peptides when administered intramuscularly (IM) alone and in prime-boost schedules with the greatest magnitude and frequency of response to the Envelope A immunogen (EnvA). Part I of this study is an open label, dose escalation evaluation of an HIV-1 adenoviral vector serotype 35 vaccine (rAd35-EnvA).
Subjects in Group 1 will receive one vaccination of rAd35-EnvA 10^9 PU.
Subjects in Group 2 will receive one vaccination of rAd35-EnvA 10^{10} PU.
Subjects in Group 3 will receive one vaccination of rAd35-EnvA 10^{11} PU.
Part II (Group 4) of this study is a randomized, double blind evaluation of the rAd35-EnvA vaccine in comparison to and in combination with a rAd5-EnvA vaccine in prime-boost schedules.

The hypotheses are: 1) rAd35-EnvA vaccine will be safe for human administration at dosages up to 10^{11} PU as a single agent and both the rAd35-EnvA and rAd5-EnvA vaccines will be safe in prime-boost regimens; 2) both the rAd35-EnvA and rAd5-EnvA vaccines will elicit immune responses to the EnvA immunogen; and 3) the heterologous prime-boost regimens will elicit a greater frequency and magnitude of response than after the priming vaccinations alone. The primary objectives relate to evaluation of the safety and tolerability of the rAd35-EnvA and rAd5-EnvA vaccines. Secondary objectives are related to evaluation of the immunogenicity of the vaccines when comparing rAd35-EnvA to rAd5-EnvA when administered as a prime or as a boost vaccination.

Product Description: Both the VRC-HIVADV038-00-VP (rAd5-EnvA) and the VRC-HIVADV027-00-VP (rAd35-EnvA) vaccines are composed of recombinant, replication deficient adenoviral vectors that encode for HIV-1 clade A Env glycoprotein.

Subjects: Thirty-five healthy adult volunteers, 18 to 50 years old; beginning with the Version 2.0 protocol, subjects in Part I must be Ad35 antibody (Ab) seronegative and subjects in Part II must be both Ad5- and Ad35-seronegative.

Study Plan: Part I: Fifteen subjects will receive an open-label 1 mL IM deltoid injection via needle and syringe of the study agent. No more than one subject per day will be enrolled into each dose group. Five days following vaccination of the fifth volunteer in each dose group, there will be an internal safety review including the principal investigator, clinical team and medical officer to determine whether to proceed to next dose level.

Part II: Initiation of enrollment into Part II will be contingent upon completion of enrollment into Group 3 and a safety review of 10^9 and 10^{10} PU dosage by the Data and Safety Monitoring Board (DSMB). The safety review will take place when at least 2 weeks of follow-up on the last 10^{10} PU injection in Group 2 is

available in the safety reports; the DSMB safety review may occur during enrollment of Group 3.

Enrollment into Group 4 will be randomized and double-blinded. The first 10 subjects in Group 4 will be randomized in a 1:1 ratio into heterologous prime-boost vaccination schedules in which both rAd5-EnvA and rAd35-EnvA are administered at the 10^{10} PU dosage. The last 10 subjects in Group 4 will be randomized in a 1:1 ratio into heterologous prime-boost vaccination schedules in which the rAd5-EnvA is administered at 10^{10} PU and rAd35-EnvA is administered at 10^{11} PU. All subjects will receive each study agent administered as 1 mL IM deltoid injections (12 weeks apart) according to the schedule.

PART I: Dose Escalation

Open-label; Sequentially Enrolled Groups	N =	Day 0 VACCINATION
Group 1	5	**rAd35-EnvA** (10^9 PU IM)
Group 2	5	**rAd35-EnvA** (10^{10} PU IM)
Group 3	5	**rAd35-EnvA** (10^{11} PU IM)
**TOTAL**	**15**	

PART II: Heterologous Prime-Boost Schedules

Randomized, Double-Blinded Enrollment	N =	Day 0 PRIME	Week 12 (-7 or +21 days) BOOST
Group 4A	5	**rAd35-EnvA** (10^{10} PU IM)	**rAd5-EnvA** (10^{10} PU IM)
Group 4B	5	**rAd5-EnvA** (10^{10} PU IM)	**rAd35-EnvA** (10^{10} PU IM)
Group 4C	5	**rAd35-EnvA** (10^{11} PU IM)	**rAd5-EnvA** (10^{10} PU IM)
Group 4D	5	**rAd5-EnvA** (10^{10} PU IM)	**rAd35-EnvA** (10^{11} PU IM)
**TOTAL**	**20**		

Study Duration: The vaccination regimen and clinical follow-up schedule for Part I requires 24 weeks and for Part II requires 52 weeks to complete. Part II subjects will be contacted annually for 4 years after study completion for collection of long-term follow-up information.

1. INTRODUCTION AND RATIONALE

1.1 HIV-1: ETIOLOGY, DISEASE COURSE, AND EPIDEMIOLOGY

Globally, the human immunodeficiency virus (HIV) incidence rate is thought to have reached its peak in the 1990's, with the exception of increasing incidence in a few countries. However, the number of people living with HIV continues to increase due to population growth and longer life expectancy achieved by use of antiretroviral medications. According to the Joint United Nations Programme on HIV/AIDS (UNAIDS), as of the end of 2005, it is estimated that 33.4-46.0 million people are living with HIV/AIDS, including an estimated 3.4-6.2 million new cases in 2005 [1, 2]. Worldwide there were an estimated 2.4-3.3 million deaths due to HIV/AIDS in 2005 [1, 2] and there have been as many as 30 million deaths as a result of HIV infection since the beginning of the epidemic [3].

Beyond the human tragedy of HIV/AIDS, the costs of the epidemic pose a significant impediment to the economic growth and political stability of many countries. In developing countries and in segments of the U.S. population, anti-HIV therapies are frequently beyond financial reach. Accordingly, effective, low-cost tools for HIV prevention, such as a vaccine, are urgently needed to bring the HIV epidemic under control. For this reason, the Vaccine Research Center (VRC) and Division of AIDS (DAIDS) at the National Institute of Allergy and Infectious Diseases (NIAID) of the National Institutes of Health (NIH) are committed to the development of safe, effective vaccines to prevent HIV infection and AIDS worldwide.

1.2 RATIONALE FOR THE STUDY VACCINES AND STUDY PLAN

The recombinant adenovector product vaccine design is based on the concept of immunization by gene transfer. Recombinant adenovector vaccines offer the positive attributes of immune stimulation by live attenuated vaccines, without adjuvant, and without utilizing HIV as the attenuated virus. Preclinical studies [4-9] and clinical studies [10-13] show that immune responses against HIV can be elicited by direct gene transfer of immunogen-expressing HIV genes via recombinant adenovectors. The major advantage of rAd immunization appears to be its efficacy in transducing host cells and priming the induction of $CD8^+$ cytotoxic T lymphocytes (CTL) responses, which are considered an important element in controlling HIV-1 viral replication [4, 14-20]. There is an additional safety feature in that following entry into the target cells, the HIV-1 gene products will be produced without the production of infectious adenovirus (Ad) or integration into the host genome. These gene products can be produced in cells that are not actively dividing.

1.2.1 <u>Adenoviral Vector Serotype 5</u>

The VRC has developed VRC-HIVADV014-00-VP (BB-IND 11661), a recombinant serotype 5 adenovector (rAd5) product composed of four rAd5 vectors (in a 3:1:1:1 ratio) that encode for HIV-1 Gag/Pol polyproteins from clade B and HIV-1 Env glycoproteins from clades A, B, and C, respectively. This vaccine has been evaluated as a single agent in Phase I studies (BB-IND 11661) and is now being further evaluated in Phase I (BB-IND 11894, BB-IND 12326) and Phase II (BB-IND 12326) studies in healthy human subjects as a boost following vaccination with a plasmid DNA prime vaccine. The study data obtained thus far from the clinical trials with the VRC rAd5 vaccine, as well as from the human clinical trials of rAd5-based vaccines

developed by Merck [8] suggest that rAd5 vaccines are well-tolerated and immunogenic at dosages from 10^9 through 10^{11} particle units (PU).

Vaccines using adenoviral vectors have been evaluated as a promising approach to developing an HIV vaccine that induces T cell immunity. Studies have shown that replication-deficient rAd5 vectors can generate cellular immune responses against HIV-1 and simian immunodeficiency virus (SIV) in non-human primates [4-9] and induce HIV-specific immune responses in healthy, HIV-negative human subjects [13]. A major potential limitation of rAd5 vector vaccines however, is the high prevalence of pre-existing immunity to adenovirus serotype 5 (Ad5) in human populations which may diminish vaccine-induced immune responses [4]. Interim results of the Phase IIB efficacy study of the Merck rAd5 vaccine (HVTN 502, also known as the Step Study) suggest caution in administering rAd5 vector vaccines to subjects with pre-existing Ad5 antibody (Ab) at enrollment [21]. The Step Study was designed to enroll 3000 men and women with an increased risk of exposure to HIV infection. The Step Study was halted on September 19, 2007 for futility when the interim data reviewed by the Data and Safety Monitoring Board (DSMB) indicated that the MRK-rAd5 HIV vaccine was not preventing HIV infections and was not reducing the HIV viral load in participants who became HIV-infected. An unexpected safety concern was that there were more HIV infections in male vaccinated participants who already had Ad5 Ab at the time of enrollment (from a prior Ad5 naturally occurring infection) than in the male placebo recipients from the same group. A post hoc multivariate analysis using four models indicated that the highest hazard ratio (HR) when comparing vaccine to placebo recipients was consistently found in the vaccinated men who were uncircumcised and had pre-existing immunity to Ad5 [HR= 4.2 to 4.8 (depending upon the model used)]. Although the MRK-rAd5 vaccine induces Ad5-specific Ab, the men who were Ad5-seronegative at enrollment and received vaccine did not have higher rates of HIV infection than the placebo recipients who were Ad5-seronegative at enrollment. The circumcised men who were Ad5-seronegative at enrollment had the lowest HR (0.6 to 0.8, depending upon the model used) (http://www.stepstudies.com). Further evaluations of Step Study results are ongoing to try to identify the basis for this unexpected observation. There was only one HIV infection among the women in the Step Study, therefore, there was not enough data in women to complete a multivariate analysis for women.

1.2.2 Adenoviral Vector Serotype 35 Selection

Many reports have confirmed high rates of Ad5 seroprevalence (with high levels of Ad5 neutralizing antibody), particularly in developing regions such as sub-Saharan Africa, which are most at risk for HIV infection and where the need for safe and effective vaccine is most critical [22, 23]. Therefore an active area of research is the development of adenoviral vectors engineered to evade dominant Ad5-specific immunity either because they are based upon alternative adenovirus serotypes, which are less seroprevalent [24] or because they have been genetically modified to evade suppression by anti-Ad5 immunity by constructing chimeric hexon or fiber proteins [24, 25]

The recombinant adenoviral serotype 35 vector (rAd35) prototype vaccine in this study, designated VRC-HIVADV027-00-VP, is designed to test this concept. It is not intended to be a final candidate vaccine product, but rather a test of the vector. The vector contains the wild-type Ad35 fiber protein. The encoded HIV-1 clade A envelope (EnvA) gene has previously been shown to be immunogenic when delivered as plasmid DNA vaccines to mice [26] and to non-

human primates [27] with confirmation of human immunogenicity in clinical trials of DNA plasmid [10] and rAd5 [13] vaccines that include the EnvA gene among the encoded immunogens. In the multiclade, multigene rAd5 vaccine previously studied, EnvA was associated with the greatest frequency and magnitude of immune response and was therefore selected for this prototype rAd35 vaccine.
The rAd35 vaccine has been evaluated for safety (Section 2.2) and immunogenicity (Section 2.3) in preclinical trials and relative to rAd5 preclinical study results appears to be somewhat less reactogenic. rAd35 is equally immunogenic as rAd5 in mice and slightly less potent than rAd5 in Ad5-naïve non-human primates. The preclinical studies to date support the hypothesis that rAd35 may have immunogenic advantages over rAd5 vectors in human populations with widespread Ad5 immunity.

1.2.3 Study Plan

The VRC 012 (in BB-IND 13358) study plan is to first evaluate, in a dose escalation design, the safety of the prototype rAd35-EnvA vaccine, VRC-HIVADV027-00-VP, at three dosages, 10^9 PU, 10^{10} PU and 10^{11} PU. The study will then evaluate two prototype regimens in which a recombinant adenoviral vector serotype 5 vaccine (VRC-HIVADV038-00-VP) that also encodes only for EnvA (rAd5-EnvA) is administered either as the prime for the rAd35-EnvA boost or as the boost after an rAd35-EnvA prime. In these prime-boost schedules all rAd5-EnvA injections will be at the 10^{10} PU dose, but half of the rAd35-EnvA injections will be at 10^{10} PU and half at 10^{11} PU. The intent is to compare responses between rAd5 and rAd35 products on a particle for particle basis at the dosage being taken forward for Phase II evaluation of the VRC candidate multiclade, multigene rAd5 vaccine (VRC-HIVADV014-00-VP), but also at a higher dosage of the rAd35-EnvA. The rAd35 vaccine construct has different characteristics (e.g., prevalence of natural immunity to Ad35, reactogenicity, etc) from the rAd5 vector in several important respects and therefore the study plan has taken into consideration the possibility that the optimal dosage for rAd35 vector vaccine may be higher than for rAd5 vector vaccines. It is also the case that non-human primate (NHP) studies are not necessarily predictive of human immune response. Although the NHP data (see Section 2.3.2) indicate that the sequence of rAd35-EnvA prime with rAd5-EnvA boost is associated with a greater immune response than the reverse sequence, both sequences will be evaluated in this Phase I study. If this prototype rAd35-EnvA vector vaccine is evaluated as safe for further evaluation and provides evidence of promising immunogenicity in humans, vaccines containing additional HIV genes (i.e. *gag, pol*, and *env* from additional clades) will be produced and evaluated for safety and immunogenicity in preclinical and clinical studies. Having preliminary information on both the 10^{10} PU and 10^{11} PU dosage of rAd35-EnvA in the heterologous prime-boost schedules will help inform decisions about dosages to prepare and evaluate in future studies.

The VRC 012 study was put on administrative pause by the IND Sponsor on October 19, 2007 pending further information from the Step Study (see Section 1.2.1). At the time of the VRC 012, Version 2.0 (January 8, 2008) protocol amendment, the rAd35-EnvA had been administered to 13 subjects between June 18, 2007 and October 4, 2007. This included 5 in each of the first two dosage groups (10^9 PU and 10^{10} PU) and 3 in the 10^{11} PU dosage group. Following approval of Version 2.0, the VRC 012 study reopened to enrollments in March 2008 and completed enrollment of the last 2 subject in the 10^{11} PU dosage group, thus completing the enrollment of Part I. In Part I, mild local reactogenicity was reported by 7 of 15 (46.7%) subjects and included mild pain without erythema or induration. Mild systemic reactogenicity

was reported by 7 of 15 (46.7%) subjects and included mild malaise, myalgia, headache, as well as one report of fever with chills. To date, there have been no adverse events meeting the criteria for expedited reporting or study pause.

A revised study plan to increase the number of subjects vaccinated with the 10^{11} PU dosage is supported by three factors. First, the rAd35 reactogenicity at 10^{11} PU was minimal in the five subjects vaccinated at this dosage in Part I; secondly, the 10^{11} PU dosage is considered to be feasible from a manufacturing perspective; and finally, there is a suggestion that immunogenicity may improve at the higher dosage based on preliminary data available from Part I. Interferon-γ ELISpot responses from week 4 showed that at 10^9 PU no subjects had a detectable response; at 10^{10} PU three of five had responses with a mean of 93 SFU per 10^6 peripheral blood mononuclear cells (PBMCs); and at 10^{11} PU four of five had responses with a mean of 175 SFU per 10^6 PBMCs.

The other study originally submitted to BB-IND 13358, HVTN 072, initiated enrollment on August 15, 2007. This randomized, placebo-controlled study was designed to enroll subjects with pre-existing Ad5 immunity and to evaluate four prototype regimens as follows: 1) rAd35-EnvA with rAd5-EnvA boost; 2) rAd5-EnvA with rAd35-EnvA boost; 3) three DNA-EnvA (VRC-HIVDNA044-00-VP) primes with rAd5-EnvA boost, and 4) three DNA-EnvA primes with rAd35 boost. Following release of Step Study information, this protocol was closed to enrollment. At the time of study closure, there were 12 subjects in the DNA prime-rAd boost arms and 5 in the rAd heterologous prime-boost arms. All were in the prime portion of their regimens and among the latter the prime injections included 2 rAd5-EnvA, 1 rAd35-EnvA and 2 placebo injections. A new study is being developed to evaluate rAd35-EnvA as a prototype vaccine in DNA prime-rAd boost and rAd heterologous prime-boost regimens.

1.3 Previous Experience with Similar Adenoviral Vector Vaccines

The Vaccine Research Center (VRC), NIAID, NIH, in collaboration with DAIDS, NIAID, NIH have conducted a series of clinical studies with a multiclade, multigene adenoviral vector vaccine (VRC-HIVADV014-00-VP). Phase I evaluation of the rAd5 vaccine was initiated in human clinical trials in 2004. Table 1.1 provides a summary of the clinical studies in which the VRC multiclade rAd5 vaccine has been administered either as a single agent and/or as a booster vaccination.

Table 1.1: Experience with VRC-HIVADV014-00-VP (as of August 2008)

INDs held by DAIDS, NIAID, NIH	Study	Comment
**BB-IND 11661**<br>VRC-HIVADV014-00-VP<br>(rAd5 as single agent)<br>4 adenoviral vectors with inserted plasmids<br>clade B gag-pol,<br>clade A *env*,<br>clade B *env*,<br>clade C *env*	VRC 006	STATUS: study completed.
	HVTN 054	STATUS: study completed
**BB-IND 11894**<br>VRC-HIVADV014-00-VP<br>(rAd5 as booster)<br><br>4-plasmid DNA vaccine:<br>VRC-HIVDNA009-00-VP<br>clade B gag-pol-nef,<br>clade A *env*,<br>clade B *env*,<br>clade C *env*	VRC 009	STATUS: study completed
	HVTN 057	STATUS: study completed
	RV156A	STATUS: closed to accrual; follow-up ongoing
	HVTN 068	STATUS: study completed
	HVTN 069	STATUS: fully enrolled, vaccinations discontinued, and follow-up ongoing
**BB-IND 12326**<br>VRC-HIVADV014-00-VP<br>(rAd5 as booster)<br><br>6-plasmid DNA vaccine:<br>VRC-HIVDNA016-00-VP<br>clade B *gag*,<br>clade B *pol*,<br>clade B *nef*,<br>clade A *env*,<br>clade B *env*,<br>clade C *env*	VRC 010	STATUS: study completed
	VRC 008	STATUS: study completed.
	VRC 011	STATUS: fully enrolled, follow-up ongoing
	HVTN 204	STATUS: study completed; long-term follow-up ongoing
	IAVI V001	STATUS: study completed
	RV 172	STATUS: study completed; long-term follow-up ongoing
	IAVI V002	STATUS: withdrawn
	PAVE 100	STATUS: withdrawn

<u>Single Vaccine Studies</u>: The two Phase I single vaccine dose escalation studies in BB-IND 11661 (VRC 006 and HVTN 054), together included 70 vaccinees and 14 placebo recipients. Dosages evaluated included 10^9 PU (n=10), 10^{10} PU (n=30) and 10^{11} PU (n=30). As predicted by preclinical rabbit studies, some subjects experienced local reactogenicity, fever and flu-like symptoms (headache, muscle aches, malaise, chills and/or nausea). Table 1.2 includes a summary of the reactogenicity for the 10^{10} and 10^{11} PU dose groups in VRC 006 and HVTN 054.

In the VRC 006 study (n=36; 30 vaccinees and 6 placebo recipients) conducted at the NIH

Clinical Center, local reactogenicity (pain, redness, swelling) was usually mild (grade 1). It often began within 24 hours after vaccination, but in some individuals onset of local reactions was delayed until 3-5 days after vaccination. At the 10^{11} PU dose four subjects had fever along with flu-like systemic symptoms that included headache, malaise, muscle aches and/or chills starting within 24 hours after vaccination and lasting several hours. In VRC 006, there were no serious adverse events (SAE) attributed to study vaccine.

The HVTN 054 study (n=48; 40 vaccinees and 8 placebo recipients) was the second Phase I study of the rAd5 vaccine as single agent and was conducted at a multicenter study in uninfected adenovirus-naïve adult subjects. It opened to accrual in April 2005, enrollment was completed in September 2005 and follow-up was completed in September 2006. The HVTN 054 data also indicate that there is a higher frequency and severity of local and systemic reactogenicity at the higher dose level as the 10^{11} PU dose group (n=24) was paused per Phase I protocol requirements 4 times; once for grade 3 fever and three times for grade 2 fever.

Prime-Boost Vaccination Studies: The first DNA prime-rAd5 booster study, HVTN 057 was initiated in 2004 and all booster vaccinations were at the 10^{10} PU dose. The results indicate local reactogenicity in 79% of participants and systemic reactogenicity in 43% of participants. Reactogenicity was generally mild, but sometimes moderate in severity. The most commonly reported symptoms were malaise and/or fatigue, myalgia and headache. Six (8.5%) participants reported mild fever.

Subsequently, two intramural roll-over booster studies, VRC 009 for the 4-plasmid DNA, and VRC 010 for the 6-plasmid DNA vaccine, were initiated in 2005 and the VRC 008 study (N=40) opened in May 2005. Consistent with prior studies, the booster vaccinations were frequently followed by systemic symptoms. In VRC 008, 13 of 39 (33.3%) subjects recorded a fever, including one with a transient grade 3 fever (39.9º C). Also of note is that 3 subjects had Grade 2 (>9 x 9 cm) erythema and/or induration after the rAd5 booster. Summary reactogenicity is shown in Table 1.2.

The leading candidate vaccination regimen for prevention of HIV-1 developed by the VRC is a multiclade 6-plasmid DNA prime-rAd5 booster vaccination regimen. This approach was evaluated as safe and promising enough to warrant inclusion in an international Phase I/II evaluation (referred to as the "Triad studies"), which was initiated in late 2005 through collaboration with three multicenter networks: the HIV Vaccine Trials Network (HVTN), the International AIDS Vaccine Initiative (IAVI) and the U.S. Military HIV Research Program (USMHRP) (see Table 1.1, BB-IND 12326). The final accrual was 920 subjects combined and administration of injections in these studies is complete. Potential safety concern for administration of rAd5 vaccines to subjects with pre-existing Ad5 Ab were raised by the Merck rAd5 (MRK-rAd5) vaccine in the Step Study (HVTN 502). The Merck candidate regimen involved administration of three injections of MRK-rAd5 vaccine, encoding for clade B Gag, Pol and Nef. The Phase IIB efficacy studies of MRK-rAd5 [HVTN 502 (Step Study) and HVTN 503 (Phambili Study)], were halted September 19, 2007 (see Section 1.2.1). The VRC is also evaluating the rAd5 prime-rAd5 boost approach in the extramural, multicenter study, HVTN 068 (N=66 with 30 in rAd5 prime-rAd5 boost schedules) and the intramural, single site study, VRC 011 (N=60 with 30 in rAd5 prime-rAd5 boost schedules). The reactogenicity of the VRC-rAd5 vaccine from Phase I studies is shown in Table 1.2. There are no serious adverse events attributed to the VRC-rAd5 candidate vaccine from the ongoing Phase I and Phase II studies.

Table 1.2: Reactogenicity of Multiclade HIV-1 rAd5 Vaccine in Phase I Studies

	10^{10} PU				10^{11} PU		
**rAd5 Study Dosage**	**Single Vaccine**	**Single Vaccine**	**Prime-Boost**	**Prime-Boost**	**Single Vaccine**	**Single Vaccine**	**Prime-Boost**
	**VRC 006 10^{10} PU (N=10)**	**HVTN 054 10^{10} PU or FFB* (N=24)**	**VRC 009/010 10^{10} PU (N=14)**	**VRC 008 10^{10} PU (N=19)**	**VRC 006 10^{11} PU (N=10)**	**HVTN 054 10^{11} PU or FFB* (N=24)**	**VRC 008 10^{11} PU (N=20)**
**Local Symptoms**							
None	2 (20%)	7 (29%)	0	3 (16%)	0	3 (13%)	1 (5%)
Mild	8 (80%)	12 (50%)	13 (93%)	14 (74%)	9 (90%)	13 (54%)	14 (70%)
Moderate	0	5 (21%)	1 (7%)	2 (10%)	1 (10%)	8 (33%)	5 (25%)
Severe	0	0	0	0	0	0	0
**Systemic Symptoms**							
None	4 (40%)	7 (29%)	5 (36%)	3 (16%)	1 (10%)	3 (12%)	2 (10%)
Mild	6 (60%)	10 (42%)	3 (21%)	14 (74 %)	3 (30%)	6 (25%)	7 (35%)
Moderate	0	7 (29%)	6 (43%)	2 (10%)	6 (60%)	11 (46%)	10 (50%)
Severe	0	0	0	0	0	4 (17%)	1 (5%)

* final formulation buffer (placebo)

Other Recombinant Ad35 Vaccines: Both tuberculosis and malaria vaccines, manufactured by Crucell Holland BV, utilizing a replication deficient adenovirus 35 backbone (Ad35.CS.01) are currently being evaluated in clinical studies; clinicaltrials.gov identifier: NCT00371189 describes the malaria vaccine study.

1.4 Measures of Immunogenicity

Part I of the study will provide a preliminary assessment of the immunogenicity of VRC-HIVDNA027-00-VP at three dosages and Part II will provide a preliminary assessment of the immunogenicity of VRC-HIVADV027-00-VP and VRC-HIVADV038-00-VP both alone and in heterologous prime-boost regimens at 10^{10} and 10^{11} PU by employing enzyme-linked immunospot (ELISpot) and intracellular cytokine staining (ICS) assays that evaluate CTL responses, as well as assays that evaluate HIV-specific antibody responses. Clade-A specific peptides will be used to detect T-cell responses by an ELISpot assay modified from a previously published method [28]. The ICS assay is based upon previously published methods [29] and quantitates the frequency of $CD4^+$ and $CD8^+$ cells that produce interleukin-2 and/or interferon-gamma and other functional parameters of T cell function, in response to a pool of peptides representing the HIV antigen (Env). The frequency and magnitude of HIV-specific antibodies will be evaluated using an enzyme-linked immunosorbent assay (ELISA) [30].

The ability of the vaccine to elicit neutralizing antibody against HIV-1 strains from clades A, B, and C will be evaluated by a single round of replication using an Env-pseudovirus assay with a luciferase read-out [31, 32]. The pre-vaccination and post-vaccination presence of adenovirus serotype 5 or 35 neutralizing antibody in study volunteers will be evaluated using a previously published luciferase transgene detection method [33] and evaluations of T cell responses to the adenoviral vector components may also be performed as exploratory evaluations. Other assays may also be completed from stored samples at a later date if further elucidation of immunogenicity is of interest.

2. BACKGROUND ON STUDY VACCINES

The VRC, NIAID, NIH has collaborated with GenVec, Inc. (GenVec) in Gaithersburg, MD, to develop a replication-deficient human adenovirus B serotype 35-based recombinant (rAd35) vector designed to confer an immune response against HIV. DAIDS will sponsor the IND that will support the clinical development of the rAd35 product and VRC will conduct the initial Phase I study of this adenovector in healthy volunteers.

Clinical trial material is manufactured by GenVec or a qualified contractor under current Good Manufacturing Practices (cGMPs) and United States Food and Drug Administration (FDA) procedures and requirements for investigational new drugs. The final formulation buffer, VRC-DILUENT013-DIL-VP (FFB), for the study vaccines was custom manufactured by Cambrex (Walkerville, MD). Analytical methods to assure the proper identification, quality, purity and strength of the investigational adenovectors have been qualified for product release testing for use in Phase I and Phase II clinical investigations

2.1 CONSTRUCTION OF ADENOVIRAL VECTORS

2.1.1 Description of HIV Clade A Envelope Gene

The same gp140(A) gene that was used in the rAd5 vaccine product, VRC-HIVADV014-00-VP, previously tested in a Phase I clinical study VRC 006 [13] was used in the construction of both the rAd5-EnvA and rAd35-EnvA adenoviral vector vaccines in this study. To create synthetic gp140 versions of the clade A envelope gene truncated at the transmembrane domain of gp41, the sequence from 92rw020 clade A strain (CCR5-tropic, GenBank accession number U08794) was used. The cleavage site and fusion peptide at the junction of envelope gp120 and gp41 regions were deleted and a portion of the interspace between the 2 heptad repeat regions in gp 41 was deleted as previously described [34].

2.1.2 VRC-HIVADV027-00-VP

VRC-HIVADV027-00-VP (rAd35-EnvA) has been constructed to express a truncated modified version of the HIV-1 clade A *env* gene. GenVec's proprietary manufacturing technology was used for HIV adenovector generation to reduce the risk of replication competent adenovirus (RCA) generation during clinical production. The rAd35-EnvA adenovector consists of the human adenovirus B, serotype 35 (Ad35), genome with a deletion of the E1 region. The E1 region deletion renders the adenovector replication deficient. The design of the HIV-1 EnvA gene insert has been previously described [13] as has the construction of similar rAd5 drug substances [35].

The 293-ORF6 cell line used to propagate the rAd35 vector was developed at GenVec, Inc. These cells were constructed by stable transfection of 293 cells (which are of human embryonic kidney origin) with an inducible expression cassette. This enables the cells to efficiently complement the E1-deleted rAd35 vector and greatly reduces the potential to generate replication-competent adenovirus. The generation of RCA would require recombination events; based on low homology, this is predicted to be extremely rare [36]. An assay for RCA is performed in the final release testing for all vectors. The 293-ORF6 Master Cell Bank (MCB) produced for GenVec by BioReliance (Gaithersburg, MD) was characterized with respect to safety, genetic and molecular characteristics and stability according to the requirements necessary to support current cGMP production, and these studies have satisfactorily established

the viability of the MCB and confirmed the authenticity of the cell line [37].

2.1.3 VRC-HIVADV038-00-VP

VRC-HIVADV038-00-VP (rAd5-EnvA) was manufactured in 293-ORF cells and tested for identity, potency, impurity and safety in a process similar to that previously described for the multiclade, multigene rAd5 vaccine product, VRC-HIVADV014-00-VP [35]. These tests included assays for adventitious pathogens, infectivity, sterility, and replication competent adenovirus type 5. The rAd5-EnvA was formulated and filled at 1.2mL into a sterile 3 mL glass vial and frozen.

2.2 PRECLINICAL SAFETY STUDIES

The Investigator Brochure provides more extensive information about the pre-clinical safety studies. Briefly described here are the biodistribution and toxicology studies conducted in rabbits with the rAd35-EnvA, VRC-HIVADV027-00-VP.

2.2.1 Good Laboratory Practices (GLP) Toxicity Study in New Zealand White Rabbits

Gene Logic (Gaithersburg, MD) conducted a GLP toxicology study no. 1542-05716, "Ad35 Vectored HIV Vaccine (VRC-HIVADV027-00-VP): 56-Day Intramuscular Toxicity Study in New Zealand White Rabbits". Briefly, groups of 10 animals/gender (11-16 week old New Zealand white rabbits) were inoculated IM with formulation buffer or with 1 x 10^{11} PU VRC-HIVADV027-00-VP in a split dose (0.5 mL injections 1 inch apart) on Study Days 1, 22, and 43 alternating from right thigh to left thigh and back with successive doses (consideration has been given to 2 inoculations in a clinical trial). One half of the animals (5/gender) were sacrificed on Study Day (SD) 45 and the remainder on SD57.

All animals survived to necropsy. No treatment-related observations were made with regard to clinical observations, body temperatures, Draize, ophthalmology, organ weights, or gross pathology. Treated animals did have slightly lower increases in body weights and concordant lower weight changes and food consumption than controls. With regard to food consumption, transient decreases in both genders were noted in the 24 hours following the 2nd adenovector delivery, in females in the 24 hours following the 3rd adenovector delivery, and in males in the 48 hours following the 3rd adenovector delivery, suggesting increasing reactogenicity with repeated dosing. Clinical pathology differences are described below. Injection site histopathology was observed at SD45 being slightly more severe and more frequent in treated animals vs. controls and demonstrating recovery by SD57 with only a minority of animals having evidence of ongoing reactogenicity approximately equivalent between treated animals and controls; but no other histopathological differences between groups were noted at SD45.

The following clinical pathology parameters appear to have been affected by treatment with Ad35 vector: elevated mean globulin values [male (♂) - SD4, 30, 45; female (♀) - SD30, 45, 57) remaining within the normal historical range, which may reflect the intended immune response to vaccination; decreased albumin to globulin (A/G) ratios reflective of the elevated globulin levels; decreased mean hemoglobin and hematocrit at SD30 and 45 (though hemoglobin was elevated in ♀ at SD57) remaining within the normal historical range; elevated mean corpuscular hemoglobin concentration in ♂ at SD57 exceeding the normal range; decreased platelets in ♀ at SD4 remaining within the normal historical range; prolonged activated partial thromboplastin time (aPTT) (♂ - SD4, 30; ♀ - SD4, 45) remaining within the normal historical

range; shortened prothrombin time (PT) (♀ - SD57) remaining within the normal historical range; elevated fibrinogen (both genders at SD4, 30, 45 and ♀ - SD57) falling outside the normal range at SD45; elevated absolute monocytes in ♀ at SD45 which may reflect the intended immune response to vaccination; and elevated creatinine kinase in both genders at SD45 (though decreased in ♀ at SD57), which may reflect muscle damage subsequent to repeated intramuscular immunization.

Many of these effects were also noted in studies of Ad5 vectors used alone or as a prime-boost, suggesting an impact of this product class (adenovectors) rather than the specific immunogen encoded. These effects suggest acute inflammation subsequent to adenovector delivery at high doses [10^{11} PU (or VP) or 2 x 10^{11} VP). While the effects in this study were of a magnitude that a statistical difference from 10 concurrent controls could be noted, most did not achieve a magnitude that exceeded the normal historical range from ~700 control gender-matched rabbits tested in the same laboratory. The prolonged aPTT values may suggest that there was an impact on the intrinsic coagulation pathway, but not on the extrinsic (normal *in vivo*) pathway. Alternatively, there is a report in the literature of a human adenovector gene therapy trial in which 6 of 11 recipients developed prolongation of aPTT, which was linked to the development of antiphospholipid antibodies and their impact on the test method, rather than an actual coagulation abnormality [38]. In a Phase I study of VRC-EBOADV018-00-VP, which is an adenovirus serotype5 Ebola vaccine (Ebola-rAd5), the VRC has observed that two subjects had abnormal aPTT laboratory values following injection. This was assessed as an *in vitro* phenomenon in the laboratory assay for aPTT that is due to a self-limited induction of an anti-phospholipid antibody (APA); there was no indication of a clinically significant *in vivo* safety issue. As part of the aPTT laboratory assay procedure, calcium and phospholipid are added and the time to clot formation is measured. Because phospholipid is a limiting ingredient in the assay, the presence of an APA can cause a prolonged aPTT. The assay for PT is much less affected by the presence of phospholipid and the thrombin time is not affected by the presence of phospholipid. This phenomenon is not associated with coagulation abnormalities *in vivo*, and as the antibody waned over 4-6 weeks the *in vitro* phenomenon resolved.

None of the clinical pathology parameters resulted in clinical symptomatology or histopathological changes. Lacking any correlative pathology, the clinical relevance of these findings is unclear. However, the inflammation that may be impacting these parameters may also contribute to the effects seen on food consumption and in the case of Ad5 vectors, fevers noted subsequent to vaccination (i.e., systemic reactogenicity).

Immunogenicity results demonstrated that all vaccinated animals seroconverted to the EnvA antigen. Endpoint titers were not determined, but testing was performed on sera at dilutions of 1:100, 1:1000, and 1:10,000. All vaccinated animals remained seropositive at the 1:10,000 dilution. No placebo-recipients seroconverted. These results demonstrate that active doses of vaccine were delivered to the test animals.

The toxicology profile of Ad35 appears to be better than Ad5 at comparable doses. With repeated dosing of Ad35, the profile begins to worsen slightly. However, it should be acknowledged that the tissue distribution of CD46 (or related molecule) in the rabbit is unknown, so it may be that the rabbit is not as good a toxicology model for CD46-utilizing adenovectors as it has proven to be for Ad5. Given that many of the same parameters that are impacted by Ad5 delivery were also noted in this study, however, the rabbit would seem to be an appropriate model for CD46-utilizing adenovectors. Human data from on-going Ad35 vector clinical trials

will help to verify this model.

2.2.2 Pyrogenicity Study in Rabbits

Charles River Laboratory (Horsham, PA) conducted a non-GLP study no. COG00010, "Four-Day Single Intramuscular Pyrogenicity Study of HIV Adenoviral Constructs in Male New Zealand White Rabbits". Briefly, groups of 5 male animals/vector (11-16 week old New Zealand white rabbits) were inoculated IM with a 1 x 10^{12} PU dose of either Ad5 or Ad35 vectors expressing a luciferase gene (delivered as 4 x 0.5 mL, split between left and right thigh muscles) on SD1. This dose is one to two logs higher than the human clinical doses and one log higher than the dose of the clinical product in the GLP repeated dose toxicology study. Control animals (n=3) received a total of 1 mL of formulation buffer. Temperatures were assessed prior to vaccination, three hours post immunization (p.i.) and then daily for 4 days.

All groups of adenovector–vaccinated rabbits showed elevated mean temperatures (>1° C) in the first 24 hours, with most resolving in 48 hours. Temperature data plotted over the 4-day study showed that there were no temperatures recorded >41° C (normal temperature of a rabbit is ~38.5° C). Temperatures at 24 hours p.i. showed that the Ad5-vaccinated animals showed an average temperature of 40.2° C, which was elevated significantly compared to the average (38.8° C) observed in the control animals (p = 0.01); the average 24 hour p.i. temperature observed in the Ad35-vaccinated animals was 39.8° C and did not differ significantly from control animals (p = 0.88).

2.2.3 GLP Biodistribution Study of VRC-HIVADV027-00-VP in Rabbits

GLP Study no. 1542-05714, "Ad35 Vectored HIV Vaccine (VRC-HIVADV027-00-VP): 90-Day, Single Intramuscular Dose Biodistribution Study in New Zealand White Rabbits" was conducted by Gene Logic Inc. (Gaithersburg, MD) to evaluate the distribution of the rAd35-EnvA vaccine. Briefly, 10 animals/gender (15-16 week old New Zealand white rabbits) were inoculated IM into the right thigh with 0.5 x 10^{11} PU of VRC-HIVADV027-00-VP on SD1. Ten animals/gender were inoculated with formulation buffer also on SD1. One half of the animals (5/gender) were sacrificed on SD9 and SD91. Tissues were collected and sent to Althea Technologies, Inc (San Diego, CA) for polymerase chain reaction (PCR) quantification.

All animals survived to necropsy. No treatment-related observations were made with regard to clinical observations and body weight changes. The adenoviral type 35 vector given IM biodistributed within the injection site sub-cutis and muscle and to the iliac lymph nodes and spleens in vaccinated animals. Over the time course from 9 days post-inoculation to 91 days post-inoculation, the number of animals with positive tissues, the number of tissues with positive signals, and amount of detectable vector in those tissues clears. By SD91, only the injection site muscle and the right iliac lymph nodes continue to have very low level positive signals (averages of 98 and 34 copies/microgram tissue DNA, respectively).

2.3 SUMMARY OF PRECLINICAL IMMUNOGENICITY STUDIES

The Investigator Brochure provides more extensive information about the preclinical immunogenicity studies. There is no adequate animal model of HIV-1 infection and, as a consequence, there can be no animal studies in which to test pharmacology and protective immunization with the adenoviral vector vaccines. Non-clinical, non-GLP immunogenicity studies were conducted with Ad35 adenovectors in mice by investigators at the VRC, NIAID,

NIH (Bethesda, MD). A flow cytometry-based ICS assay was used to evaluate the cellular immune responses elicited by the vaccine, measuring cytokines produced by antigen stimulated cells. The stimulated cells are further characterized by phenotypic lymphocyte markers, allowing for precise quantification of CD4+ or CD8+ T-lymphocytes responding to the vaccine antigens by methods previously described [26]. Materials evaluated in the immunogenicity studies consisted of recombinant E1/E3/E4-deleted Ad5 or E1-deleted Ad35-based adenovectors (some with modified fiber proteins), made in 293-ORF6 cells. Recombinant vectors expressed either a HIVgp140 clade A envelope (EnvA) gene insert or a green fluorescence protein gene insert.

2.3.1 Immune Responses to Viral Gene Products in Mice

Non-clinical, non-GLP immunogenicity studies were conducted with rAd35 adenovectors in mice by investigators at the VRC, NIAID, NIH (Bethesda, MD) in order to compare the immunogenicity of recombinant adenovectors based upon wild-type *human adenovirus C serotype 5* (rAd5) and *human adenovirus B serotype 35* (rAd35) expressing EnvA.

HIV EnvA-specific CD4+ and CD8+ cellular immune responses were demonstrated by ICS in all mice vaccinated with both rAd5- and rAd35-based adenovectors expressing EnvA. rAd35-based vectors trended to be less immunogenic than rAd5 vectors in Ad5-naïve mice. Sera from the vaccinated mice also demonstrated anti-HIV EnvA IgG titers in ELISA assays. Mice pre-exposed to Ad5 antibody did not show a reduction in immunogenicity of rAd35 vectors, however this Ad5-pre-exposure did reduce rAd5 vector immunogenicity in mice. These data suggested that rAd35 vectors are immunogenic, and that such vectors would retain their immunogenic potential in the setting of pre-existing Ad5 immunity and therefore deserved further evaluation in non-human primate models.

2.3.2 Immunogenicity of Ad35-based Recombinant Adenoviral Vector Vaccines in Rhesus Monkeys

Preclinical studies conducted with rAd5, rAd35 and chimeric rAd35 vectors are summarized in the IB. The immunogenicity of adenovectors constructed with Ad5 and Ad35 components are relevant to VRC 012. Briefly, groups of Rhesus macaques (6/group) were first vaccinated with either 1 x 10^{10} PU replication–deficient rAd5 or rAd35 expressing EnvA. Animals were then boosted at Week 12, with a heterologous adenovector. One group was primed with rAd5-EnvA, followed by rAd35-EnvA boost and a second group was primed with rAd35-EnvA, followed by rAd5-EnvA boost.

ELISpot and ICS were utilized to monitor the emergence of vaccine-elicited T cell immune responses to EnvA antigen by previously described methods [39], and sera were assayed for anti-EnvA antibody by ELISA, using plates coated with HIV envelope A protein. Two weeks following immunization, EnvA specific-T-cell immunogenicity was demonstrated by ICS and ELISpot for both rAd5-EnvA and Ad35-EnvA vectors. The strength of effector T-cell response, as measured by ELISpot assay in monkeys, was greater for rAd5 than for rAd35 at 2, 4 and 8 weeks after initial priming. After boosting, the highest overall T-cell responses (~1500 spot forming cell/1x10^6 cells) were observed in rAd35-EnvA immunized monkeys, boosted with rAd5-EnvA.

Pre-boost ELISA titers (3 and 6 wks post-injection) were also greater for rAd5-EnvA primed monkeys than for those immunized with rAd35-EnvA. Heterologous boosting with either rAd5-EnvA or rAd35-EnvA resulted in increased antibody responses, but the rAd35-EnvA priming

with rAd5-EnvA boosting was greatest as it was for the T-cell responses.

These studies demonstrate that heterologous rAd priming and boosting combination regimens can induce augmented HIV-specific immune responses in monkeys compared to a single rAd injection regimen.

3. STUDY OBJECTIVES

3.1 PRIMARY OBJECTIVES

- To evaluate the safety and tolerability of the rAd35-EnvA vaccine (VRC-HIVADV027-00-VP) when administered IM via needle and syringe at a dosage of 10^9 PU.
- To evaluate the safety and tolerability of the rAd35-EnvA vaccine when administered IM via needle and syringe at a dosage of 10^{10} PU.
- To evaluate the safety and tolerability of the rAd35-EnvA vaccine when administered IM via needle and syringe at a dosage of 10^{11} PU.
- To evaluate the safety and tolerability of the rAd5-EnvA vaccine (VRC-HIVADV038-00-VP) when administered IM via needle and syringe at a dosage of 10^{10} PU in heterologous prime-boost regimens with the rAd35-EnvA vaccine at dosages of 10^{10} PU and 10^{11} PU.

3.2 SECONDARY OBJECTIVES

- To evaluate the magnitude and frequency of immune response to the rAd35-EnvA vaccine at dosages of 10^{10} and 10^{11} PU and rAd5-EnvA vaccine at a dosage of 10^{10} PU administered IM via needle and syringe as indicated by intracellular cytokine staining, ELISpot, vaccine antigen-specific ELISA, neutralization assays and other immunological assays at Study Week 4.
- To evaluate the magnitude and frequency of immune response to the four heterologous prime-boost regimens as indicated by intracellular cytokine staining, ELISpot, vaccine antigen-specific ELISA, neutralization assays and other immunological assays at Study Week 16.
- To evaluate Ad5 and Ad35 neutralizing antibody titers at 4 weeks after the first injection administered and 4 weeks after the boost injection.
- To monitor the social impact of participating in an HIV-1 vaccine clinical trial.

3.3 EXPLORATORY OBJECTIVES

- To compare the immune response of the rAd35-EnvA vaccine at dosages of 10^{10} and 10^{11} PU to the immune response to the rAd5-EnvA (VRC-HIVADV038-00-VP) vaccine at a dosage of 10^{10} PU by evaluating the magnitude of antigen-specific T cell responses to EnvA by ELISpot at 4 weeks after completing the priming vaccinations (*i.e.*, at Study Week 4).
- To compare the immune response of the heterologous prime-boost regimens by evaluating the magnitude of antigen-specific T cell responses to EnvA by ELISpot at 4 weeks after completing the prime-boost vaccination schedule (*i.e.,* at Study Week 16).

- To evaluate the magnitude and frequency of immune response to the rAd35-EnvA vaccine at dosages of 10^{10} and 10^{11} PU administered IM via needle and syringe and to evaluate the magnitude and frequency of immune response to the four prime-boost regimens as indicated by intracellular cytokine staining, ELISpot, vaccine antigen-specific ELISA, neutralization assays and other immunological assays at various timepoints throughout the study.
- To evaluate the long-term immunogenicity of the prime-boost regimen in subjects at Study Week 36 and 52.
- To evaluate humoral and T-cell immune responses to the adenoviral vectors by a variety of exploratory immunological assays.

4. STUDY DESIGN

This is a two-part Phase I study. Part I is designed to first evaluate the safety and tolerability of the rAd35-EnvA vaccine alone in an open-label, dose-escalation design in 15 HIV-uninfected subjects (18-50 years old). Group 1 (N=5) will receive one intramuscular injection of rAd35-EnvA at a dose of 10^9 PU. Group 2 (N=5) will receive one injection at a dose of 10^{10} PU, and Group 3 (N=5) will receive one injection at a dose of 10^{11} PU. In all groups vaccine administration will be on Day 0. Each dose group will enroll no more than one subject per day. Criteria for dose escalation are described in Section 4.6.

Part II is designed as a randomized, double-blind evaluation of the safety and tolerability of heterologous prime-boost adenoviral vector regimens. Twenty HIV-uninfected subjects (18-50 years old), will be randomized to four vaccination schedules as follows:

- Group 4A will receive 10^{10} PU rAd35-EnvA prime with 10^{10} PU rAd5-EnvA boost (N=5).
- Group 4B will receive 10^{10} PU rAd5-EnvA prime with 10^{10} PU rAd35-EnvA boost (N=5).
- Group 4C will receive 10^{11} PU rAd35-EnvA prime with 10^{10} PU rAd5-EnvA boost (N=5).
- Group 4D will receive 10^{10} PU rAd5-EnvA prime with 10^{11} PU rAd35-EnvA boost (N=5).

The first 10 subjects in Group 4 will be randomized in a 1:1 ratio into Groups 4A and 4B. The last 10 subjects in Group 4 will be randomized in a 1:1 ratio into Groups 4C and 4D. All rAd5-EnvA and rAd35-EnvA injections will be administered IM.

Both Part I and Part II will evaluate immunogenicity of the study vaccines from frozen samples. The immunogenicity evaluations are not factors in the initiation of sequential study group enrollments. The schemas for Part I and Part II are shown in the tables that follow:

Table 4.1. VRC 012 PART I: Dose Escalation

**Open-label; Sequentially Enrolled Groups**	**N =**	**Day 0 VACCINATION**
Group 1	5	**rAd35-EnvA** (10^9 PU IM)
Group 2	5	**rAd35-EnvA** (10^{10} PU IM)
Group 3	5	**rAd35-EnvA** (10^{11} PU IM)
**TOTAL**	**15**	

Table 4.2. VRC 012 PART II: Heterologous Prime-Boost Schedules

Randomized, Double-Blinded Enrollment	N =	Day 0 PRIME	Week 12 (-7 or +21 days) BOOST
Group 4A	5	**rAd35-EnvA** (10^{10} PU IM)	**rAd5-EnvA** (10^{10} PU IM)
Group 4B	5	**rAd5-EnvA** (10^{10} PU IM)	**rAd35-EnvA** (10^{10} PU IM)
Group 4C	5	**rAd35-Env A** (10^{11} PU IM)	**rAd5-EnvA** (10^{10} PU IM)
Group 4D	5	**rAd5-EnvA** (10^{10} PU IM)	**rAd35-EnvA** (10^{11} PU IM)
**TOTAL**	**20**		

The primary hypotheses are that rAd35-EnvA vaccine will be safe for human administration at dosages up to 10^{11} PU as a single agent and both the rAd35-EnvA and rAd5-EnvA vaccines will be safe in prime-boost regimens. The secondary hypotheses include that both the rAd35-EnvA and rAd5-EnvA vaccines will elicit an immune response to the EnvA immunogen; and the exploratory hypotheses include that the heterologous prime-boost regimens will elicit a greater frequency and magnitude of response than after priming vaccinations alone. Safety of the vaccine regimens will be evaluated at scheduled study visits and by study subject report.

Specimens to evaluate immunogenicity will be taken at baseline and at specified time points. The HIV-1-specific immune responses will be assessed by cellular immune function assays and humoral immunity assays. Part I study subjects will require 24 weeks of follow-up and Part II subjects will require 52 weeks of follow-up. In addition, Part II subjects will be contacted annually for 4 more years after last study visit for collection of long-term follow-up information.

4.1 STUDY POPULATION

All study activities will be carried out at the National Institutes of Health. Thirty-five healthy, HIV-negative volunteers will be recruited and screened through VRC 000 (02-I-0127), a screening protocol for healthy volunteers who are interested in participating in HIV vaccine clinical trials, to confirm eligibility requirements for participation. The screening and education process required prior to enrollment should ensure that subjects comprehend the purpose and details of the study. This Phase I study will be limited to adults who are 18-50 years old at the time of enrollment.

Prior to signing the VRC 012 informed consent, eligible volunteers will take a short “Assessment of Understanding” quiz to test understanding of this vaccine study. Incorrect answers will be explained to the volunteer and they will sign the informed consent document only after the study coordinator is satisfied with their understanding of the study.

4.1.1 Inclusion Criteria

***A participant must meet all of the following criteria*:**

1. 18 to 50 years old.

2. Available for clinical follow-up through Week 24 of the study for subjects in Part I; available for clinical follow-up through Week 52 for subjects in Part II and committed to 4 years of annual follow-up contact after Week 52 if in Part II of the study.

3. Able to provide proof of identity to the satisfaction of the study clinician completing the enrollment process.

4. Complete an Assessment of Understanding (that includes understanding of the Step Study results) prior to enrollment and verbalize understanding of all questions answered incorrectly.

5. Able and willing to complete the informed consent process.

6. Willing to receive HIV test results and willing to abide by NIH guidelines for partner notification of positive HIV results.

7. Willing to donate blood for sample storage to be used for future research.

8. Willing to discuss HIV infection risks, amenable to risk reduction counseling, committed to maintaining behavior consistent with low risk of HIV exposure through the last required protocol clinic visit and assessed by the clinic staff as being at low risk of HIV infection on the basis of behaviors in the 12 months prior to enrollment as follows:

Sexually abstinent<br><br>**OR**<br><br>Had two or fewer mutually monogamous relationships with partners believed to be HIV-uninfected and who did not use injection drugs, crack cocaine or methamphetamine<br><br>**OR**<br><br>Had three or fewer partners believed to be HIV-uninfected and who did not use injection drugs, crack cocaine or methamphetamine and with whom he/she regularly used condoms for vaginal or anal intercourse

9. In good general health without clinically significant medical history.

10. Physical examination and laboratory results without clinically significant findings within the 56 days prior to enrollment.

Laboratory Criteria within 56 days prior to enrollment:

11. Hemoglobin ≥ 11.5 g/dL for women; ≥13.5 g/dL for men.

12. White blood cells (WBC) = 3,300-12,000 cells/mm^3.

13. Differential either within institutional normal range or accompanied by site physician approval.

14. Total lymphocyte count ≥ 800 cells/mm^3.

15. Platelets = 125,000 – 550,000/mm^3.

16. Alanine aminotransferase (ALT) ≤ 1.25 x upper limit of normal.

17. Serum creatinine ≤ upper limit of normal (i.e., ≤1.3 mg/dL for females; ≤1.4 mg/dL for males).

18. Normal urinalysis defined as negative glucose, negative or trace protein, and no clinically significant blood in the urine.

19. Negative FDA-approved HIV blood test.

20. Negative hepatitis B surface antigen (HBsAg).

21. Negative anti-hepatitis C virus (HCV) antibody and negative HCV PCR.

Laboratory Criteria within 84 days prior to enrollment:

22. Seronegative for Ad35 antibody if enrolled in Part I after the Version 2.0 amendment and seronegative for both Ad5 and Ad35 antibody if in Part II.

Female-Specific Criteria:

23. Negative β-human chorionic gonadotropin pregnancy test (urine or serum) on day of enrollment for women presumed to be of reproductive potential.

24. A female participant must meet any of the following criteria:

    No reproductive potential because of menopause [one year without menses] or because of a hysterectomy, bilateral oophorectomy, or tubal ligation,

    or

    Participant agrees to be heterosexually inactive at least 21 days prior to enrollment and through Week 12 of the study for subjects in Part I and through Week 24 of the study for subjects in Part II,

    or

Participant agrees to consistently practice contraception at least 21 days prior to enrollment and through Week 12 of the study for subjects in Part I or through Week 24 of the study for subjects in Part II by one of the following methods:

- condoms, male or female, with or without a spermicide
- diaphragm or cervical cap with spermicide
- intrauterine device
- contraceptive pills or patch, Depo-Provera or other FDA-approved contraceptive method
- male partner has previously undergone a vasectomy.

4.1.2 Exclusion Criteria

A volunteer will be excluded if one or more of the following conditions apply:

Women:

1. Woman who is breast-feeding or planning to become pregnant during the 12 weeks of study participation for subjects in Part I and 24 weeks of study participation for subjects in Part II.

Volunteer has received any of the following substances:

2. HIV vaccine in a prior clinical trial.
3. Immunosuppressive medications, cytotoxic medications, inhaled corticosteroids, or long-acting beta-agonists within the past three months. [With the exceptions that use of corticosteroid nasal spray for rhinitis; topical corticosteroids for an acute uncomplicated dermatitis; short-acting beta-agonists in controlled asthmatics; or a short course (10 days or less) of corticosteroids for a non-chronic condition at least 2 weeks prior to enrollment in this study will not exclude study participation.]
4. Blood products within 120 days prior to HIV screening.
5. Immunoglobulin within 60 days prior to HIV screening.
6. Investigational research agents within 30 days prior to initial study vaccine administration.
7. Live attenuated vaccines within 30 days prior to initial study vaccine administration.
8. Medically indicated subunit or killed vaccines, e.g. influenza, pneumococcal, or allergy treatment with antigen injections, within 14 days of study vaccine administration.
9. Current anti-tuberculosis prophylaxis or therapy.

Volunteer has a history of any of the following clinically significant conditions:

10. Serious adverse reactions to vaccines such as anaphylaxis, urticaria (hives), respiratory difficulty, angioedema, or abdominal pain.
11. Idiopathic urticaria within the past 2 years.

12. Autoimmune disease or immunodeficiency.

13. Asthma that is unstable or required emergent care, urgent care, hospitalization or intubation during the past two years or that requires the use of oral or intravenous corticosteroids.

14. Diabetes mellitus (type I or II), with the exception of gestational diabetes.

15. History of thyroidectomy or thyroid disease that required medication within the past 12 months.

16. A history of hereditary angioedema, acquired angioedema, or idiopathic forms of angioedema.

17. Hypertension that is not well controlled by medication or blood pressure that is more than 145/95 at enrollment.

18. Bleeding disorder diagnosed by a doctor (e.g., factor deficiency, coagulopathy, or platelet disorder requiring special precautions) or significant bruising or bleeding difficulties with IM injections or blood draws.

19. Within the 12 months prior to enrollment: newly-acquired syphilis, gonorrhea, non-gonococcal urethritis, herpes simplex virus type 2 (HSV2), chlamydia, pelvic inflammatory disease (PID), trichomonas, mucopurulent cervicitis, epididymitis, proctitis, lymphogranuloma venereum, chancroid, or hepatitis B.

20. Malignancy that is active or treated malignancy for which there is not *reasonable* assurance of sustained cure or malignancy that is likely to recur during the period of the study.

21. Seizure disorder other than: 1) febrile seizures under the age of two, 2) seizures secondary to alcohol withdrawal more than 3 years ago, or 3) a singular seizure not requiring treatment within the last 3 years.

22. Asplenia, functional asplenia or any condition resulting in the absence or removal of the spleen.

23. Psychiatric condition that precludes compliance with the protocol; past or present psychoses; past or present bipolar disorder; disorder requiring lithium; or within five years prior to enrollment, history of a suicide plan or attempt.

24. Any medical, psychiatric, social condition, occupational reason or other responsibility that, in the judgment of the investigator, is a contraindication to protocol participation or impairs a volunteer’s ability to give informed consent.

25. BMI ≥ 40; <u>OR</u> BMI ≥ 35 AND with one or more of the following:
    - Age > 45
    - Systolic blood pressure > 140 mm Hg

- Diastolic blood pressure > 90 mm Hg
- Current smoker or quit smoking within 28 days of enrollment
- Untreated or poorly controlled hyperlipidemia

26. Within the 12 months prior to enrollment, one or more of the following:
    - excessive daily alcohol use
    - frequent binge drinking
    - chronic marijuana abuse
    - any other illicit drug use.

4.2 Schedule of Clinical Procedures and Laboratory Evaluations

Evaluation of the safety of this vaccine will include laboratory studies, medical history, physical assessment by clinicians, and subject self-assessment recorded on a diary card. Potential adverse reactions will be further evaluated prior to continuing the immunization schedule. Blood tests for immune responses will be performed at the Vaccine Research Center. The study schedule is described in Section 4.2.2 and presented in the form of a Table in Appendix III. Total blood volume drawn from each subject will not exceed the NIH Clinical Center Guidelines of 450 mL in any 6-week period.

4.2.1 Screening

Screening for this study will be completed through the Vaccine Research Center's Screening Protocol, VRC 000 (NIH 02-I-0127). The evaluations and sample collection that will be included in VRC 000 screening are a medical history, physical exam, complete blood count with differential, PT, aPTT, chemistry panel, quantitative immunoglobulins, rapid plasma reagin (RPR), hepatitis B surface antigen, anti-hepatitis B antibody, anti-hepatitis C antibody, HCV PCR, anti-dsDNA, HIV ELISA/Western Blot, HIV PCR, urinalysis, Ad5 and Ad35 antibody tests, pregnancy test (for females of reproductive potential), and questions regarding sexual behavior and other practices. Any test that has a specific eligibility requirement must be done within the window needed to meet study eligibility. Risk status for HIV infection will be determined by a series of questions designed to identify risk factors. Storage samples of PBMCs and serum will also be collected. General eligibility for clinical trials will be dependent on results of laboratory tests and answers to the interview questions. Informed consent documents for vaccine trials will be reviewed, and counseling relating to the potential risks of becoming pregnant during this trial and avoiding HIV infection will be provided. An Assessment of Understanding of VRC 012 is completed on the day the subject is scheduled to enroll in VRC 012.

4.2.2 Day 0 through End of Clinical Follow-up

Day 0 is defined as the day of VRC 012 enrollment and first injection. Study-specific eligibility is reviewed on Day 0 as part of the enrollment process. Pregnancy test results for women of childbearing potential must be confirmed as negative prior to enrollment on Day 0 and prior to each vaccination for Part II female participants. Subjects who enroll in Group 4 will begin their randomized prime-boost schedule on the day of enrollment with the first vaccination. Day 0 evaluations prior to the first vaccination are the baseline for subsequent safety assessments.

Vaccinations for Groups 1, 2 and 3 are open-label. All vaccinations for Group 4 will be blinded with regard to sequence of study vaccine administration. The Protocol Statistician will prepare the randomization plan and provide it to the Site Pharmacy in advance so that the pharmacy database can be set up prior to opening the study to accrual. For Group 4 participants, both clinic staff and subjects will know that the first ten Group 4 subjects are in subgroups 4A and 4B, while the last ten Group 4 subjects are in subgroups 4C and 4D. In this way they will know the dosage of the two study vaccines in the respective schedules but will not know which vaccine type is being administered as the prime or the boost. Enrollment on Day 0 is followed on the same day by the first study injection.

Vaccination Schedule for Groups 1, 2, and 3:

Day 0 vaccination will be administered intramuscularly in the deltoid with a needle and syringe.

Following the study injection subjects will be observed for a minimum of 30 minutes. Vital signs (temperature, blood pressure, pulse and respiratory rate) will be completed between 30 and 45 minutes post-immunization and the injection site will be inspected for evidence of local reaction. Subjects will be given a Diary Card to use as a memory aid in recording temperature and symptoms daily for 5 days. Diary cards may be completed on paper or electronically in a password-protected system and will be reviewed with the clinician at any visits that occur from Day 0 through the first study visit following completion of Day 5.

Vaccination Schedule for Group 4:

Enrollment in Group 4 is contingent upon the interim Data and Safety Monitoring Board (DSMB) review (see Section 4.6) and will not proceed until Group 3 is fully enrolled. Once the randomized enrollments in Groups 4A and 4B are done, the randomized enrollments into Groups 4C and 4D will proceed.

The prime vaccination will occur on Day 0 and the booster vaccination will occur as close to Day 84 as possible (with a -7 days to +21 days window permitted for scheduling). All vaccinations will be administered intramuscularly in the deltoid with a needle and syringe.

Observations in the Clinic on the Day of a Vaccination:

Following each study injection, subjects will be observed for a minimum of 30 minutes. Vital signs (temperature, blood pressure, pulse and respiratory rate) will be completed between 30 and 45 minutes post-immunization and the injection site will be inspected for evidence of local reaction.

5-Day Solicited Reactogenicity:

Subjects will be given a “Diary Card” with instructions for recording temperature measurements and reactogenicity symptoms daily for 5 days. Diary cards may be completed either on paper or recorded directly by the subject electronically in a password-protected system. Solicited reactogenicity will be reviewed with the clinician at any visits from Day 0 through the first study visit following completion of Day 5. Either the subject’s electronic or written diary card may be used as a source document. When neither a written nor electronic diary is available, the study clinician will note the source of the reactogenicity information.

Follow-up Schedule:

The first follow-up for any vaccination will be performed by telephone on Day 2 ±1 day

following the injection. A clinic visit will occur within 24 hours if indicated by the telephone interview. Events reported in the telephone interview that will require a clinic visit include rash, urticaria (hives), fever of 38.7˚C (Grade 2) or higher that does not resolve within 24 hours, or significant impairment in the activities of daily living (ADL). At 14 ±3 days after any vaccination, study subjects will be evaluated at a clinic visit. This visit will include interim history, vital signs, lymph node exam and examination of the vaccination site. The 5-day Diary Card will be reviewed at this time.

The schedule of follow-up visits, permitted windows for completing the visits and evaluations performed at each visit is shown in the table in Appendix III.

At intervals throughout the study subjects will have blood drawn for immunologic assays. Any cells, serum or plasma not used will be stored for future virological and immunological assays. Study procedures and tests included in this protocol are listed below; refer to Appendix III for detailed requirements regarding at which visits these are performed. After Day 0, deviations from the visit windows noted in Appendix III are discouraged and will be recorded as protocol deviations, but are permitted, at the discretion of the PI (or designee) in the interest of obtaining subject safety and immunogenicity evaluations following exposure to the investigational vaccine.

- "VRC 012 Assessment of Understanding" Quiz
- Signature of study participation informed consent form for VRC 012
- Clinical evaluations: vital signs and weight, axillary lymph node exam and targeted physical exam when indicated by interim complaints or laboratory findings.
- Interim medical history.
- Counseling on HIV and avoidance of pregnancy.
- Study vaccinations; schedule by group assignment.
- Post-injection vital signs and assessment of injection site at 30 to 45 minutes after a study vaccination.
- Diary Card: Baseline on day of vaccination; 5-day diary card for self-assessment by subject following each vaccination. The diary card will include the parameters: unusually tired/feeling unwell, muscles aches (at other than injection site), headache, chills, nausea, and pain/tenderness at injection site. Subjects will also record highest measured temperature, measurement of perpendicular diameters for redness and swelling at injection site and note if there is evidence of a skin lesion at the vaccination site. To ensure prompt assessment, completed diary cards will be collected by a secure web based data entry system, pre-paid clinic provided FedEx envelope, or at a clinic visit.
- Serum or urine pregnancy test, for females of reproductive potential. Negative pregnancy test results must be confirmed prior to vaccination. If a subject becomes pregnant after enrollment, the outcome of the pregnancy will be sought and recorded.
- HLA (human leukocyte antigen) type: blood sample shown as collected at Week 2 for convenience, but may be done from a sample collected at any timepoint, if blood draw allowance is not exceeded (or may be recorded from prior test results at the NIH Clinical Center).

- CBC, differential, platelet count.
- PT/aPTT
- Creatinine and ALT.
- Urinalysis.
- T cell FACS (fluorescence-activated cell sorter) for CD4/CD8; after day of enrollment this is done only in the event of a detectable HIV PCR.
- HIV testing: ELISA (also Western blot if ELISA is positive) and HIV PCR.
- HIV-specific antibody research assays. Note: The assays will not be performed immediately, but rather completed at a later date using frozen samples. May be performed at additional timepoints, using stored sera, if of interest.
- ELISpot and ICS assays. Note: The assays will not be performed immediately, but rather completed at a later date using frozen samples. PBMC and plasma for storage will be saved from the blood collected for these assays. Other immunological assays, such as multiparameter flow cytometry, may also be performed from stored samples.
- Social Impact Questionnaire: The Social Impact questionnaire will include parameters: personal relationships, travel or immigration, employment, education, medical or dental, health insurance, life insurance, housing, military/other government agency and other.
- Serum for archiving.
- Adenovirus serology; testing at any timepoint with stored serum may be performed.

4.2.3 Long-term Follow-up Contacts for Part II Subjects:

Part II subjects will be contacted annually for 4 years after the Week 52 clinic visit. Subjects will be encouraged to return for a clinic visit to be interviewed about:

- interval life-threatening adverse events,
- persistent or significant disability/incapacity,
- non-elective hospitalizations,
- new chronic diseases requiring ongoing medical management or medication,
- outcomes of any pregnancies (including if there were any congenital anomalies/birth defects)

HIV testing (ELISA with Western blot if positive and HIV PCR) and a research immunology blood draw (PBMC, plasma and serum) may also be performed. A subject may opt to be contacted by remote means (e.g., telephone, mail, e-mail) to allow collection of the interview information specified above without a follow-up blood draw. If there are any subject deaths, an attempt will be made to obtain information about cause of death. Subjects may also be contacted at other times to confirm contact information, to provide notification about release of study results and to inform subjects of schedule assignment in accordance with the study unblinding process.

4.3 Monitoring for HIV Infections

It is possible that this vaccination regimen will induce immunologic responses that are detected by standard HIV screening techniques, even though the vaccines will not cause HIV infection. The following steps will be taken to ensure detection of HIV infection and to protect participants from adverse consequences associated with an HIV antibody test that indicates an antibody response to the vaccine:

- Study participants will receive regularly scheduled counseling regarding avoidance of HIV infection in accordance with the most recent CDC HIV Counseling Guidelines.

- Study participants will be screened for HIV infection periodically while participating in the study (see Appendix III for schedule of testing).

- If there is any clinical or laboratory indication of HIV infection, any test required to make a definitive diagnosis, including Western blot analysis, viral load measurement (PCR), or other tests will be performed.

- Confirming tests will be performed as soon as possible once a positive antibody response is identified. Participants will be promptly informed if they are HIV-infected. Participants who are found to have vaccine-induced antibody responses, but with no evidence of HIV infection, will be informed that they are not HIV-infected. Written documentation describing any vaccine-induced antibody response and confirming data will be provided when the study is completed. This should be sufficient evidence that the antibody response as of the date of testing resulted from vaccination and not from naturally occurring infection. Participants with vaccine-induced antibody will be provided with the opportunity for HIV antibody testing annually for five years after enrollment or until study termination to monitor their serological status. Beyond five years after enrollment or after study termination, testing may be performed through a VRC sample collection protocol. VRC will also provide assistance in advising other medical professionals on how to complete a diagnostic algorithm that takes into account the characteristics of the vaccine to distinguish vaccine-induced antibody from HIV infection. Participants will be counseled regarding the potential for antibody responses and the implications of such responses prior to participation in the study.

4.4 Intercurrent HIV Infection

The vaccines cannot cause HIV infection. Subjects who become HIV infected while participating in the study will be referred for their medical care and treatment and management of the disease. They may be given the opportunity to enroll in an appropriate study of acute HIV infection or a long-term follow-up study, if one is available. The NIH investigators will not be responsible for providing ongoing medical care or antiretroviral medications in the event of HIV infection.

4.5 Concomitant Medications

Concomitant medications are recorded at screening and every study visit. If an FDA-approved live attenuated vaccine is required during the study vaccination schedule for an immediate medical need, then study injections must be discontinued if it cannot be administered with at least 14 days after the previous study vaccination and 30 days before the next study vaccination.

If an FDA-approved subunit or killed vaccine is required for an immediate medical need, then it must be given at least 14 days before or 14 days after any study injection for the subject to remain eligible for additional study injections. If it will not imperil a subject's health, FDA-approved vaccines should be deferred until at least 30 days after the final study injection. Any subject who receives at least one study injection will continue with the clinical and laboratory evaluations specified by the study.

4.6 CRITERIA FOR DOSE ESCALATION IN PART I AND INITIATION OF PART II

4.6.1 Part I Dose Escalation

Part I involves enrollment into 3 open-label, sequential dosage groups. No more than one subject per day will be enrolled into each Part I dose group. Dose escalation and enrollment in Groups 2 and 3 will be contingent upon a safety review by at least five members of the protocol safety review team (PSRT), which will include the Principal Investigator, the DAIDS Medical Officer and 3 or more other protocol safety review members (as noted in Section 8.9). The review will occur no earlier than 5 days after the last vaccination in each dose group and must confirm that no study pause rules are in effect and that no serious safety concerns requiring further evaluation are in progress.

4.6.2 Initiation of Part II (Group 4)

In order to begin enrollments into Part II of the study, an interim safety data review of the 10^9 PU and 10^{10} PU dose groups will be completed by the Intramural NIAID DSMB. The DSMB review of the 10^9 PU and 10^{10} PU safety data will occur no earlier than three weeks after the last Group 2 enrollment (i.e., when at least 2 weeks of follow-up on the last 10^{10} PU injection in Group 2 is available in the safety reports). The DSMB review may be concurrent with enrollment of Group 3 (i.e., 10^{11} PU dose group). However, initiation of Group 4, in which only 10^{10} PU injections will be administered to the first 10 subjects enrolled, will not begin until the safety review by the PSRT of Group 3 is completed. This will occur about 3 weeks after the last Group 3 enrollment (i.e., when 2 weeks of follow-up on the last 10^{11} PU injection is available in the safety reports). There will not be a pause between the first 10 subjects and last 10 subjects enrolled into Group 4 as PSRT safety reviews are weekly throughout the period of study vaccinations and the overall study pause rules (see Section 4.8) will apply if any safety concerns arise.

4.6.3 DSMB Reviews

The NIAID Intramural DSMB will review the protocol and related documents at the following times: 1) prior to study initiation; 2) to consider initiation of Part II through an interim safety review (to be scheduled as noted in Section 4.6.2); and 3) at the regularly scheduled DSMB meetings (unless the DSMB deems that a review is not needed). During the course of the study, the DSMB will review cumulative study data to evaluate safety, study conduct, and scientific validity and integrity of the trial. As part of this responsibility, DSMB members must be satisfied that the timeliness, completeness, and accuracy of the data submitted to them for review are sufficient for evaluation of the safety and welfare of study subjects. The DSMB may also convene as needed if stopping criteria are met or other safety issues arise that the Principal Investigator and/or NIAID Clinical Director or designee would like the DSMB to address. The DSMB recommendations will be submitted to the IRB as they become available. The Protocol

Statistician will provide the DSMB Executive Secretary with the randomization codes in a sealed envelope in the event that the DSMB will require this information to make its recommendations.

4.7 Criteria for Withdrawal of a Subject From the Injection Schedule

Part I subjects receive only one study injection. Under certain circumstances, a subject enrolled in Part II, will be terminated from participating in the booster injection. Participants who are discontinued from the vaccination schedule will continue to be followed according to the schedule of safety and immunogenicity evaluations, except that the follow-up evaluations that are specifically for safety follow-up on a vaccination do not need to be completed when a vaccination is not given. Referring to Appendix III, these are the “A” and “B” and “C” visits that follow vaccinations. Specific events that will require withdrawal of a subject from the vaccination schedule include:

1. HIV infection;
2. Pregnancy;
3. Grade 3 adverse event assessed as possibly, probably or definitely associated with immunization (with the exception that self-limited Grade 3 solicited reactogenicity does not require discontinuation of study injections);
4. Grade 4 adverse event assessed as possibly, probably or definitely associated with immunization;
5. Immediate hypersensitivity reaction associated with immunization;
6. An intercurrent illness that is not expected to resolve prior to the next scheduled immunization and is judged by the study clinician to require withdrawal from vaccination schedule;
7. Treatment with systemic glucocorticoids (e.g., prednisone or other glucocorticoid) or other immunomodulators (other than nonsteroidal anti-inflammatory drugs [NSAIDs]) for any reason;
8. Medical need for concomitant vaccine during the period of study vaccinations that requires discontinuation from the study vaccination schedule (see section 4.6);
9. Repeated failure to comply with protocol requirements;
10. The IND sponsor, study sponsor or Principal Investigator decides to stop or cancel the study;
11. The Institutional Review Board (IRB) or the FDA request that the study be stopped.

4.8 Criteria for Pausing Study and Resuming Study

The Principal Investigator will closely monitor and analyze study data as they become available and will make determinations regarding the presence and severity of adverse events. The DAIDS Medical Officer will provide an independent review of adverse events that have a bearing on study pauses. The administration of study injections and new enrollments will be paused and the IND sponsor will be promptly notified according to the criteria that follow.

One (or more) subject experiences a Grade 4 or Grade 5 adverse event that is assessed as possibly, probably or definitely related to a study vaccine, or

One (or more) subject experiences a Grade 3 urticaria that is assessed as possibly, probably or definitely related to a study vaccine, or

Two (or more) subjects experience the same Grade 3 adverse event assessed as possibly, probably or definitely related to a study vaccine (except that Grade 3 subjective solicited reactogenicity symptoms of injection site pain/tenderness, fatigue/malaise, myalgia, chills, headache or nausea will not result in a study pause).

Plan for Review of Pauses and Resuming Rules:

The study injections and enrollments would resume only if review of the adverse events that caused the pause resulted in a recommendation to permit further study injections and study enrollments. The reviews to make this decision will occur as follows:

The IND Sponsor, in consultation with the Principal Investigator, will conduct the review and make the decision to resume or close the study for the Grade 3 events that meet the criteria for pausing the study. As part of the pause review, the reviewers will also advise on whether the study needs to be paused again for any subsequent Grade 3 event of the same type.

The IND Sponsor, with participation by the Principal Investigator, will consult with the FDA to conduct the review and make the decision to resume or close the study for any Grade 4 and Grade 5 adverse events that meet the criteria for pausing the study.

Safety data reports and changes in study status are submitted to the IRB promptly in accordance with Section 5.4 and institutional policy.

5. SAFETY AND ADVERSE EVENT REPORTING

5.1 ADVERSE EVENTS

An adverse event is any unfavorable or unintended change in body structure, body function or laboratory result associated temporally with the use of study treatment, whether or not considered related to the study treatment. Each adverse event will be graded according to the Table for Grading Severity of Adverse Events (see Appendix IV).

5.2 SERIOUS ADVERSE EVENTS (SAE)

The term "Serious Adverse Drug Experience" is defined in 21 CFR 312.32 as follows: "Any adverse drug experience occurring at any dose that results in any of the following outcomes: Death, a life-threatening adverse drug experience, inpatient hospitalization or prolongation of existing hospitalization, a persistent or significant disability/incapacity, or a congenital anomaly/birth defect. Important medical events that may not result in death, be life-threatening, or require hospitalization may be considered a serious adverse drug experience when, based upon appropriate medical judgment, they may jeopardize the subject or require medical or surgical intervention to prevent one of the outcomes listed in this definition. Examples of such medical events include allergic bronchospasm requiring intensive treatment, blood dyscrasias or convulsions that do not result in inpatient hospitalization, or the development of drug

dependency or drug abuse."

"Life-threatening" refers to an adverse event that at occurrence represents an immediate risk of death to the subject. An event that may cause death if it occurs in a more severe form is not considered life-threatening. Similarly, a hospital admission for an elective procedure is not considered a Serious Adverse Event.

In Section 5.3 the term "Expedited Adverse Event" (EAE) encompasses the events that would be considered an SAE by the 21 CFR 312.32 definition.

5.3 Adverse Event Reporting to the IND Sponsor

The IND Sponsor, DAIDS, issued Version 2.0 of its EAE Manual in January 2010. For this study, the requirements that applied under Version 1.0 were in effect through the last required clinic visit (Week 24 in Part I and Week 52 in Part II) and are updated for the Long-term Follow-up contacts that are part of the schedule for the Part II participants.

All versions of the DAIDS EAE Manual are available on the RSC website: http://rsc.tech-res.com/safetyandpharmacovigilance/.

Regardless of when they occur, AEs reported on an expedited basis must be documented through the DAIDS Adverse Event Reporting System (DAERS) by electronic submission within 3 reporting days of site awareness of the event. Access is available for authorized clinical staff through the RSC website: http://rsc.tech-res.com/safetyandpharmacovigilance/. RSC contact information is provided in Appendix II.

5.3.1 Reporting to the IND Sponsor through Study Week 24 in Part I and Study Week 52 in Part II

Information on adverse events (AEs) is collected by Study Nurses and other clinic staff and entered into a computer database. The Principal Investigator and the Study Coordinator review these data on an ongoing basis.

The EAE reporting requirements and definitions for this study through Study Week 24 in Part I and Study Week 52 in Part II and the methods for expedited reporting of AEs to the DAIDS Regulatory Support Center (RSC) Safety Office are defined in "The Manual for Expedited Reporting of Adverse Events to DAIDS" (DAIDS EAE Manual) Version 1.0, dated May 6, 2004.

EAE Reporting Level:

The study visits through Week 24 in Part I and Week 52 in Part II use the Standard Level of expedited AE reporting as defined in the DAIDS EAE Manual, Version 1.0 (May 6, 2004). Briefly summarized, Standard Level reporting requires completion of an EAE report form for the following types of AEs occurring after exposure to the study agent:

- Result in death regardless of relationship to study agent.
- Are congenital anomalies, birth defects, or fetal losses regardless of relationship to study agent.
- Result in persistent or significant disabilities or incapacities regardless of relationship to study agent.

- Are a suspected adverse drug reaction (i.e., definitely, probably, possibly, or probably not related to study agent) that requires hospitalization, or prolongs existing hospitalization OR requires intervention to prevent significant/permanent disability or death.
- Are life-threatening (including all Grade 4 adverse events) suspected adverse drug reactions (i.e., assessed as definitely, probably, possibly or probably not related to study agent).

In addition, any event, regardless of grade, which in the judgment of a site investigator represents a serious adverse event, may be reported to the IND sponsor as an expedited report.

EAE Reporting Period:

AEs must be reported on an expedited basis at the Standard Level during the protocol-defined EAE Reporting Period, which for this study is from study enrollment until the last required clinical visit for the subject or until discontinuation of the subject from study participation for any reason.

After the end of the protocol-defined EAE reporting period stated above, the site must report serious, unexpected, clinical suspected adverse vaccine reactions if the study site staff becomes aware of the event on a passive basis, i.e. from publicly available information.

Study Agents for Expedited Reporting to DAIDS:

The study agents that must be considered when determining relationships of AEs requiring expedited reporting to DAIDS are: VRC-HIVADV027-00-VP and VRC-HIVADV038-00-VP.

Grading Severity of Events:

The Table for Grading the Severity of Adult Adverse Events is: "The Division of AIDS Table for Grading the Severity of Adult and Pediatric Adverse Events, Version 1.0, Dec 2004" with amendment of the criteria for PTT and seizure (see Appendix IV).

The EAE report must be submitted by the clinical site to the IND sponsor (DAIDS) through the RSC Safety Office (DAIDSRSCSafetyOffice@tech-res.com) as soon as possible, but no later than 3 working days after the clinical site becomes aware of events meeting these criteria. The IND sponsor is responsible for submitting IND safety reports to the FDA, as necessary, per 21 CFR 312.32. DAIDS submits IND safety reports as soon as possible, but no later than 15 days after initial receipt of the information.

5.3.2 Reporting to the IND Sponsor During Long-term Follow-up for Part II

The reporting guidelines in the updated DAIDS EAE Manual, Version 2.0, dated January 2010 apply to Part II subjects starting with the long-term follow-up contacts. For convenience the criteria from the EAE Manual for reporting an AE as a serious adverse event (SAE) are provided in the box below. However, the Manual should be consulted as well for further detail about reporting procedures to be used. Also ensure that any other protocol-specific reporting requirements are met.

Of special note, HIV infection is within the definition of events that need to be recorded during the long-term follow-up period in accordance with protocol **Section 4.2.3**; however, a new diagnosis of HIV infection will not be reported as an EAE, but will be recorded in an HIV diagnosis case report format without a severity grade or attribution assessment.

EAE Reporting Criteria During Long-term follow-up:

Complete an EAE report form for the following adverse events regardless of relationship to study agent:

- Results in death
- Is life-threatening[1]
- Requires inpatient hospitalization or prolongation of hospitalization[2]
- Results in persistent or significant disabilities/incapacity.
- Is a congenital anomaly/birth defect[3]
- Is an important medical event (may jeopardize the patient or may require intervention to prevent one of the outcomes above)

[1] "Life-threatening" refers to an event in which the patient was at risk of death at the time of the event. It does NOT refer to an event that hypothetically might have caused death if it were more severe.

[2] Per ICH SAE definition, hospitalization is NOT an adverse event (AE), but is an outcome of the event. **DO NOT REPORT**: Any admission unrelated to an AE (e.g., for labor/delivery, cosmetic surgery, administrative or social admission for temporary placement for lack of a place to sleep); protocol-specified admission (e.g., for a procedure required by protocol); admission for diagnosis or therapy of a condition that existed before receipt of study agent(s) **and** has not increased in severity or frequency as judged by the clinical investigator. (**NOTE**: A new AIDS-defining event in a subject already known to be HIV-infected would be considered an increase in severity of a pre-existing condition [HIV infection] and **would be** reportable.)

[3] Clinically insignificant physical findings at births including those regarded as normal variants do NOT meet reporting criteria. If a clinically significant anomaly is reported, all findings (including those of no individual significance) should be included in the same report. For example, do NOT report an isolated finding of polydactyly (extra fingers or toes) or Mongolian spot in an infant. But if either finding occurred with a major cardiac defect, report all findings in the SAE Report.

5.3.3 Attribution Categories

Consistent with the EAE Manual, Version 1.0 attribution categories used (i.e. terms used for assessment of relationship of AE to study agent) for reporting AEs to DAIDS through end of the clinical visits are:

- **Definitely Related**. The adverse event and administration of study agent are related in time, and a direct association can be demonstrated.

- **Probably Related**. The adverse event and administration of study agent are reasonably related in time, and the adverse event is more likely explained by study agent than other causes.

- **Possibly Related**. The adverse event and administration of study agent are reasonably related in time, and the adverse event can be explained equally well by causes other than study agent.

- **Probably Not Related**. A potential relationship between study agent and the adverse event could exist (i.e., the possibility cannot be excluded), but the adverse event is most likely explained by causes other than the study agent.

- **Not Related**. The adverse event is clearly explained by another cause not related to the study agent.

Under EAE Manual, Version 2.0 only two attribution categories apply as follows:

- **Related** – There is a reasonable possibility that the AE may be related to the study agent(s).

- **Not Related** – There is not a reasonable possibility that the AE is related to the study agent(s).

In this protocol, the "Definitely, Probably and Possibly" attributions used under EAE Manual, Version 1.0 are considered to map to the "Related" category under EAE Manual, Version 2.0, while the "Probably Not Related and "Not Related" attributions used under EAE Manual, Version 1.0 are considered to both map to the "Not Related" category under EAE Manual, Version 2.0.

5.4 REPORTING TO THE INSTITUTIONAL REVIEW BOARD

5.4.1 Adverse Event Reporting to the NIAID IRB

Adverse event reporting requirements to the NIAID IRB for this protocol are as follows:

- Investigators will submit a completed serious adverse event report to the NIAID IRB within 7 days after becoming aware of a subject death, a potentially life-threatening (Grade 4) serious adverse event that is possibly, probably or definitely related to investigational agent, an inpatient hospitalization (other than elective), a persistent or significant disability/incapacity, or a congenital anomaly/birth defect.

- Investigators will report within 15 days on any other event or condition regardless of grade, which in their judgment represents an event reportable to the IRB.

- Investigators will forward all IND safety reports and related FDA communications to the IRB within 15 days of receipt.

- A summary of all adverse events will be reported to the NIAID IRB with submission of a request for continuing review.

5.4.2 Unanticipated Problem Reporting to the NIAID IRB:

Unanticipated Problem reporting to the NIAID IRB is based upon the OHRP 2007 guidance (http://www.hhs.gov/ohrp/policy/advevntguid.html) and is defined as any incident, experience, or outcome that meets all three of the following criteria:

- unexpected in nature, severity, or frequency in relation to the research risks that are described in the protocol, informed consent, Investigator's Brochure, other study documents or in consideration of the characteristics of the subject population being studied; **and**
- is related to participation in the research; **and**
- places subjects or others at a greater risk of harm (including physical, psychological, economic, or social harm) than was previously known or recognized.

Unanticipated problems meeting these criteria will be reported to the IRB, within 7 days of investigator awareness.

5.4.3 Protocol Violation Reporting to the NIAID IRB

A Protocol Violation is defined as any change, divergence, or departure from the study procedures in an IRB-approved research protocol that meets the criteria for expedited reporting described on the IRB's Protocol Violation Form and has a major impact on the subject's rights, safety, or well-being and/or the completeness, accuracy or reliability of the study data.

The Investigator will report, within 7 days of awareness, any Protocol Violation that meets the IRB's expedited reporting criteria. A summary of all protocol violations will be reported to the NIAID IRB with the annual submission of a request for continuing review.

5.5 SERIOUS ADVERSE EVENT REPORTING TO THE INSTITUTIONAL BIOSAFETY COMMITTEE

The Institutional Biosafety Committee (IBC) (Building 13, Room 3K04, NIH, Bethesda, MD) has a responsibility to review research using recombinant DNA for compliance with NIH Guidelines. In keeping with IBC requirements, any SAE reports sent to the IRB will be provided to the IBC at the same time.

6. STATISTICAL CONSIDERATIONS

6.1 OVERVIEW

This study is a single-center, two part (dose escalation and randomized) trial to assess the safety and tolerability of an intramuscular rAd35-EnvA HIV vaccine in HIV-uninfected adults. A preliminary assessment of immunogenicity will also be performed.

6.2 OBJECTIVES

The primary objective is to evaluate the safety and tolerability in humans of the vaccines. Secondary objectives include evaluating the immunogenicity of the vaccination regimens, the development of adenovirus serotype 35 and serotype 5 neutralizing antibodies and the social impact of participating in an HIV-1 vaccine trial.

6.3 ENDPOINTS

6.3.1 Safety

Assessment of product safety will include clinical observation and monitoring of hematological and chemical parameters. Safety will be closely monitored after injection and evaluated through 24 weeks for subjects in Part I and through 52 weeks for subjects in Part II. See Appendix III for details and specified time points. The following parameters will be assessed:

- Local reactogenicity signs and symptoms
- Systemic reactogenicity signs and symptoms
- Laboratory measures of safety
- Adverse and serious adverse experiences

6.3.2 Immunogenicity

The principal immunogenicity endpoints for cellular immune responses are measured at Week 0 (baseline), 4 weeks after Day 0 vaccinations (in Groups 1, 2, 3, 4A and 4B), and 4 weeks (week 16) after the boost vaccinations (Groups 4A and 4B). They will consist of HIV-1-specific T cell responses, as measured by ELISpot and ICS assays and by research ELISA for vaccine-specific antigens. These and other immunogenicity assays will be performed at other study timepoints as exploratory evaluations.

6.3.3 Social Impacts

Social impact variables, as measured by questionnaire at the last clinic visit, include any negative experiences or problems the participant experienced due to his/her participation in this study. The following social impacts will be followed during the course of the study: personal relationships, travel or immigration, employment, education, medical or dental care, health insurance, life insurance, housing, military/other government agency and other impacts identified by a participant.

6.4 SAMPLE SIZE AND ACCRUAL

Recruitment will target 35 healthy, HIV-uninfected adult participants between age 18 and 50 years old. The required clinic visits are through Study Week 24 for subjects in Part I and through Study Week 52 for subjects in Part II. Sample size will be 15 for subjects with safety data for the dose escalation groups and 20 for the rAd35 prime-rAd5 boost/rAd5 prime-rAd35 boost combination regimens. The sample sizes for each arm are as follows:

Group 1	**rAd35-EnvA** (10^9 PU IM)	N=5
Group 2	**rAd35-EnvA** (10^{10} PU IM)	N=5
Group 3	**rAd35-EnvA** (10^{11} PU IM)	N=5
Group 4A	**rAd35-EnvA** (10^{10} PU IM), **rAd5-EnvA** (10^{10} PU IM)	N=5
Group 4B	**rAd 5-EnvA** (10^{10} PU IM), **rAd35-EnvA** (10^{10} PU IM)	N=5
Group 4C	**rAd35-EnvA** (10^{11} PU IM), **rAd5-EnvA** (10^{10} PU IM)	N=5
Group 4D	**rAd 5-EnvA** (10^{10} PU IM), **rAd35-EnvA** (10^{11} PU IM)	N=5

6.4.1 Randomization of Treatment Assignments

Participants will be sequentially enrolled into Groups 1, 2, 3, and 4 in the order in which they are found to be both eligible and available to begin participation. Groups 1, 2 and 3 will not be randomized or blinded to assignment, but rather will be assigned in a sequential manner to allow

the assessment of the safety of each dose before proceeding to the next. Study numbers 01012001 through 01012015 will be sequentially assigned to participants as they enroll into Groups 1, 2 and 3.

Group 4 assignments will be randomized and blinded, with the first 10 subjects randomized between Groups 4A and 4B and the second 10 subjects randomized between Groups 4C and 4D. Study numbers 01012016 through 01012025 will be used for the randomization of subjects in Groups 4A and 4B and study numbers 01012026 through 01012035 will be used for the randomization of subjects in Groups 4C and 4D. The randomization sequence will be obtained by computer-generated random numbers and provided to the study pharmacist by the statistician. The pharmacist and the statistician are responsible for maintaining security of the treatment assignments. To maintain blinding, any discussion of the treatment assignment between the VRC clinicians and the pharmacy staff or statistician is prohibited until after the assignments are permitted to be known to all. To decrease the potential for participant dropouts during the period between randomization and initial vaccination, randomization will occur on Day 0 after the study consent is signed and eligibility is confirmed. The study number is assigned through completion of the eligibility checklist in the electronic study database and will be the next sequential number in the study number sequence.

6.4.2 Power Calculations for Safety

The goal of the safety evaluation for this study is to identify safety concerns associated with injection. Sample size calculations for safety are expressed in terms of the ability to detect serious adverse experiences.

The ability of the study to identify serious adverse experiences is best expressed by the maximum true rate of events that would be unlikely to be observed and the minimum true rate of events that would very likely be observed. Specifically, there is a 83% chance of observing at least 1 serious adverse experience in the 5 volunteers receiving a specific dose if the true rate of such an event is at least 0.3; there is a 77% chance that we would not observe at least 1 serious adverse experience if the true rate is less than 0.05. Across the entire study, there is a 83% chance of observing at least 1 serious adverse experience in the 35 volunteers if the true rate of such an event is at least 0.05. Probabilities of observing 0 or 2 or more serious adverse experiences among the total sample size (N=35), and within each arm for Part I (N=5) are presented in Table 6.1 for a range of possible true event rates. These calculations provide a more complete picture of the sensitivity of this study design to identify potential safety problems with the vaccine.

Table 6.1: Probability of Response for Different Safety and Immunogenicity Scenarios

	N=35		N=5	
True Rate	Pr(0/35)	Pr(2+/35)	Pr(0/5)	Pr(2+/5)
.05	0.17	0.53	0.77	0.02
.1	0.03	0.88	0.59	0.08
.15	<0.01	0.98	0.44	0.16
.2	<0.01	>0.99	0.33	0.26
.3	<0.01	>0.99	0.17	0.47

Table 6.2 gives the upper and lower bounds for 95% exact binomial confidence intervals for several possible numbers of events. For example, if none of the 35 participants receiving the

vaccine experience serious adverse experiences to the vaccine, the 95% exact 2-sided upper confidence bound for the rate of such reactions in the population is 0.10. Within a group of 5, the confidence interval would range from 0 to 0.52.

Table 6.2: 95% Confidence Intervals for Some Possible Observed Rates

	95% CI		95% CI
**0/35**	0,0.10	**0/5**	0,0.52
**1/35**	0,0.15	**1/5**	0,0.72
**2/35**	0.01,0.19	**2/5**	0.05,0.85
**3/35**	0.02,0.23	**3/5**	0.15,0.95
**4/35**	0.03,0.27	**4/5**	0.28,0.99
**5/35**	0.05, 0.30	**5/5**	0.48,1
**10/35**	0.15, 0.46		
**15/35**	0.26, 0.61		
**20/35**	0.39, 0.74		
**25/35**	0.54, 0.85		
**30/35**	0.70, 0.95		

6.4.3 Sample Size Calculations for Immunogenicity

The primary goal of this trial regarding immunogenicity outcomes is a preliminary estimation of response rates. The definition of response is based on comparing the percent of responding cells when stimulated to the background levels specific for each person at each time point. Table 6.2 is also applicable to the immunogenic response rates, and gives the exact 95% confidence interval for possible numbers of responses among the different groups of volunteers. For example, if we observe 4 responses among the 5 vaccinees receiving VRC-HIVADV027-00-VP at a dose of 10^{10} PU, our 95% exact binomial confidence interval for the true rate will range from 0.28 to 0.99.

6.5 STATISTICAL ANALYSIS

Since enrollment is concurrent with receiving the first study vaccination, all participants will have received at least one vaccination and therefore will provide some safety data. All statistical analyses will be performed using SAS and S-Plus statistical software. No formal multiple comparison adjustments will be employed for safety endpoints or secondary endpoints.

6.5.1 Analysis Variables

The analysis variables consist of baseline variables, safety variables, immunogenicity and social impact variables for primary and secondary objective analyses.

6.5.2 Baseline Demographics

Baseline characteristics including demographics and laboratory measurements will be summarized using descriptive statistics.

6.5.3 Safety Analysis

Reactogenicity

The number and percentage of participants experiencing each type of reactogenicity sign or symptom will be tabulated by severity. For a given sign or symptom, each participant's reactogenicity will be counted once under the maximum severity for all assessments. Reactogenicities for each dose group in Part I will be tabulated separately; similarly after Part II is unblinded the reactogenicity by vaccine type and dose will be tabulated separately for prime and boost injections.

Adverse Experiences

Adverse experiences (AEs) are coded into MedDRA preferred terms. The number and percentages of participants experiencing each specific adverse event will be tabulated by severity and relationship to treatment. For the calculations in these tables, each participant's adverse experience will be counted once under the maximum severity or strongest recorded causal relationship to treatment.

Adverse experiences following vaccination with VRC-HIVADV027-00-VP will be summarized separately from AEs following vaccination with VRC-HIVADV038-00-VP. A complete listing of adverse experiences for each participant will provide details including severity, relationship to treatment type, onset, duration and outcome.

Local laboratory values

Boxplots of local laboratory values will be generated for baseline values and for values measured during the course of the study. Each boxplot will show the 1st quartile, the median, and the 3rd quartile. Outliers, or values outside the boxplot, will also be plotted. If appropriate, horizontal lines representing boundaries for abnormal values will be plotted.

6.5.4 Immunogenicity Analysis

The statistical analysis for immunogenicity will employ the intent-to-treat principle, i.e., all data from enrolled participants will be used. The only exception will be to exclude data from HIV-infected participants at or post infection. If the HIV positivity status of an infected participant is unknown at the time that the first sample for immunogenicity assessments is drawn, then all data from that participant will be excluded from the analysis.

If assay data are qualitative (i.e., positive or negative) then analyses will be performed by tabulating the frequency of positive response for each assay at each time point that an assessment is performed. Binomial response rates will be presented with their corresponding exact 95% confidence interval estimates. Response rates by strata, injection method and Ad boost will be summarized in contingency tables and compared using Fisher's exact test. Missing responses will be assumed to be missing at random, i.e., conditional on the observed data the missingness is independent of the unobserved responses. Graphical descriptions of the longitudinal immune

responses will also be given.

Some immunologic assays have underlying continuous or count-type readout that is often dichotomized into responder/nonresponder categories. For these assays, graphical and tabular summaries of the underlying distributions will be made. These summaries may be performed on transformed data (e.g., log transformation) to better satisfy assumptions of symmetry and homoscedasticity.

6.5.5 Social Impact Analysis

Social impacts will be tabulated by type of event and impact on quality of life. The number and percentage of participants experiencing each type of social impact will also be tabulated by impact on quality of life. For this calculation multiple events of the same type for a participant will be counted once under the maximum impact for all post-vaccination visits.

In addition, a listing will be generated of all participants who experienced a major disturbance of their quality of life due to study participation. The listing includes all social impacts experienced by these participants, descriptions of each impact, impact on quality of life and whether or not there was a resolution.

6.5.6 Interim Analyses

Interim analyses of immunogenicity for each Group may be performed after all ICS assays up to and including 4 weeks after completion of the priming vaccination have been completed on all participants in a Group and again 4 weeks after completion of the booster vaccinations have been completed on all participants in Group 4. The purpose of the reports is to provide basic immunogenicity data to inform those who are making future clinical trial development-related decisions in a timely manner. The results of this interim immunogenicity analysis will not influence the conduct of the VRC 012 trial in terms of early termination or completion of later safety or immunogenicity endpoint assessments.

6.5.7 Unblinding Group 4 Assignments and Emergency Unblinding

The Group 4 assignments will be unblinded when safety data collection through 4 weeks after the booster injection for all subjects is completed.

If the Principal Investigator (or designee) and the DAIDS Medical Officer agree that management of an adverse event requires emergency unblinding of an individual subject's treatment assignment, then the Pharmacist will be asked to provide the group assignment for that subject. This will be documented and the Protocol Statistician, the IRB and the DSMB will be notified that an early unblinding has occurred and provided with a statement explaining the medical necessity for the early unblinding.

7. PHARMACY PROCEDURES

The study vaccine regimens are shown in Tables 4.1 and 4.2. Refer to the Investigator's Brochure for further information about study products. Quality Assurance lot release testing by the manufacturer verifies conformance to product specifications prior to use in clinical trials.

7.1 STUDY AGENTS

The VRC 012 study includes two investigational adenoviral vector vaccines that both encode for a clade A Env antigen; one is an adenovirus serotype 35 vector and the other is an adenovirus

serotype 5 vector. A diluent is also provided to use in preparation of the lowest dosage of the rAd35 vaccine. There are no placebo injections in the study.

- rAd35-EnvA 1 x 10^{10} PU/mL: VRC-HIVADV027-00-VP
- rAd35-EnvA 1 x 10^{11} PU/mL: VRC-HIVADV027-00-VP
- rAd5-EnvA 1 x 10^{10} PU/mL: VRC-HIVADV038-00-VP
- rAd35 Diluent: VRC-DILUENT013-DIL-VP (final formulation buffer, FFB)

Vials will be individually labeled with specific manufacturing and storage information. If deviations in storage temperature occur, the site pharmacist must report the storage temperature excursion promptly. The excursion must be evaluated and investigated and action must be taken to restore and maintain the desired temperature limits. Pending the outcome of the investigation, the IND sponsor will notify the pharmacist if continued clinical use of the product is acceptable.

Vials of final filled drug product will be sent to a cGMP-compliant storage and distribution facility. Upon release, the vials will be shipped on dry ice in sealed Mylar bags (to prevent CO_2 from inactivating the adenoviral product) to the recipient pharmacy and will be stored at temperatures as indicated on the vial label. Vials of vaccine and the diluent are intended for single use only and should not be refrozen after thawing.

7.1.1 Adenoviral Vector Serotype 35 Vaccine, VRC-HIVADV027-00-VP

The rAd35-EnvA vaccine is supplied in sterile 3 mL vials filled to a target volume of 1.2 ± 0.1 mL at a concentration of 1 x 10^{10} PU/mL or 1 x 10^{11} PU/mL. The vaccine is formulated as a clear, colorless, frozen solution in a stabilizing buffering agent, a disaccharide, salts and a nonionic surfactant. The container closure system is a 3 mL borosilicate glass vial, butyl rubber stopper and aluminum seal.

7.1.2 Adenoviral Vector Serotype 5 Vaccine, VRC-HIVADV038-00-VP

The rAd5-EnvA vaccine is supplied in sterile 3 mL vials filled to a target volume of 1.2 ± 0.1 mL at a concentration of 1 x 10^{10} PU/mL. The vaccine is formulated as a clear, colorless, frozen solution in a stabilizing buffering agent, a disaccharide, salts and a nonionic surfactant. The container closure system is a 3 mL borosilicate glass vial, butyl rubber stopper and aluminum seal.

7.1.3 Diluent, VRC-DILUENT013-DIL-VP

The diluent is the final formulation buffer, VRC-DILUENT013-DIL-VP (FFB), which is composed of sodium chloride, Tris buffer, trehalose $\bullet 2H_2O$ (low endotoxin), magnesium chloride$\bullet 6H_2O$, monooleate (Tween 80) and water for injection (WFI). The FFB diluent is a clear, colorless solution supplied in a 3 mL glass vial filled to a target volume of 1.2 ± 0.1 mL.

7.2 PREPARATION OF STUDY AGENT FOR ADMINISTRATION

To prepare a study injection, the pharmacist will remove the appropriate vial(s) from the freezer and allow to equilibrate to room temperature. Each study injection must be administered within

4 hours after removing the vials from the freezer.

7.2.1 Preparation of 10^9 PU Dosage of VRC-HIVADV027-00-VP for Administration by Needle and Syringe

To prepare an injection of the 1 x 10^9 PU dose, the study pharmacist, will use an appropriate dilution method as follows:

- Using a 5 mL sterile syringe, draw up 4 mL of the diluent, VRC-DILUENT013-DIL-VP and inject into an empty 10 mL sterile vial. Also draw up 0.5 mL of the diluent with a 1 mL sterile syringe and add to the 10 mL vial to achieve a total volume of 4.5 mL diluent in a 10 mL vial.
- Using a sterile 1 mL syringe, withdraw 0.5 mL of the investigational vaccine, VRC-HIVADV027-00-VP, from a thawed vial containing 1 x 10^{10} PU/mL and inject into the 10 mL vial that contains 4.5 mL of the diluent. Vortex at half speed for 3-5 seconds. This vial now contains VRC-HIVADV027-00-VP at 1 x 10^9 PU/mL (5 x 10^9 PU in 5 mL).
- The pharmacy will prepare an individual syringe with 1 mL volume from the 1 x 10^9 PU/mL vial and label it with the subject identifier for transport to the clinic.

7.2.2 Preparation of 10^{10} PU Dosage of VRC-HIVADV027-00-VP for Administration by Needle and Syringe

To prepare a rAd35-EnvA 10^{10} PU IM injection, the pharmacy will prepare an individual syringe with 1 mL volume from a 1 x 10^{10} PU/mL vial and label it with the subject identifier for transport to the clinic.

7.2.3 Preparation of 10^{11} PU Dosage of VRC-HIVADV027-00-VP for Administration by Needle and Syringe

To prepare a rAd35-EnvA 10^{11} PU IM injection, the pharmacy will prepare an individual syringe with 1 mL volume from a 1 x 10^{11} PU/mL vial and label it with the subject identifier for transport to the clinic.

7.2.4 Preparation of 10^{10} PU Dosage of VRC-HIVADV038-00-VP for Administration

To prepare a rAd5-EnvA 10^{10} PU IM injection, the pharmacy will prepare an individual syringe with 1 mL volume from a 1 x 10^{10} PU/mL vial and label it with the subject identifier for transport to the clinic.

7.3 STUDY AGENT LABELING

Vials will be individually labeled with the name of the material, dose, pH, volume, lot number, concentration, storage instructions, Investigational Use Statement ("Caution: New Drug – Limited by Federal Law to Investigational Use"), and manufacturer information.

7.4 PROCEDURES TO PRESERVE BLINDING IN PART II

There are four vaccination schedules in Part II (Group 4). The subjects, the clinical staff, and the Principal Investigator will be blinded to treatment allocation through 4 weeks after the booster injection for all subjects is completed. The pharmacist with primary responsibility for vaccine

dispensing receives the randomization code from the protocol statistician in advance of opening the study. Each time a vaccine order is sent to the pharmacy by a study clinician, the pharmacist checks to ensure that the vaccine dispensed matches the schedule assignment for that subject's study ID. The study pharmacist will be responsible for preparing the syringe with the subject identification. The pharmacist will not be the same individual who is responsible for clinical follow-up.

7.5 Study Agent Accountability

7.5.1 Documentation

The site pharmacist will be responsible for maintaining an accurate record of the codes, inventory, and an accountability record of vaccine supplies for this study. The site pharmacist will also be responsible for ensuring the security of these documents. Electronic as well as paper records may be used.

7.5.2 Disposition

The empty vials and the unused portion of a vial will be discarded in a biohazard containment bag and incinerated or autoclaved. Any unopened vials that remain at the end of the study will be returned to the production facility or discarded at the discretion of the sponsor in accordance with policies that apply to investigational agents. Partially used vials will not be administered to other subjects or used for *in vitro* experimental studies. They will be disposed of in accordance with institutional or pharmacy policy.

8. HUMAN SUBJECT PROTECTIONS AND ETHICAL OBLIGATIONS

This research study will be conducted in compliance with the protocol, Good Clinical Practices (GCP), and all applicable regulatory requirements.

8.1 Informed Consent

The study informed consent is provided in Appendix I. It describes the investigational product to be used and all aspects involved in protocol participation.

Before a subject's participation in the study, it is the investigator's responsibility to obtain written informed consent from the subject, after adequate explanation of the aims, methods, anticipated benefits, and potential hazards of the study and before any protocol-specific procedures or study medications are administered.

The acquisition of informed consent will be documented in the subject's medical records, as required by 21 CFR 312.62. The informed consent form will be signed and personally dated by the subject and the person who conducted the informed consent discussion. The original signed informed consent form will be retained in the medical chart and a copy will be provided to the subject.

8.2 Risks and Benefits

8.2.1 Risks

VRC-HIVADV027-00-VP and VRC-HIVADV038-00-VP: At the Vaccine Research Center at the National Institutes of Health, similar adenoviral vector vaccines have been studied in Phase I

trials. The first extramural study using a similar vaccine opened in November 2004. At doses equal or lower than 10^{10} PU dose none of the subjects in the first Phase I study had fever and the other reactogenicity was mild or none. At the next higher dose (10^{11} PU) four subjects had a flu-like set of symptoms with fever, headache, muscle aches, malaise and chills starting 12-16 hours after vaccination and lasting a few hours. Some of these symptoms were moderate in severity. A few subjects have had nausea. Some subjects have had injection site pain or discomfort in the first few days after a vaccination. These symptoms improved after treatment with over-the-counter medicine.
A potential safety concern raised by the MRK-rAd5 vaccine is that pre-existing Ad5 immunity from a natural adenovirus serotype 5 infection may increase susceptibility to HIV infection if exposure to HIV occurs subsequently (see Section 1.2.1) [21].

The effect of the study vaccines on a fetus or nursing baby is unknown, so female subjects of child bearing potential will be required to agree to use birth control for sexual intercourse beginning 21 days prior to enrollment and continuing through 12 weeks after the last vaccination. Women who are pregnant or nursing will be excluded from the study.

Either vaccine may cause a positive HIV antibody test using the standard screening test. A positive or indeterminate test may have a negative employment and social impact. Western blot analysis and HIV PCR or other testing will be done to either exclude or confirm HIV infection. ELISA, Western Blot, and PCR results will be discussed with the study subject as they become available.

Blood drawing may cause pain or bruising, may infrequently cause a feeling of lightheadedness or fainting and, rarely, may cause infection at the site where the blood is taken.

Subjects may believe that this vaccine provides protection, and therefore practice riskier behavior. They will receive extensive counseling throughout the study to address this potential problem.

8.2.2 Benefits

It is unknown if any benefit will result from study participation. Others may benefit from knowledge gained in this study that may aid in the development of an HIV vaccine.

8.3 INSTITUTIONAL REVIEW BOARD

A copy of the protocol, proposed informed consent form, other written subject information, and any proposed advertising material will be submitted to the IRB for written approval.

The investigator must submit and, where necessary, obtain approval from the IRB for all subsequent protocol amendments and changes to the informed consent document. The investigator will notify the IRB of deviations from the protocol and serious adverse events.

The investigator will be responsible for obtaining IRB approval of the annual Continuing Review throughout the duration of the study.

8.4 PROTOCOL REGISTRATION

The Division of AIDS, NIAID is the IND sponsor for this protocol. Protocol registration must occur before subjects are enrolled in this study. The IRB must approve the protocol and consent form. The protocol must be submitted to the IBC. Approval letters from both the IRB and IBC must be submitted to the Division of AIDS RCC Protocol Registration Office (see Appendix II)

with the initial protocol registration. Subsequent protocol amendments must also be registered with and approved by the RCC Protocol Registration Office.

8.5 Subject Confidentiality

The investigator must ensure that the subject's anonymity is maintained. Individual identifying information will not be included in any reports; subjects will be identified only by coded numbers. All records will be kept confidential to the extent provided by federal, state and local law. Medical records are made available for review when required by the Food and Drug Administration or other authorized users, such as the vaccine manufacturer, only under the guidelines set by the Federal Privacy Act. Direct access includes examining, analyzing, verifying, and reproducing any records and reports that are important to the evaluation of the study. The investigator is obligated to inform the subjects that the above named representatives will review their study-related records without violating the confidentiality of the subjects.

8.6 Plan for Use and Storage of Biological Samples

The June 12, 2006 memorandum "Research Use of Stored Human Samples, Specimens or Data" requires that all NIH IRB-approved protocols in which intramural research program researchers intend to collect and store human specimens or data must include a written description of the intended use of the samples; how they will be stored; how they will be tracked; what will happen to at the completion of the protocol, and what circumstances would prompt the PI to report to the IRB loss or destruction of samples. We will apply the specified provisions to the stored samples from this protocol as follows:

<u>Intended use of the samples/specimens/data</u>:
Samples, specimens and data collected under this protocol may be used to conduct protocol-related safety and immunogenicity evaluations, exploratory laboratory evaluations related to the type of infection the vaccine was designed to prevent, exploratory laboratory evaluations related to vaccine research in general and for research assay validation. Genetic testing may be performed in accordance with the genetic testing information that was included in the study informed consent.

<u>How stored samples, specimens and data from sample use will be stored</u>:
All of the stored study research samples are labeled by a code (such as a number) that only the VRC Clinic can link to the subject. Samples are stored at the NIAID Vaccine Immune T-Cell and Antibody Laboratory (NVITAL), Gaithersburg, MD or VRC Laboratories in Building 40, which are both secure facilities with limited access. Data will be kept in password-protected computers. Only investigators or their designees will have access to the samples and data.

<u>How samples/specimens/data will be tracked</u>:
Samples will be tracked in the Laboratory Information Management System (LIMS) database.

<u>What will happen to the samples/specimens/data at the completion of the protocol</u>:
In the future, other investigators (both at NIH and outside) may wish to study these samples and/or data. IRB approval must be sought prior to any sharing of samples with investigators and any clinical information shared about those samples would similarly require prior IRB approval. The research use of stored, unlinked or unidentified samples may be exempt from the need for

prospective IRB review and approval. Exemption requests will be submitted in writing to the NIH Office of Human Subjects Research, which is authorized to determine whether a research activity is exempt.

At the time of protocol termination, samples will remain in the NVITAL facility or VRC laboratories or, after IRB approval, transferred to another repository. Data will be archived by the VRC in compliance with requirements for retention of research records, or after IRB and study sponsor approval, it may be either destroyed or transferred to another repository.

Circumstances that would prompt the PI to report loss or destruction of samples/specimens/data to the IRB:
The NIH Intramural Protocol Violation definition related to loss of or destruction of samples will be followed in reporting to the IRB. Any loss or unanticipated destruction of samples (for example, due to freezer malfunction) or data (for example, misplacing a printout of data with identifiers) that compromises the scientific integrity of the study will be reported to the IRB. The PI will also notify the IRB if the decision is made to destroy the remaining samples.

8.7 Subject Identification and Enrollment of Study Participants

All study activities will be carried out at the Clinical Center at the National Institutes of Health. Study subjects will be recruited through on-site and off-site advertising done for the screening protocol, VRC 000 (02-I-0127). Effort will be made to include women and minorities in proportions similar to that of the community from which they are recruited. Because this Phase I study is designed to establish safety of the vaccine in healthy adults, enrollment will be limited to persons at least 18 years of age, and no older than 50 years of age.

8.7.1 Participation of Children

Children are not eligible to participate in this clinical trial because it does not meet the guidelines for inclusion of children in research. These guidelines (45 CFR 46, Subpart D, 401-409), state the Department of Health and Human Services protections for children who participate in research. Generally, healthy children can be studied when the research is considered as "not greater than minimal risk." Children can be involved in research with greater than minimal risk only when it presents the prospect of direct benefit to the individual child or is likely to yield generalizable knowledge about the child's disorder or condition.

8.8 Compensation

Subjects will be compensated for time and inconvenience in accordance with the standards for compensation of the Clinical Research Volunteer Program. The compensation per visit will be $275 for visits that include injections and blood drawing, $175 for visits that include blood drawing but no injection, and $75 for unscheduled visits without blood draws. The approximate total compensation for the subject will be between $975 (for Part I) and $1775 (for Part II), based on the projected number of clinic visits and study injections for Part I and Part II, respectively. Part II subjects who complete the annual long-term contacts as clinic visits will be compensated $175 if completed as a clinic visit with blood drawing. Subjects may complete HIV testing at unscheduled intervals to address social and medical needs for test results, but are not compensated for these extra visits. Amendment of study compensation will not be retroactive, but will be applied only to activities taking place after approval.

8.9 SAFETY MONITORING

Close cooperation between the designated members of the Protocol Team will occur to evaluate and respond to individual adverse events in a timely manner. The VRC Safety Officer for the day (nurse practitioner or physician) conducts a daily safety review of clinical data, including laboratory results per VRC Clinic guidelines. Designated team members (Principal Investigator, Associate Investigators, Medical Officer, Protocol Specialist, Study Coordinator and other study clinicians) will review the summary study safety data reports on a weekly basis through 4 weeks after the last subject receives the last study injection in order to be certain that the vaccine has an acceptable safety profile and will continue to monitor the study safety data reports on a monthly basis through completion of the last study visit. The DAIDS Medical Officer will provide an independent review of adverse events that have a bearing on study stopping.

The NIAID Intramural DSMB safety monitoring reviews will be scheduled to occur as specified in Section 4.6.

9. ADMINISTRATION AND LEGAL OBLIGATION

9.1 PROTOCOL AMENDMENTS AND STUDY TERMINATION

Protocol Amendments must be made only with the prior approval of the National Institute of Allergy and Infectious Diseases' Division of AIDS and Vaccine Research Center. Agreement from the investigator must be obtained for all protocol amendments and amendments to the informed consent document. All study amendments will be submitted to the IRB for approval.

The Division of AIDS, National Institute of Allergy and Infectious Diseases, the Vaccine Research Center, the Principal Investigator, the Institutional Review Board, the Office of Human Research Protection and the Food and Drug Administration reserve the right to terminate the study. The investigator will notify the IRB in writing of the study's completion or early termination.

9.2 STUDY DOCUMENTATION AND STORAGE

The investigator will maintain a list of appropriately qualified persons to whom trial duties have been delegated.

Source documents are original documents, data, and records from which the subject's data are obtained. These include but are not limited to hospital records, clinical and office charts, laboratory and pharmacy records, microfiches, radiographs, and correspondence. Solicited reactogenicity data will be entered into electronic case report forms and the source and accuracy of the information verified by study clinicians.

The investigator and staff are responsible for maintaining a comprehensive and centralized filing system of all study-related (essential) documentation, suitable for inspection at any time by representatives from the National Institute of Allergy and Infectious Diseases' Division of AIDS and Vaccine Research Center, IRB, FDA, and/or applicable regulatory authorities. Elements include:

- Subject files containing completed informed consent forms, and supporting copies of source documentation (if kept)
- Study files containing the protocol with all amendments, Investigator Brochures,

copies of all correspondence with the IRB and the National Institute of Allergy and Infectious Diseases' Division of AIDS and Vaccine Research Center

In addition, all original source documentation must be maintained and be readily available.

All essential documentation should be retained by the institution for the same period of time required for medical records retention. After study closure, the code that could potentially link study sample numbers to subject identity will be maintained in a secure database. The FDA requires study records to be retained for up to two years after marketing approval or refusal (21 CFR 312.62). No study document should be destroyed without prior written agreement between the National Institute of Allergy and Infectious Diseases' Division of AIDS and Vaccine Research Center and the investigator. Should the investigator wish to assign the study records to another party or move them to another location, they must notify the National Institute of Allergy and Infectious Diseases' Division of AIDS and Vaccine Research Center in writing of the new responsible person and/or the new location.

9.3 STUDY MONITORING, DATA COLLECTION, AND DATA MONITORING

9.3.1 Study Monitoring

The National Institute of Allergy and Infectious Diseases' Division of AIDS and Vaccine Research Center regulatory authority inspectors or their authorized representatives are responsible for contacting and visiting the investigator for the purpose of inspecting the facilities and, upon request, inspecting the various records of the trial, provided that subject confidentiality is respected.

Site visits by study monitors will be made in accordance with the IND Sponsor (DAIDS) policy to monitor the following: study operations, the quality of data collected in the research records, the accuracy and timeliness of data entered in the database, and to determine that all process and regulatory requirements are met.

Site investigators will allow the study monitors, the NIAID IRB, and the FDA to inspect study documents (e.g., consent forms, drug distribution forms, case report forms) and pertinent hospital or clinic records for confirmation of the study data.

9.3.2 Data Collection

Clinical research data will be collected in a secure electronic data management system through a contract research organization, EMMES (Rockville, MD). Extracted data without patient identifiers will be sent to the Protocol Statistician for statistical analysis.

9.4 LANGUAGE

All written information and other material to be used by subjects and investigative staff must use vocabulary and language that are clearly understood.

9.5 POLICY REGARDING RESEARCH-RELATED INJURIES

The Clinical Center will provide short-term medical care for any injury resulting from participation in this research. In general, the National Institutes of Health, the Clinical Center, or the Federal Government will provide no long-term medical care or financial compensation for research-related injuries.

10. REFERENCES

1. UNAIDS/WHO. *AIDS Epidemic Update - December 2004*. 2004 [cited; Available from: http://www.unaids.org/wad2004/report_pdf.html.

2. UNAIDS. *2006 Report on the Global AIDS Epidemic*. 2006 [cited; Available from: http://www.unaids.org/en/HIV_data/2006GlobalReport/default.asp.

3. WHO, *Treating 3 Million by 2005: The WHO Strategy*. 2003, World Health Organization: Geneva, Switzerland. p. 1-53.

4. Barouch, D.H. and G.J. Nabel, *Adenovirus vector-based vaccines for human immunodeficiency virus type 1*. Hum Gene Ther, 2005. 16(2): p. 149-56.

5. Casimiro, D.R., L. Chen, T.M. Fu, R.K. Evans, M.J. Caulfield, et al., *Comparative immunogenicity in rhesus monkeys of DNA plasmid, recombinant vaccinia virus, and replication-defective adenovirus vectors expressing a human immunodeficiency virus type 1 gag gene*. J Virol, 2003. 77(11): p. 6305-13.

6. Letvin, N.L., Y. Huang, B.K. Chakrabarti, L. Xu, M.S. Seaman, et al., *Heterologous envelope immunogens contribute to AIDS vaccine protection in rhesus monkeys*. J Virol, 2004. 78(14): p. 7490-7.

7. Santra, S., M.S. Seaman, L. Xu, D.H. Barouch, C.I. Lord, et al., *Replication-defective adenovirus serotype 5 vectors elicit durable cellular and humoral immune responses in nonhuman primates*. J Virol, 2005. 79(10): p. 6516-22.

8. Shiver, J.W. and E.A. Emini, *Recent advances in the development of HIV-1 vaccines using replication-incompetent adenovirus vectors*. Annu Rev Med, 2004. 55: p. 355-72.

9. Shiver, J.W., T.M. Fu, L. Chen, D.R. Casimiro, M.E. Davies, et al., *Replication-incompetent adenoviral vaccine vector elicits effective anti-immunodeficiency-virus immunity*. Nature, 2002. 415(6869): p. 331-5.

10. Graham, B.S., R.A. Koup, M. Roederer, R.T. Bailer, M.E. Enama, et al., *Phase 1 safety and immunogenicity evaluation of a multiclade HIV-1 DNA candidate vaccine*. J Infect Dis, 2006. 194(12): p. 1650-60.

11. Isaacs, R., *Evaluating the efficacy of the Merck adenovirus serotype 5-based trivalent MRKAD5GAG/POL/NEF (paper #42)*, in *AIDS Vaccine 2005*. 2005: Montreal, Canada.

12. Shiver, J.W., *Development of an HIV-1 vaccine based on replication-defective adenovirus*, in *Keystone Symposium on HIV Vaccine Development: Process and Prospects*. 2004: Whistler, B.C., Canada.

13. Catanzaro, A.T., R.A. Koup, M. Roederer, R.T. Bailer, M.E. Enama, et al., *Phase 1 safety and immunogenicity evaluation of a multiclade HIV-1 candidate vaccine delivered by a replication-defective recombinant adenovirus vector.* J Infect Dis, 2006. 194(12): p. 1638-49.

14. Jin, X., D.E. Bauer, S.E. Tuttleton, S. Lewin, A. Gettie, et al., *Dramatic rise in plasma viremia after CD8(+) T cell depletion in simian immunodeficiency virus-infected macaques.* J Exp Med, 1999. 189(6): p. 991-8.

15. Klein, M.R., C.A. van Baalen, A.M. Holwerda, S.R. Kerkhof Garde, R.J. Bende, et al., *Kinetics of Gag-specific cytotoxic T lymphocyte responses during the clinical course of HIV-1 infection: a longitudinal analysis of rapid progressors and long-term asymptomatics.* J Exp Med, 1995. 181(4): p. 1365-72.

16. Moss, R.B., E. Webb, W.K. Giermakowska, F.C. Jensen, J.R. Savary, et al., *HIV-1-Specific CD4 helper function in persons with chronic HIV-1 infection on antiviral drug therapy as measured by ELISPOT after treatment with an inactivated, gp120-depleted HIV-1 in incomplete Freund's adjuvant.* J Acquir Immune Defic Syndr, 2000. 24(3): p. 264-9.

17. Musey, L., J. Hughes, T. Schacker, T. Shea, L. Corey, et al., *Cytotoxic-T-cell responses, viral load, and disease progression in early human immunodeficiency virus type 1 infection.* N Engl J Med, 1997. 337(18): p. 1267-74.

18. Nixon, D.F., A.R. Townsend, J.G. Elvin, C.R. Rizza, J. Gallwey, et al., *HIV-1 gag-specific cytotoxic T lymphocytes defined with recombinant vaccinia virus and synthetic peptides.* Nature, 1988. 336(6198): p. 484-7.

19. Schmitz, J.E., M.J. Kuroda, S. Santra, V.G. Sasseville, M.A. Simon, et al., *Control of viremia in simian immunodeficiency virus infection by CD8+ lymphocytes.* Science, 1999. 283(5403): p. 857-60.

20. Yang, O., S.A. Kalams, M. Rosenzweig, A. Trocha, N. Jones, et al., *Efficient lysis of human immunodeficiency virus type 1-infected cells by cytotoxic T lymphocytes.* J Virol, 1996. 70(9): p. 5799-806.

21. Cohen, J., *Did Merck's Failed HIV Vaccine Cause Harm.* Science, 2007. 318(5852): p. 1048-9.

22. Kostense, S., W. Koudstaal, M. Sprangers, G.J. Weverling, G. Penders, et al., *Adenovirus types 5 and 35 seroprevalence in AIDS risk groups supports type 35 as a vaccine vector.* Aids, 2004. 18(8): p. 1213-6.

23. Vogels, R., D. Zuijdgeest, R. van Rijnsoever, E. Hartkoorn, I. Damen, et al., *Replication-deficient human adenovirus type 35 vectors for gene transfer and vaccination: efficient*

human cell infection and bypass of preexisting adenovirus immunity. J Virol, 2003. 77(15): p. 8263-71.

24. Barouch, D.H., M.G. Pau, J.H. Custers, W. Koudstaal, S. Kostense, et al., *Immunogenicity of recombinant adenovirus serotype 35 vaccine in the presence of pre-existing anti-Ad5 immunity.* J Immunol, 2004. 172(10): p. 6290-7.

25. Roberts, D.M., A. Nanda, M.J. Havenga, P. Abbink, D.M. Lynch, et al., *Hexon-chimaeric adenovirus serotype 5 vectors circumvent pre-existing anti-vector immunity.* Nature, 2006. 441(7090): p. 239-43.

26. Kong, W.P., Y. Huang, Z.Y. Yang, B.K. Chakrabarti, Z. Moodie, et al., *Immunogenicity of multiple gene and clade human immunodeficiency virus type 1 DNA vaccines.* J Virol, 2003. 77(23): p. 12764-72.

27. Barouch, D.H., Z.Y. Yang, W.P. Kong, B. Korioth-Schmitz, S.M. Sumida, et al., *A human T-cell leukemia virus type 1 regulatory element enhances the immunogenicity of human immunodeficiency virus type 1 DNA vaccines in mice and nonhuman primates.* J Virol, 2005. 79(14): p. 8828-34.

28. Helms, T., B.O. Boehm, R.J. Asaad, R.P. Trezza, P.V. Lehmann, et al., *Direct visualization of cytokine-producing recall antigen-specific CD4 memory T cells in healthy individuals and HIV patients.* J Immunol, 2000. 164(7): p. 3723-32.

29. Betts, M.R., J.P. Casazza, and R.A. Koup, *Monitoring HIV-specific CD8+ T cell responses by intracellular cytokine production.* Immunol Lett, 2001. 79(1-2): p. 117-25.

30. Malenbaum, S.E., D. Yang, and C. Cheng-Mayer, *Evidence for similar recognition of the conserved neutralization epitopes of human immunodeficiency virus type 1 envelope gp120 in humans and macaques.* J Virol, 2001. 75(19): p. 9287-96.

31. Mascola, J.R., P. D'Souza, P. Gilbert, B.H. Hahn, N.L. Haigwood, et al., *Recommendations for the design and use of standard virus panels to assess neutralizing antibody responses elicited by candidate human immunodeficiency virus type 1 vaccines.* J Virol, 2005. 79(16): p. 10103-7.

32. Li, M., F. Gao, J.R. Mascola, L. Stamatatos, V.R. Polonis, et al., *Human immunodeficiency virus type 1 env clones from acute and early subtype B infections for standardized assessments of vaccine-elicited neutralizing antibodies.* J Virol, 2005. 79(16): p. 10108-25.

33. Sprangers, M.C., W. Lakhai, W. Koudstaal, M. Verhoeven, B.F. Koel, et al., *Quantifying adenovirus-neutralizing antibodies by luciferase transgene detection: addressing preexisting immunity to vaccine and gene therapy vectors.* J Clin Microbiol, 2003. 41(11): p. 5046-52.

34. Chakrabarti, B.K., W.P. Kong, B.Y. Wu, Z.Y. Yang, J. Friborg, et al., *Modifications of human immunodeficiency virus envelope glycoprotein enhance immunogenicity for genetic immunization.* J Virol, 2002. 76(11): p. 5357-68.

35. Butman, B.T., P.L. Gomez, R. Sheets, J. Gall, D. Brough, et al., *Manufacture and testing of a multi-clade adenoviral vector-based candidate vaccine against human immunodeficiency virus.* BioProcessing Journal, 2006. 5(2): p. 15-9.

36. Seth, P., *Adenoviruses : basic biology to gene therapy*. Medical intelligence unit. 1999, Austin, TX: R.G. Landes Co.

37. Butman, B.T., A. Lizonova, D.E. Brough, J.M. Sowers, R. Sheets, et al., *Comprehensive characterization of the 293-ORF6 cell line.* Dev Biol (Basel), 2006. 123: p. 225-33; discussion 65-6.

38. Malaeb, B.S., T.A. Gardner, V. Margulis, L. Yang, J.Y. Gillenwater, et al., *Elevated activated partial thromboplastin time during administration of first-generation adenoviral vectors for gene therapy for prostate cancer: identification of lupus anticoagulants.* Urology, 2005. 66(4): p. 830-4.

39. Letvin, N.L., J.R. Mascola, Y. Sun, D.A. Gorgone, A.P. Buzby, et al., *Preserved CD4+ central memory T cells and survival in vaccinated SIV-challenged monkeys.* Science, 2006. 312(5779): p. 1530-3.

APPENDIX I

STUDY INFORMED CONSENT FORM

INSTITUTE: Vaccine Research Center, National Institute of Allergy and Infectious Diseases

STUDY NUMBER: 07-I-0167 PRINCIPAL INVESTIGATOR: Barney S. Graham, M.D., Ph.D.

STUDY TITLE: VRC 012: A Phase I Clinical Trial of the Safety and Immunogenicity of an HIV-1 Adenoviral Vector Serotype 35 Vaccine, VRC-HIVADV027-00-VP (rAd35-EnvA): Dose Escalation as a Single Agent and Prime-Boost Schedules with an HIV-1 Adenoviral Vector Serotype 5 Vaccine, VRC-HIVADV038-00-VP (rAd5-EnvA), in Uninfected Adults

Latest IRB Review:
Latest Amendment Approved:

Part II Study Consent: Version 5.0

INTRODUCTION

We invite you to take part in a research study at the National Institutes of Health (NIH).

First, we want you to know that:

> Taking part in NIH research is entirely voluntary.
>
> You may choose not to take part, or you may withdraw from the study at any time. In either case, you will not lose any benefits to which you are otherwise entitled. However, to receive care at the NIH, you must be taking part in a study or be under evaluation for study participation.
>
> You may receive no benefit from taking part. The research may give us knowledge that may help people in the future.

Before you decide to take part, please take as much time as you need to ask any questions and discuss this study with anyone at NIH, or with family, friends or your personal physician or other health professional. You will be informed of anything new during this study that might cause you to change your mind about staying in the study.

PURPOSE OF THE STUDY

The main purpose of this study is to see if two new experimental HIV vaccines are safe and whether they cause any side effects in healthy adult volunteers. "Experimental" means that the study vaccines have not been approved by the Food and Drug Administration (FDA) for treating or preventing HIV infection. The FDA allows them to be used in research studies only.

You are eligible to participate in this study because you:

- have completed the screening process,
- have completed an assessment of understanding,
- are HIV-negative,
- are between 18 and 50 years old,

- are available for the time needed to complete the study,
- do not have any significant medical problems,
- are willing to donate blood samples for future research,
- are assessed as being at low risk of HIV exposure and infection,
- are willing to comply with HIV risk reduction practices and counseling during the study,
- are willing to be contacted annually after the last study visit for follow-up until the 5th year after your enrollment.

Thirty-five (35) people will participate in this study at the NIH Clinical Center in Bethesda, Maryland. While on the study, you will be checked for vaccine side effects. You will be treated at the National Institutes of Health if any side effects occur.

Potential Risk of HIV Vaccine:

You should know that receiving an experimental HIV vaccine could increase (rather than decrease or not change) your risk of getting HIV, if you are later exposed to HIV (for example through sex or drug use). This may have been the case for certain participants in the Step Study. This study tested an HIV vaccine made by Merck, Inc. This vaccine included a common cold virus called adenovirus (type 5). This adenovirus was changed so it could not infect people. It was used to carry bits of HIV genes into the body.

The Step Study enrolled 1850 men and 1150 women at increased risk of HIV infection. Half got the Merck rAd5 vaccine and half got injections without the vaccine (placebo). Everyone got counseling about how to lower their risk of HIV infection. The study was stopped early for two reasons. The vaccine did not prevent HIV infection and it did not lower the amount of HIV virus in the blood. When the study was stopped, 82 men had gotten HIV infections; there were 49 HIV infections in the vaccine group and 33 in the placebo group. There was 1 women in the study who had HIV infection. Each case was reviewed carefully. The vaccine itself did **NOT** give anyone HIV.

In the Step Study, some men who got vaccine were more likely to get HIV. Two factors seemed to increase the risk of HIV infection in the vaccinated men compared to men who got placebo:

- having had an adenovirus (type 5) infection in the past before joining the study. (This is found by looking for "Ad5 antibody" in a blood test).
- having a foreskin on the penis (being uncircumcised).

Men with both of these factors had the highest risk of HIV infection. We do not yet know why.

One (1) woman had HIV in the Step Study at the time it was stopped. A study of the Merck rAd5 vaccine in South Africa (called "Phambili") was stopped early. There were some HIV infections in women who got vaccine and in women who got placebo. The numbers are small, so Phambili cannot be analyzed in the same way as the Step Study. It is possible that vaccinated women could be at increased risk of HIV infection.

The VRC rAd5 vaccine is similar in some ways and different in other ways from the Merck rAd5 vaccine.

If you are enrolling into VRC 012, Part I, you must have no antibody to Ad35 when tested during screening. If you are enrolling into VRC 012, Part II you must have no antibody to either Ad5 orAd35 when tested during screening. **It is important for you to continue to avoid behaviors associated with the risk of getting HIV infection**.

STUDY VACCINES

Vaccines are substances used to try to create an immune response (the body's natural defenses) to prevent or resist an infection. There is no live HIV virus in the study vaccines. The vaccine itself cannot cause you to become infected with HIV. You must be exposed to the HIV virus (for example through sex with an HIV-infected person) to become infected with HIV.

The two experimental HIV vaccines in this study are known as VRC-HIVADV027-00-VP and VRC-HIVADV038-00-VP. In this consent document, we call the first one the "rAd35-EnvA vaccine" and the second one the "rAd5-EnvA vaccine."

Adenovirus type 5 is a common virus that causes upper respiratory infections (such as the common cold), eye infection (conjunctivitis), urine infection or diarrhea. Adenovirus type 35 also causes upper respiratory infections, but is less common in the United States. The rAd35-EnvA vaccine and the rAd5-EnvA are modified adenoviruses that will carry DNA into cells in the body. The vaccines code for parts of the HIV proteins called Env. The manufactured DNA has been packaged in an adenovirus shell that is missing some of the usual adenovirus genes. It cannot reproduce in a human body. You cannot infect someone else with the study vaccine adenovirus.

The study vaccines are manufactured by packaging DNA into an adenovirus shell, similarly to the Merck vaccine. The study vaccines code for a different HIV protein than was in the Merck vaccine.

The NIH, including some members of the Vaccine Research Center scientific staff, developed the investigational vaccines being used in this research study. The results of this study could play a role in whether the FDA will approve the vaccines for sale at some time in the future. If approved, the future sale of the vaccines could lead to payments to the NIH and to some NIH/VRC scientists. By U.S. law, government scientists are required to receive such payments for their inventions. You will not receive money or other compensation should this occur. Please discuss with your study doctor any questions you may have about these issues.

STUDY PROCEDURES

The study has two Parts. Part I of the study enrolled 15 people into 3 different dose groups to receive one injection of the rAd35-EnvA vaccine. The dosages tested were 10^9 particle units, 10^{10} particle units and 10^{11} particle units.

You will be in Part II of the study. In Part II, 20 people will be enrolled. Each person in Part II will be randomly assigned (like flipping a coin) to vaccination schedules that both include injection of the rAd35-EnvA and the rAd5-EnvA vaccines. The schedules vary in the order that the two vaccine types are given. The first 10 people in Part II will get both injections at a dose of

10^{10} particle units. The last 10 people in Part II will get the rAd5-EnvA injection at 10^{10} particle units and the rAd35-EnvA injection at 10^{11} particle units.

**<u>PART II</u> Injection Schedule**	**Day 0<br>PRIME**	**Week 12<br>BOOST**
Group 4A = 5 people	rAd35-EnvA (10^{10} particle units)	rAd5-EnvA (10^{10} particle units)
Group 4B = 5 people	rAd5-EnvA (10^{10} particle units)	rAd35-EnvA (10^{10} particle units)
Group 4C = 5 people	rAd35-EnvA (10^{11} particle units)	rAd5-EnvA (10^{10} particle units)
Group 4D = 5 people	rAd5-EnvA (10^{10} particle units)	rAd35-EnvA (10^{11} particle units)
**TOTAL: 20 people**		

You and the study staff will know what dosage of each vaccine you will get, but will not know the order in which the vaccines are given. You will have an equal chance of being assigned to either order of administration. When the last person in Group 4 has completed a period of follow-up to the second injection, you may learn the order in which you received the study vaccines.

In the study all injections will be given in the upper arm. You will get an injection on the day you enroll in the study. This is called Day 0. The clinic staff will observe you for at least 30 minutes after an injection. You will be asked to keep track of your symptoms at home for 5 days after an injection.

After any injection, you must come to the clinic to be seen if you have a rash, hives, fever of 101.6˚ F or higher, or a lot of difficulty in daily activities (such as not being able to go to work or take care of yourself). You will also need to come to the clinic for any problem which the nurse or doctor thinks should be checked by exam or blood tests or urine tests. It is very important that you follow the instructions given to you by the study nurse.

You will have about 9 clinic visits over 52 weeks. The vaccination visits will each take about 4 hours to complete. Other clinic visits will usually take about 1 hour. At each visit, you will be checked for any health changes or problems. You will be asked how you are feeling and if you have taken any medications or supplements, including those that are not prescribed by a doctor. If it is not an emergency, call a study nurse or doctor before starting new medicines or getting other vaccines or shots of any type.

Urine samples will be collected and blood will be drawn at some study visits to check on your health. You will be told right away if any of your test results show a health problem. Some blood samples will be used to study your immune response to the vaccine.

The amount of blood drawn will vary from about 1 tablespoon (15 mL) to about 9 tablespoons (135 mL), depending on the visit. No more than a total of about two cups (450 mL) will be drawn over any six-week period during the study.

You will be tested for HIV several times and asked questions about your sexual behavior and drug use. Throughout the study you will be counseled on how to lower your risk of getting HIV. You will be asked about any social effects you may have experienced from your participation in this study.

The vaccines used in this study cannot cause HIV infection. However, if you become infected with HIV during this study for other reasons, you will not receive any additional study injections. You will be referred for medical care. Medical treatment for HIV infection is not part of this study. You will be asked to continue with study follow-up visits.

If you are in Part II, you will be contacted at least once per year for 4 years after the last study clinic visit. You will have a choice of completing the annual contact as a clinic visit or contact by telephone, e-mail or mail to answer questions about your health. If you come to the clinic you will be asked to allow blood samples to be collected for research and HIV testing. About seven tablespoons of blood (110 mL) will be collected.

MONITORING OF THE STUDY

This study will be monitored by a group of physicians and scientists at the Clinical Center. This group will review the information from the study and will pay close attention to any serious side effects. If serious side effects occur, further injections may be delayed or canceled.

HIV TESTING

We will test your blood for HIV, the virus that causes AIDS. If you have HIV, we will explain what it means for you. If you live in Maryland, the NIH Clinical Center will report your name and HIV results to the Maryland Department of Health and Mental Hygiene. If you have any questions regarding the HIV testing, you are encouraged to discuss them with the study nurse or doctor, or you may call a Clinical Center HIV counselor at 301-496-2381.

GENETIC TESTING

In the future, genetic research tests to help understand how vaccines work may be done on your DNA using stored samples. In vaccine research some genetic tests are done to see if different types of immune response to a vaccine seem to be related to genetic differences in people. Genetic tests done in a research lab using your stored samples will **not** be in your medical record and will **not** have your name on the sample.

HLA type is a genetic test ordered through the NIH Clinical Center medical laboratory. HLA type results will be in your medical record at the NIH Clinical Center. People with certain HLA types might be more likely to develop certain diseases. Simply having those HLA types doesn't mean they will develop those diseases. It is our policy not to discuss your HLA results unless it

has direct medical or reproductive implications for you or your family. The results of these tests are not used to make health care decisions.

STORED SAMPLES

Some of the blood samples collected from you will be stored for future research to learn more about HIV and HIV vaccines, the immune system, and/or other medical conditions. The results from the research done with your stored samples will not be given to your health care provider and will not be put in your medical record.

Labeling of Stored Samples

Your stored samples will be labeled by a code (such as a number) that only the study team can link to you. Any identifying information about you will be kept confidential to the extent permitted by law.

Risks from Stored Samples

The greatest risk is the unplanned release of information from your medical records. The chance that this information will be given to an unauthorized person without your permission is very small. Possible problems with the unplanned release of information include discrimination when applying for insurance and employment. Similar problems may occur if you disclose information yourself or agree to have your medical records released.

Future Studies

In the future, other investigators (at NIH or outside of NIH) may wish to study your stored samples. When the study team shares your materials, they may share it with no identifying information or with a code. Some information about you, such as your gender, age, health history, or ethnicity may also be shared with other investigators. Any future research studies using your samples will be reviewed by the investigator's Institutional Review Board (IRB), a special committee that oversees medical research studies to protect the rights and welfare of human volunteers.

Your stored materials will be used only for research and will not be sold. The research done with your materials may be used to develop new products in the future but you will not receive payment for such products.

Making your Choice

If you agree to participate in this study, you agree to let us store your samples for future research. No matter what you decide, you may still participate in other studies at NIH. However, refusal to let us store your samples means you are not eligible to be in this specific study. Even if you agree now to let us store your samples, you can change your mind later. If you do, please contact us and say that you do not want us to use your stored samples for future research.

POSSIBLE STUDY RISKS

Vaccine Risks

It is possible to have one or more of the following side effects: fever, chills, rash, aches and pains, nausea, headache, dizziness, or fatigue. As with all vaccines or drugs, you could have an immediate allergic reaction, including a rash, hives, or even difficulty breathing. Allergic reactions can be life threatening; therefore, the clinic staff will watch you for at least 30 minutes after each immunization and provide any needed treatment. There may be other side effects, even serious ones that we don't know about yet. Therefore, it is important that you report any side effects to the clinic staff as soon as they occur.

Investigational adenoviral vector vaccines have been given to hundreds of people in several different studies. Some people have a flu-like condition with fever, headache, muscle aches, tired feeling and chills. It starts about 12-16 hours after vaccination and lasts a few hours. A few people have had nausea. Some people have injection site pain or discomfort in the first few days after a vaccination. The flu-like symptoms and injection site pain or discomfort may be treated with an over-the-counter medicine for pain and fever.

<u>Adenovirus Antibodies</u>: You may develop antibodies to the adenovirus type in the injection or injections you receive in this study. It is possible you would not be able to receive (or have a reduced response to) future products that used these same types of adenoviral vector. Currently there are no products approved by the FDA that use an adenoviral vector. There are other experimental products that use an adenoviral vector.

Other Risks of Being in an HIV Vaccine Study

Getting the experimental vaccines in this study may mean that you cannot be in other experimental HIV vaccine studies later. It is also possible that receiving the experimental HIV vaccine may alter your response to future HIV vaccines and may make them either more or less effective. If you are exposed to HIV through sex or drug use after receiving the study injection, your risk of becoming HIV infected is unknown. Please do not do anything that might expose you to HIV.

You should be aware that some people who received experimental HIV vaccines in the past became infected with HIV through sex or drug use. We know that HIV infection and AIDS can develop even in a person who has received a test vaccine if he/she is exposed to HIV. If you are exposed to HIV through sex or drug use after receiving the study vaccines, your risk of becoming infected with HIV and developing AIDS is unknown. Please inform the VRC Clinic any time you think that you may have been exposed to HIV. If you do get infected, we do not know what effect the study vaccines may have on the disease. The time that it takes for you to become sick from HIV/AIDS may be the same, longer or shorter than usual. You will be educated and counseled about HIV exposure often during the study. If you have questions, please ask the clinic staff.

Risk of Developing a "False" Positive HIV Test

At the time you enroll in the study you must have a negative HIV antibody test. An HIV antibody test (called an ELISA or Western Blot) is the usual way to test for HIV infection. <u>After the study vaccinations, it is very likely that you will test positive for HIV antibody from the study vaccines.</u> However, it will be possible, by using tests for the presence of HIV virus (called PCR or viral load testing), to show when a positive result on the HIV antibody test is NOT

because of an HIV infection. A positive antibody test in a person who is not HIV infected is called a "false positive" test. If you do have a false positive HIV antibody test caused by the experimental vaccine, it is unknown how long the test will be positive. You cannot pass antibodies to your partner. If you have a false positive antibody test at the end of the study you are encouraged to be retested at the VRC Clinic at least once per year or before taking the test for insurance, travel or other purposes. You may continue to have HIV testing at the VRC Clinic for five years so that you can find out if it changes back to negative. There are social risks from having your HIV test appear positive. For this reason you are advised to have all HIV testing done at the VRC Clinic while you are participating in the study and for up to five years afterwards. Counseling about HIV tests, including social problems related to false positive results, is offered at all clinic visits. You may also call the clinic at other times if you have questions or concerns.

Any time you have a positive HIV antibody test in the future, you must also have an HIV "viral load" (PCR) test. Otherwise, you and others will not know if the positive HIV antibody test is from the study vaccines or from HIV infection.

You will not be able to donate blood while you are participating in the study and for at least one year after the last study injection. You may not be able to donate blood ever again if you have a false positive test when you try to donate blood. Please be sure you have a negative HIV antibody test before trying to donate blood.

If you have a false positive antibody response on HIV tests, you may also have difficulties with:

- Health insurance
- Life Insurance
- Medical or dental care
- Travel to other countries or immigration
- Employment
- Education
- Housing
- Military services or other government agencies
- Personal relationships

If you have problems like these, the staff at the clinic will try to help you work through them. If your blood tests look HIV positive because of study vaccinations you will be offered a letter that shows you joined this study and that describes the antibody response caused by the vaccine. Even so, this letter or other help offered by the VRC Clinic may not solve a social problem caused by a false positive HIV antibody test.

It is also possible that others may learn that you are taking part in this study and assume that you are at risk of HIV infection because of sexual behavior or drug use. This may result in some people treating you differently.

Risks from Pregnancy

We do not know the possible effects of the study vaccines on the fetus or nursing infant. Women who are able to have children must agree to practice adequate birth control beginning at least 21 days prior to receiving the first injection until 12 weeks after the last study injection received. Birth control include: condoms, male or female, with or without a spermicide; diaphragm or cervical cap with spermicide; intrauterine device; birth control pills or patch, Depo-Provera, other prescription methods or a male partner who had a vasectomy. Women must have a negative pregnancy test before each study injection. You must notify the clinic staff right away if you become pregnant during this study or think that you might be pregnant. If you become pregnant, you will not get any more injections. However, you will be asked to continue with study follow-up visits as well as to report the outcome of the pregnancy.

POSSIBLE BENEFITS

This study will not provide any direct benefit to you. However, you and others may benefit in the future from the information that will be learned from the study.

COSTS TO YOU FOR YOUR PARTICIPATION

You do not have to pay for the vaccines, research clinic visits, examinations or laboratory tests that are part of this study. All medical costs outside this study will be paid by you or your health insurance carrier (if you have insurance).

PAYMENT TO YOU FOR YOUR PARTICIPATION

You will be compensated $175 for each visit with blood drawing but no injection. You will be compensated $275 for each visit that includes an injection and blood drawing. In Part II, the approximate total compensation will be about $1,775 for participants who complete the study. Actual compensation is based on the number of study visits you attend and number of study injections you receive. You will be paid throughout the study by checks, which will be mailed to you after each reimbursable visit. You will be compensated $175 for each of the scheduled long-term follow-up contacts that is completed as a clinic visit with blood drawing.

REASONS FOR STOPPING STUDY VACCINATIONS WITHOUT YOUR CONSENT

Study vaccination may be stopped without your consent for several different reasons, including:

- You don't keep appointments or follow study procedures
- The study sponsor or study doctor decides to stop or cancel the study
- The Institutional Review Board (IRB) or the FDA decide that the study should be stopped
- You get a serious illness
- You receive a medication that affects your immune system
- You have a serious side effect thought to be caused by study vaccine.
- You become pregnant

If you agree to take part in this study, it is important for you to keep all your appointments. We will ask you to continue with follow-up visits to check on your health and immune system response even if the vaccinations are stopped. If you want to stop getting vaccinations, you may

stop at any time. You may also stop coming to clinic visits, but we recommend that you continue with the check-ups because continuing to attend the study visits is for your benefit. If you choose to stop vaccinations, we will not pressure you to continue vaccinations during the health check-up visits.

ALTERNATIVES

You may choose to not participate in any HIV vaccine study. You may be eligible for other studies, including those testing other experimental HIV vaccines. Your study doctor can discuss the risk and benefits of alternative studies.

COMMUNITY RESOURCES

You may also be interested in contacting local volunteer panels of individuals from the general public that were organized to assist and advise AIDS vaccine trials in the metropolitan Washington DC area, the Capital Area Vaccine Effort (CAVE) and the Community Advisory Board (CAB). Information is available at the Internet site http://www.aidsvaccine.org.

OTHER PERTINENT INFORMATION

1. Confidentiality. When results of an NIH research study are reported in medical journals or at scientific meetings, the people who take part are not named and identified. In most cases, the NIH will not release any information about your research involvement without your written permission. However, if you sign a release of information form, for example, for an insurance company, the NIH will give the insurance company information from your medical record. This information might affect (either favorably or unfavorably) the willingness of the insurance company to sell you insurance.

The Federal Privacy Act protects the confidentiality of your NIH medical records. However, you should know that the Act allows release of some information from your medical record without your permission, for example, if it is required by the Food and Drug Administration (FDA), members of Congress, law enforcement officials, or other authorized people.

2. Policy Regarding Research-Related Injuries. The Clinical Center will provide short-term medical care for any injury resulting from your participation in research here. In general, no long-term medical care or financial compensation for research-related injuries will be provided by the National Institutes of Health, the Clinical Center, or the Federal Government. However, you have the right to pursue legal remedy if you believe that your injury justifies such action.

3. Payments. The amount of payment to research volunteers is guided by the National Institutes of Health policies.

4. Problems or Questions. If you have any problems or questions about this study or about any research-related injury, contact the Principal Investigator, Barney S. Graham, M.D., Ph.D. at 301-594-8468 or Study Coordinator Laura Novik, RN at 301-451-8715 or 1-800-NIH-BEEP ext 14881.

If you have any questions about your rights as a research subject, you may call the Clinical Center Patient Representative at 301-496-2626.

5. Consent Document. Please keep a copy of this document in case you want to read it again.

COMPLETE APPROPRIATE ITEM(S) BELOW:

A. Adult Study Participant's Consent

I have read the explanation about this study and have been given the opportunity to discuss it and to ask questions. I hereby consent to take part in this study.

Time

Signature of Adult Participant/Legal Representative Date

THIS CONSENT DOCUMENT HAS BEEN APPROVED FOR USE FROM XXXXXX THROUGH XXXXXX.

Time: ________ Time: ________

Signature of Investigator/Person Obtaining Consent Date Signature of Witness Date

APPENDIX II
CONTACT INFORMATION

Contact Information Page Removed

APPENDIX III

SCHEDULE OF EVALUATIONS

PART I AND PART II SCHEDULES

**VRC 000**			**VRC 012: PART I**					
[1] Visit		01	02	02B	02C	03	04	05
Week of Study			Wk 0	W 1	W 2	W 4	W 12	W 24
Day of Study			D 0	D 2	D 14	D 28	D 84	D 168
**Clinical**	**Tube**							
VRC 000 Screening consent		X						
VRC 012 Assessment of Understanding & Consent			X					
[2] Physical exam		X	[X]		[X]	[X]	[X]	[X]
Complete or interim medical history, vital signs, weight		X	X		X	X	X	X
Lymph node assessment		X	X		X	[X]	[X]	[X]
Vaccination			X					
5-day Diary Card			start					
[3] Phone evaluation (clinic visit if needed)				X				
Counsel on HIV throughout; pregnancy through 12 wks post-injection		X	X		X	X	X	X
Social Impact Assessment								X
Urinalysis		X	X		X	X		
CBC, differential, platelets	Lav.	3	3		3	3	3	3
PT, aPTT	Blue	5						
[4] Pregnancy test: urine or serum		X	X					X
Chemistry Panel (Chem 20)	SST	4						
ALT & Creatinine	SST		4		4	4	4	4
HBsAg, Anti-HCV, HCV PCR	SST	8						
HLA class I, II antigens	ACD				20			
ELISA/Western Blot	SST	8	8			8		8
HIV PCR	Lav	3	3			3		3
[5]T cell Subsets	Lav		3			[3]		[3]
Anti-dsDNA, RPR, Immunoglobulins	SST	4						
**Research**								
Adenovirus Serology	SST	4				4		
HIV-specific antibody	SST		16			16		
ICS and ELISpot; PBMC & Plasma for Storage	EDTA	60	80			80	80	80
Serum Storage	SST	20	16			12	16	16
**Daily Volume (mL)**		**119**	**133**		**27**	**130**	**103**	**114**
**Cumulative Volume (mL)**		**119**	**252**		**279**	**409**	**512**	**626**

[1] Visit 01 (under VRC 000) screening may be completed over 1 or more visits. Day 0 (day of enrollment) pregnancy test is used for eligibility. Day 0 evaluations prior to vaccination are the baseline for subsequent safety assessments. "A" visits (not shown) are evaluations (vital signs & injection site assessment) completed between 30-45 minutes post vaccination. Visit 02B is Day 2 ±1 day. Other visits and permitted windows are as follows: 02C (Week 2 ±3 days), 03 (Week 4 ±3 days), 04 (Week 12 ± 7 days) and 05 (Week 24 ± 14 days). Subjects with vaccine-induced HIV antibody who choose to do so, may return for HIV testing after study completion.

[2] Screening (Visit 01) includes a physical exam, at other visits a targeted physical exam is done if indicated (shown as [X]) by interim history or lab results.

[3]A clinic visit is required within 24 hrs if there is rash, urticaria, fever ≥ 38.7°C that does not resolve within 24 hours, or significant impairment in ADL.

[4] Negative pregnancy results must be confirmed prior to vaccination.

[5]T cell subsets are done at Day 0. After that they are done only if there is a positive HIV PCR at the visits shown by [3].

VRC 000			VRC 012 PART II											
[1] Visit		01	02	02B	02C	03	04	04B	04C	05	06	07	08	09 to 12
Week of Study			Wk 0	W 1	W 2	W 4	W 12	W 13	W14	W16	W 24	W 36	W 52	Yrs 2 to 5
[1]Day of Study			D 0	D 2	D 14	D 28	D 84	D86	W 98	D 112	D 168	D 252	D 364	
**Clinical**	**Tube**													
VRC 000 Screening consent		X												
VRC 012 Assessment of Understanding & Consent			X											
[2] Physical exam		X	[X]		[X]	[X]	[X]		[X]	[X]	[X]	[X]	[X]	
Medical history; vital signs, weight through wk 52; Specified long-term follow-up information years 2-5		X	X		X	X	X		X	X	X	X	X	X
Lymph node assessment		X	X		X	[X]	X		X	[X]	[X]	[X]	[X]	
Vaccination			X				X							
5-day Diary Card			start				start							
[3] Phone evaluation (clinic visit if needed)				X				X						
Counsel HIV throughout; pregnancy through week 36		X	X		X	X	X		X	X	X	X		
Social Impact Assessment													X	
Urinalysis		X	X		X	X	X		X	X				
CBC, differential, platelets	Lav.	3	3		3	3	3		3	3	3	3	3	
PT, aPTT	Blue	5	5		5	5	5		5	5				
[4] Pregnancy test: urine or serum		X	X				X						X	
Chemistry Panel (Chem 20)	SST	4												
ALT & Creatinine	SST		4		4	4	4		4	4	4	4	4	
HBsAg, Anti-HCV, HCV PCR	SST	8												
HLA class I, II antigens	ACD				20									
ELISA/Western Blot	SST	8	8			8	8			8	8	8	8	[8]
HIV PCR	Lav	3	3			3	3			3	3	3	3	[3]
[5]T cell Subsets	Lav		3			[3]	[3]			[3]	[3]	[3]	[3]	[3]
Anti-dsDNA, RPR, Immunoglobulins	SST	4												
**Research**														
Adenovirus Serology	SST	4				4	4			4			4	
HIV-specific antibody	SST		16			16	16			16				
ICS and ELISpot; PBMC & Plasma for Storage	EDTA	60	80			80	80			80	80	80	80	[80]
Serum Storage	SST	20	16			12	12			12	16	16	16	[16]
**Daily Volume (mL)**		**119**	**138**		**32**	**135**	**135**		**12**	**135**	**114**	**114**	**118**	**[110]**
**Cumulative Volume (mL)**		**119**	**257**		**289**	**424**	**559**		**571**	**706**	**820**	**934**	**1052**	

[1]Visit 01 (under VRC 000) screening may be completed over 1 or more visits. Day 0 (day of enrollment) pregnancy test is used for eligibility. Day 0 evaluations prior to vaccination are the baseline for subsequent safety assessments. "A" visits (not shown) are evaluations (vital signs & injection site assessment) completed between 30-45 minutes post vaccination. "B" visits are phone assessments at 2 (±1) days after vaccination. "C" visits are 14 (±3) days after vaccination. Other visits and permitted windows are as follows: 03 is Week 4 ±3 days, 04 (boost vaccination) is at Week 12 (-7 to +21 days), 05 (Week 16 ± 7 days), 06 (Week 24 ± 7 days), 07 (Week 36 ± 14 days) and 08 (Week 52 ± 14 days). Attempt to complete Visit 09 (Week 104), Visit 10 (Week 156), Visit 11 (Week 208) and Visit 12 (Week 360) long-term follow-up contacts with a ± 28 day window.

[2]Screening visit (01) includes a physical exam; at other visits a targeted physical exam (indicated by [X]) is done if indicated by interim history or lab results.

[3]A clinic visit is required within 24 hrs if there is rash, urticaria, fever ≥ 38.7°C that does not resolve within 24 hours, or significant impairment in ADL.

[4]Negative pregnancy results must be confirmed prior to vaccination

[5] T cell subsets are done at Day 0. After that they are done only if there is a positive HIV PCR at the visits shown by [3].

APPENDIX IV
TABLE FOR GRADING SEVERITY OF ADVERSE EVENTS

The Division of AIDS Table for Grading the Severity of Adult and Pediatric Adverse Events, Version 1.0, Dec 2004

The table for Grading Severity of Adverse Events in this protocol is based upon "The Division of AIDS Table for Grading the Severity of Adult and Pediatric Adverse Events, Version 1.0, Dec 2004" but this table has been amended for PTT and seizure severity grading criteria in keeping with an FDA request for a different VRC vaccine study. The VRC has found the revised PTT severity definition to be appropriate for healthy volunteers. The seizure criterion was also amended so that there would be only one amended table in use within the VRC clinic.

Division of AIDS Table for Grading the Severity of ADULT AND PEDIATRIC Adverse Events
Publish Date: December, 2004
(With PTT and seizure grading criteria amended for VRC 012)

Quick Reference

The Division of AIDS Table for Grading the Severity of Adult and Pediatric Adverse Events (“DAIDS AE grading table”) is a descriptive terminology which can be utilized for Adverse Event (AE) reporting. A grading (severity) scale is provided for each AE term.

General Instructions

Estimating Severity Grade

If the need arises to grade a clinical AE that is not identified in the DAIDS AE grading table, use the category “Estimating Severity Grade” located at the top of Page 3. For AEs that are not listed in the table but will be collected systematically for a study/trial, protocol teams are highly encouraged to define study-specific severity scales within the protocol or an appendix to the protocol. (Please see “Template Wording for the Expedited Adverse Event Reporting Section of DAIDS-sponsored Protocols”.) This is particularly important for laboratory values because the “Estimating Severity Grade” category only applies to clinical symptoms.

Grading Adult and Pediatric AEs

The DAIDS AE grading table includes parameters for grading both Adult and Pediatric AEs. When a single set of parameters is not appropriate for grading specific types of AEs for both Adult and Pediatric populations, separate sets of parameters for Adult and/or Pediatric populations (with specified respective age ranges) are given in the table. If there is no distinction in the table between Adult and Pediatric values for a type of AE, then the single set of parameters listed is to be used for grading the severity of both Adult and Pediatric events of that type.

Determining Severity Grade

If the severity of an AE could fall under either one of two grades (e.g., the severity of an AE could be either Grade 2 or Grade 3), select the higher of the two grades for the AE.

Definitions

Basic Self-care Functions	Adult<br>Activities such as bathing, dressing, toileting, transfer/movement, continence, and feeding.<br>Young Children<br>Activities that are age and culturally appropriate (e.g., feeding self with culturally appropriate eating implement).
LLN	Lower limit of normal
Medical Intervention	Use of pharmacologic or biologic agent(s) for treatment of an AE.
NA	Not Applicable

Operative Intervention	Surgical OR other invasive mechanical procedures.
ULN	Upper limit of normal
Usual Social & Functional Activities	Adult<br>Adaptive tasks and desirable activities, such as going to work, shopping, cooking, use of transportation, pursuing a hobby, etc.<br>Young Children<br>Activities that are age and culturally appropriate (e.g., social interactions, play activities, learning tasks, etc.).

Contents

Clinical — Page

Estimating Severity Grade.................... 3
Systemic... 3
Infection... 4
Injection Site Reactions........................ 4
Skin – Dermatological 5
Cardiovascular 5
Gastrointestinal 7
Neurologic 9
Respiratory... 12
Musculoskeletal.................................. 12
Genitourinary...................................... 13
Ocular/Visual...................................... 14
Endocrine/Metabolic........................... 14

Laboratory — Page

Hematology..16
Chemistries..17
Urinalysis 20

DIVISION OF AIDS TABLE FOR GRADING THE SEVERITY OF ADULT AND PEDIATRIC ADVERSE EVENTS
PUBLISH DATE: DECEMBER, 2004
(With PTT and seizure grading criteria amended for VRC 012)

CLINICAL				
**PARAMETER**	**GRADE 1 MILD**	**GRADE 2 MODERATE**	**GRADE 3 SEVERE**	**GRADE 4 POTENTIALLY LIFE-THREATENING**
**ESTIMATING SEVERITY GRADE**				
Clinical adverse event NOT identified elsewhere in this DAIDS AE grading table	Symptoms causing no or minimal interference with usual social & functional activities	Symptoms causing greater than minimal interference with usual social & functional activities	Symptoms causing inability to perform usual social & functional activities	Symptoms causing inability to perform basic self-care functions OR Medical or operative intervention indicated to prevent permanent impairment, persistent disability, or death
**SYSTEMIC**				
Acute systemic allergic reaction	Localized urticaria (wheals) with no medical intervention indicated	Localized urticaria with medical intervention indicated OR Mild angioedema with no medical intervention indicated	Generalized urticaria OR Angioedema with medical intervention indicated OR Symptomatic mild bronchospasm	Acute anaphylaxis OR Life-threatening bronchospasm OR laryngeal edema
Chills	Symptoms causing no or minimal interference with usual social & functional activities	Symptoms causing greater than minimal interference with usual social & functional activities	Symptoms causing inability to perform usual social & functional activities	NA
Fatigue<br>Malaise	Symptoms causing no or minimal interference with usual social & functional activities	Symptoms causing greater than minimal interference with usual social & functional activities	Symptoms causing inability to perform usual social & functional activities	Incapacitating fatigue/ malaise symptoms causing inability to perform basic self-care functions
Fever (nonaxillary)	37.7 – 38.6°C	38.7 – 39.3°C	39.4 – 40.5°C	> 40.5°C
Pain (indicate body site)<br>DO NOT use for pain due to injection (See Injection Site Reactions: Injection site pain)<br>See also Headache, Arthralgia, and Myalgia	Pain causing no or minimal interference with usual social & functional activities	Pain causing greater than minimal interference with usual social & functional activities	Pain causing inability to perform usual social & functional activities	Disabling pain causing inability to perform basic self-care functions OR Hospitalization (other than emergency room visit) indicated

Basic Self-care Functions – Adult: Activities such as bathing, dressing, toileting, transfer/movement, continence, and feeding.

Basic Self-care Functions – Young Children: Activities that are age and culturally appropriate (e.g., feeding self with culturally appropriate eating implement).

Usual Social & Functional Activities – Adult: Adaptive tasks and desirable activities, such as going to work, shopping, cooking, use of transportation, pursuing a hobby, etc.

Usual Social & Functional Activities – Young Children: Activities that are age and culturally appropriate (e.g., social interactions, play activities, learning tasks, etc.).

DIVISION OF AIDS TABLE FOR GRADING THE SEVERITY OF ADULT AND PEDIATRIC ADVERSE EVENTS
PUBLISH DATE: DECEMBER, 2004
(With PTT and seizure grading criteria amended for VRC 012)

CLINICAL				
**PARAMETER**	**GRADE 1 MILD**	**GRADE 2 MODERATE**	**GRADE 3 SEVERE**	**GRADE 4 POTENTIALLY LIFE-THREATENING**
Unintentional weight loss	NA	5 – 9% loss in body weight from baseline	10 – 19% loss in body weight from baseline	≥ 20% loss in body weight from baseline OR Aggressive intervention indicated [e.g., tube feeding or total parenteral nutrition (TPN)]
**INFECTION**				
Infection (any other than HIV infection)	Localized, no systemic antimicrobial treatment indicated AND Symptoms causing no or minimal interference with usual social & functional activities	Systemic antimicrobial treatment indicated OR Symptoms causing greater than minimal interference with usual social & functional activities	Systemic antimicrobial treatment indicated AND Symptoms causing inability to perform usual social & functional activities OR Operative intervention (other than simple incision and drainage) indicated	Life-threatening consequences (e.g., septic shock)
**INJECTION SITE REACTIONS**				
Injection site pain (pain without touching)<br>Or<br>Tenderness (pain when area is touched)	Pain/tenderness causing no or minimal limitation of use of limb	Pain/tenderness limiting use of limb OR Pain/tenderness causing greater than minimal interference with usual social & functional activities	Pain/tenderness causing inability to perform usual social & functional activities	Pain/tenderness causing inability to perform basic self-care function OR Hospitalization (other than emergency room visit) indicated for management of pain/tenderness
Injection site reaction (localized)				
**Adult > 15 years**	Erythema OR Induration of 5x5 cm – 9x9 cm (or 25 cm^2 – 81cm^2)	Erythema OR Induration OR Edema > 9 cm any diameter (or > 81 cm^2)	Ulceration OR Secondary infection OR Phlebitis OR Sterile abscess OR Drainage	Necrosis (involving dermis and deeper tissue)
**Pediatric ≤ 15 years**	Erythema OR Induration OR Edema present but ≤ 2.5 cm diameter	Erythema OR Induration OR Edema > 2.5 cm diameter but < 50% surface area of the extremity segment (e.g., upper arm/thigh)	Erythema OR Induration OR Edema involving ≥ 50% surface area of the extremity segment (e.g., upper arm/thigh) OR Ulceration OR Secondary infection OR Phlebitis OR Sterile abscess OR Drainage	Necrosis (involving dermis and deeper tissue)

Basic Self-care Functions – Adult: Activities such as bathing, dressing, toileting, transfer/movement, continence, and feeding.

Basic Self-care Functions – Young Children: Activities that are age and culturally appropriate (e.g., feeding self with culturally appropriate eating implement).

Usual Social & Functional Activities – Adult: Adaptive tasks and desirable activities, such as going to work, shopping, cooking, use of transportation, pursuing a hobby, etc.

Usual Social & Functional Activities – Young Children: Activities that are age and culturally appropriate (e.g., social interactions, play activities, learning tasks, etc.).

DIVISION OF AIDS TABLE FOR GRADING THE SEVERITY OF ADULT AND PEDIATRIC ADVERSE EVENTS
PUBLISH DATE: DECEMBER, 2004
(With PTT and seizure grading criteria amended for VRC 012)

CLINICAL				
**PARAMETER**	**GRADE 1 MILD**	**GRADE 2 MODERATE**	**GRADE 3 SEVERE**	**GRADE 4 POTENTIALLY LIFE-THREATENING**
Pruritis associated with injection<br>See also Skin: Pruritis (itching - no skin lesions)	Itching localized to injection site AND Relieved spontaneously or with < 48 hours treatment	Itching beyond the injection site but not generalized OR Itching localized to injection site requiring ≥ 48 hours treatment	Generalized itching causing inability to perform usual social & functional activities	NA
**SKIN – DERMATOLOGICAL**				
Alopecia	Thinning detectable by study participant (or by caregiver for young children and disabled adults)	Thinning or patchy hair loss detectable by health care provider	Complete hair loss	NA
Cutaneous reaction – rash	Localized macular rash	Diffuse macular, maculopapular, or morbilliform rash OR Target lesions	Diffuse macular, maculopapular, or morbilliform rash with vesicles or limited number of bullae OR Superficial ulcerations of mucous membrane limited to one site	Extensive or generalized bullous lesions OR Stevens-Johnson syndrome OR Ulceration of mucous membrane involving two or more distinct mucosal sites OR Toxic epidermal necrolysis (TEN)
Hyperpigmentation	Slight or localized	Marked or generalized	NA	NA
Hypopigmentation	Slight or localized	Marked or generalized	NA	NA
Pruritis (itching – no skin lesions)<br>(See also Injection Site Reactions: Pruritis associated with injection)	Itching causing no or minimal interference with usual social & functional activities	Itching causing greater than minimal interference with usual social & functional activities	Itching causing inability to perform usual social & functional activities	NA
**CARDIOVASCULAR**				
Cardiac arrhythmia (general)<br>(By ECG or physical exam)	Asymptomatic AND No intervention indicated	Asymptomatic AND Non-urgent medical intervention indicated	Symptomatic, non-life-threatening AND Non-urgent medical intervention indicated	Life-threatening arrhythmia OR Urgent intervention indicated
Cardiac-ischemia/infarction	NA	NA	Symptomatic ischemia (stable angina) OR Testing consistent with ischemia	Unstable angina OR Acute myocardial infarction

Basic Self-care Functions – Adult: Activities such as bathing, dressing, toileting, transfer/movement, continence, and feeding.

Basic Self-care Functions – Young Children: Activities that are age and culturally appropriate (e.g., feeding self with culturally appropriate eating implement).

Usual Social & Functional Activities – Adult: Adaptive tasks and desirable activities, such as going to work, shopping, cooking, use of transportation, pursuing a hobby, etc.

Usual Social & Functional Activities – Young Children: Activities that are age and culturally appropriate (e.g., social interactions, play activities, learning tasks, etc.).

DIVISION OF AIDS TABLE FOR GRADING THE SEVERITY OF ADULT AND PEDIATRIC ADVERSE EVENTS
PUBLISH DATE: DECEMBER, 2004
(With PTT and seizure grading criteria amended for VRC 012)

CLINICAL					
**PARAMETER**		**GRADE 1 MILD**	**GRADE 2 MODERATE**	**GRADE 3 SEVERE**	**GRADE 4 POTENTIALLY LIFE-THREATENING**
Hemorrhage (significant acute blood loss)		NA	Symptomatic AND No transfusion indicated	Symptomatic AND Transfusion of ≤ 2 units packed RBCs (for children ≤ 10 cc/kg) indicated	Life-threatening hypotension OR Transfusion of > 2 units packed RBCs (for children > 10 cc/kg) indicated
Hypertension					
	**Adult > 17 years** (with repeat testing at same visit)	> 140 – 159 mmHg systolic OR > 90 – 99 mmHg diastolic	≥ 160 – 179 mmHg systolic OR ≥ 100 – 109 mmHg diastolic	≥ 180 mmHg systolic OR ≥ 110 mmHg diastolic	Life-threatening consequences (e.g., malignant hypertension) OR Hospitalization indicated (other than emergency room visit)
	**Pediatric ≤ 17 years** (with repeat testing at same visit)	NA	91st – 94th percentile adjusted for age, height, and gender (systolic and/or diastolic)	≥ 95th percentile adjusted for age, height, and gender (systolic and/or diastolic)	Life-threatening consequences (e.g., malignant hypertension) OR Hospitalization indicated (other than emergency room visit)
Hypotension		NA	Symptomatic, corrected with oral fluid replacement	Symptomatic, IV fluids indicated	Shock requiring use of vasopressors or mechanical assistance to maintain blood pressure
Pericardial effusion		Asymptomatic, small effusion requiring no intervention	Asymptomatic, moderate or larger effusion requiring no intervention	Effusion with non-life threatening physiologic consequences OR Effusion with non-urgent intervention indicated	Life-threatening consequences (e.g., tamponade) OR Urgent intervention indicated
Prolonged PR interval					
	**Adult > 16 years**	PR interval 0.21 – 0.25 sec	PR interval > 0.25 sec	Type II 2nd degree AV block OR Ventricular pause > 3.0 sec	Complete AV block
	**Pediatric ≤ 16 years**	1st degree AV block (PR > normal for age and rate)	Type I 2nd degree AV block	Type II 2nd degree AV block	Complete AV block

Basic Self-care Functions – Adult: Activities such as bathing, dressing, toileting, transfer/movement, continence, and feeding.

Basic Self-care Functions – Young Children: Activities that are age and culturally appropriate (e.g., feeding self with culturally appropriate eating implement).

Usual Social & Functional Activities – Adult: Adaptive tasks and desirable activities, such as going to work, shopping, cooking, use of transportation, pursuing a hobby, etc.

Usual Social & Functional Activities – Young Children: Activities that are age and culturally appropriate (e.g., social interactions, play activities, learning tasks, etc.).

CLINICAL				
**PARAMETER**	**GRADE 1 MILD**	**GRADE 2 MODERATE**	**GRADE 3 SEVERE**	**GRADE 4 POTENTIALLY LIFE-THREATENING**
Prolonged QTc				
**Adult > 16 years**	Asymptomatic, QTc interval 0.45 – 0.47 sec OR Increase interval < 0.03 sec above baseline	Asymptomatic, QTc interval 0.48 – 0.49 sec OR Increase in interval 0.03 – 0.05 sec above baseline	Asymptomatic, QTc interval ≥ 0.50 sec OR Increase in interval ≥ 0.06 sec above baseline	Life-threatening consequences, e.g. Torsade de pointes or other associated serious ventricular dysrhythmia
**Pediatric ≤ 16 years**	Asymptomatic, QTc interval 0.450 – 0.464 sec	Asymptomatic, QTc interval 0.465 – 0.479 sec	Asymptomatic, QTc interval ≥ 0.480 sec	Life-threatening consequences, e.g. Torsade de pointes or other associated serious ventricular dysrhythmia
Thrombosis/embolism	NA	Deep vein thrombosis AND No intervention indicated (e.g., anticoagulation, lysis filter, invasive procedure)	Deep vein thrombosis AND Intervention indicated (e.g., anticoagulation, lysis filter, invasive procedure)	Embolic event (e.g., pulmonary embolism, life-threatening thrombus)
Vasovagal episode (associated with a procedure of any kind)	Present without loss of consciousness	Present with transient loss of consciousness	NA	NA
Ventricular dysfunction (congestive heart failure)	NA	Asymptomatic diagnostic finding AND intervention indicated	New onset with symptoms OR Worsening symptomatic congestive heart failure	Life-threatening congestive heart failure
**GASTROINTESTINAL**				
Anorexia	Loss of appetite without decreased oral intake	Loss of appetite associated with decreased oral intake without significant weight loss	Loss of appetite associated with significant weight loss	Life-threatening consequences OR Aggressive intervention indicated [e.g., tube feeding or total parenteral nutrition (TPN)]
Ascites	Asymptomatic	Symptomatic AND Intervention indicated (e.g., diuretics or therapeutic paracentesis)	Symptomatic despite intervention	Life-threatening consequences

CLINICAL

PARAMETER	GRADE 1 MILD	GRADE 2 MODERATE	GRADE 3 SEVERE	GRADE 4 POTENTIALLY LIFE-THREATENING
Cholecystitis	NA	Symptomatic AND Medical intervention indicated	Radiologic, endoscopic, or operative intervention indicated	Life-threatening consequences (e.g., sepsis or perforation)
Constipation	NA	Persistent constipation requiring regular use of dietary modifications, laxatives, or enemas	Obstipation with manual evacuation indicated	Life-threatening consequences (e.g., obstruction)
Diarrhea				
**Adult and Pediatric ≥ 1 year**	Transient or intermittent episodes of unformed stools OR Increase of ≤ 3 stools over baseline per 24-hour period	Persistent episodes of unformed to watery stools OR Increase of 4 – 6 stools over baseline per 24-hour period	Bloody diarrhea OR Increase of ≥ 7 stools per 24-hour period OR IV fluid replacement indicated	Life-threatening consequences (e.g., hypotensive shock)
**Pediatric < 1 year**	Liquid stools (more unformed than usual) but usual number of stools	Liquid stools with increased number of stools OR Mild dehydration	Liquid stools with moderate dehydration	Liquid stools resulting in severe dehydration with aggressive rehydration indicated OR Hypotensive shock
Dysphagia-Odynophagia	Symptomatic but able to eat usual diet	Symptoms causing altered dietary intake without medical intervention indicated	Symptoms causing severely altered dietary intake with medical intervention indicated	Life-threatening reduction in oral intake
Mucositis/stomatitis (clinical exam)<br>Indicate site (e.g., larynx, oral)<br>See Genitourinary for Vulvovaginitis<br>See also Dysphagia-Odynophagia and Proctitis	Erythema of the mucosa	Patchy pseudomembranes or ulcerations	Confluent pseudomembranes or ulcerations OR Mucosal bleeding with minor trauma	Tissue necrosis OR Diffuse spontaneous mucosal bleeding OR Life-threatening consequences (e.g., aspiration, choking)
Nausea	Transient (< 24 hours) or intermittent nausea with no or minimal interference with oral intake	Persistent nausea resulting in decreased oral intake for 24 – 48 hours	Persistent nausea resulting in minimal oral intake for > 48 hours OR Aggressive rehydration indicated (e.g., IV fluids)	Life-threatening consequences (e.g., hypotensive shock)

Basic Self-care Functions – Adult: Activities such as bathing, dressing, toileting, transfer/movement, continence, and feeding.

Basic Self-care Functions – Young Children: Activities that are age and culturally appropriate (e.g., feeding self with culturally appropriate eating implement).

Usual Social & Functional Activities – Adult: Adaptive tasks and desirable activities, such as going to work, shopping, cooking, use of transportation, pursuing a hobby, etc.

Usual Social & Functional Activities – Young Children: Activities that are age and culturally appropriate (e.g., social interactions, play activities, learning tasks, etc.).

DIVISION OF AIDS TABLE FOR GRADING THE SEVERITY OF ADULT AND PEDIATRIC ADVERSE EVENTS
PUBLISH DATE: DECEMBER, 2004
(With PTT and seizure grading criteria amended for VRC 012)

CLINICAL				
**PARAMETER**	**GRADE 1 MILD**	**GRADE 2 MODERATE**	**GRADE 3 SEVERE**	**GRADE 4 POTENTIALLY LIFE-THREATENING**
Pancreatitis	NA	Symptomatic AND Hospitalization not indicated (other than emergency room visit)	Symptomatic AND Hospitalization indicated (other than emergency room visit)	Life-threatening consequences (e.g., circulatory failure, hemorrhage, sepsis)
Proctitis (functional-symptomatic)<br>Also see Mucositis/stomatitis for clinical exam	Rectal discomfort AND No intervention indicated	Symptoms causing greater than minimal interference with usual social & functional activities OR Medical intervention indicated	Symptoms causing inability to perform usual social & functional activities OR Operative intervention indicated	Life-threatening consequences (e.g., perforation)
Vomiting	Transient or intermittent vomiting with no or minimal interference with oral intake	Frequent episodes of vomiting with no or mild dehydration	Persistent vomiting resulting in orthostatic hypotension OR Aggressive rehydration indicated (e.g., IV fluids)	Life-threatening consequences (e.g., hypotensive shock)
**NEUROLOGIC**				
Alteration in personality-behavior or in mood (e.g., agitation, anxiety, depression, mania, psychosis)	Alteration causing no or minimal interference with usual social & functional activities	Alteration causing greater than minimal interference with usual social & functional activities	Alteration causing inability to perform usual social & functional activities	Behavior potentially harmful to self or others (e.g., suicidal and homicidal ideation or attempt, acute psychosis) OR Causing inability to perform basic self-care functions
Altered Mental Status<br>For Dementia, see Cognitive and behavioral/attentional disturbance (including dementia and attention deficit disorder)	Changes causing no or minimal interference with usual social & functional activities	Mild lethargy or somnolence causing greater than minimal interference with usual social & functional activities	Confusion, memory impairment, lethargy, or somnolence causing inability to perform usual social & functional activities	Delirium OR obtundation, OR coma
Ataxia	Asymptomatic ataxia detectable on exam OR Minimal ataxia causing no or minimal interference with usual social & functional activities	Symptomatic ataxia causing greater than minimal interference with usual social & functional activities	Symptomatic ataxia causing inability to perform usual social & functional activities	Disabling ataxia causing inability to perform basic self-care functions

Basic Self-care Functions – Adult: Activities such as bathing, dressing, toileting, transfer/movement, continence, and feeding.

Basic Self-care Functions – Young Children: Activities that are age and culturally appropriate (e.g., feeding self with culturally appropriate eating implement).

Usual Social & Functional Activities – Adult: Adaptive tasks and desirable activities, such as going to work, shopping, cooking, use of transportation, pursuing a hobby, etc.

Usual Social & Functional Activities – Young Children: Activities that are age and culturally appropriate (e.g., social interactions, play activities, learning tasks, etc.).

CLINICAL				
**PARAMETER**	**GRADE 1 MILD**	**GRADE 2 MODERATE**	**GRADE 3 SEVERE**	**GRADE 4 POTENTIALLY LIFE-THREATENING**
Cognitive and behavioral/attentional disturbance (including dementia and attention deficit disorder)	Disability causing no or minimal interference with usual social & functional activities OR Specialized resources not indicated	Disability causing greater than minimal interference with usual social & functional activities OR Specialized resources on part-time basis indicated	Disability causing inability to perform usual social & functional activities OR Specialized resources on a full-time basis indicated	Disability causing inability to perform basic self-care functions OR Institutionalization indicated
CNS ischemia (acute)	NA	NA	Transient ischemic attack	Cerebral vascular accident (CVA, stroke) with neurological deficit
Developmental delay – **Pediatric ≤ 16 years**	Mild developmental delay, either motor or cognitive, as determined by comparison with a developmental screening tool appropriate for the setting	Moderate developmental delay, either motor or cognitive, as determined by comparison with a developmental screening tool appropriate for the setting	Severe developmental delay, either motor or cognitive, as determined by comparison with a developmental screening tool appropriate for the setting	Developmental regression, either motor or cognitive, as determined by comparison with a developmental screening tool appropriate for the setting
Headache	Symptoms causing no or minimal interference with usual social & functional activities	Symptoms causing greater than minimal interference with usual social & functional activities	Symptoms causing inability to perform usual social & functional activities	Symptoms causing inability to perform basic self-care functions OR Hospitalization indicated (other than emergency room visit) OR Headache with significant impairment of alertness or other neurologic function
Insomnia	NA	Difficulty sleeping causing greater than minimal interference with usual social & functional activities	Difficulty sleeping causing inability to perform usual social & functional activities	Disabling insomnia causing inability to perform basic self-care functions

Basic Self-care Functions – Adult: Activities such as bathing, dressing, toileting, transfer/movement, continence, and feeding.

Basic Self-care Functions – Young Children: Activities that are age and culturally appropriate (e.g., feeding self with culturally appropriate eating implement).

Usual Social & Functional Activities – Adult: Adaptive tasks and desirable activities, such as going to work, shopping, cooking, use of transportation, pursuing a hobby, etc.

Usual Social & Functional Activities – Young Children: Activities that are age and culturally appropriate (e.g., social interactions, play activities, learning tasks, etc.).

**CLINICAL**				
**PARAMETER**	**GRADE 1**<br>**MILD**	**GRADE 2**<br>**MODERATE**	**GRADE 3**<br>**SEVERE**	**GRADE 4**<br>**POTENTIALLY LIFE-THREATENING**
Neuromuscular weakness (including myopathy & neuropathy)	Asymptomatic with decreased strength on exam OR Minimal muscle weakness causing no or minimal interference with usual social & functional activities	Muscle weakness causing greater than minimal interference with usual social & functional activities	Muscle weakness causing inability to perform usual social & functional activities	Disabling muscle weakness causing inability to perform basic self-care functions OR Respiratory muscle weakness impairing ventilation
Neurosensory alteration (including paresthesia and painful neuropathy)	Asymptomatic with sensory alteration on exam or minimal paresthesia causing no or minimal interference with usual social & functional activities	Sensory alteration or paresthesia causing greater than minimal interference with usual social & functional activities	Sensory alteration or paresthesia causing inability to perform usual social & functional activities	Disabling sensory alteration or paresthesia causing inability to perform basic self-care functions
Seizure: (new onset) **– Adult ≥ 18 years**<br>See also Seizure: (known pre-existing seizure disorder)	NA	NA	new onset seizure	Seizures of any kind which are prolonged, repetitive (e.g., status epilepticus), or difficult to control (e.g., refractory epilepsy)
Seizure: (known pre-existing seizure disorder) **– Adult ≥ 18 years**<br>For worsening of existing epilepsy the grades should be based on an increase from previous level of control to any of these levels.	NA	Increased frequency of pre-existing seizures (non-repetitive) without change in seizure character OR Infrequent break-through seizures while on stable medication in a previously controlled seizure disorder	Change in seizure character from baseline either in duration or quality (e.g., severity or focality)	Seizures of any kind which are prolonged, repetitive (e.g., status epilepticus), or difficult to control (e.g., refractory epilepsy)
Seizure **– Pediatric < 18 years**	Seizure, generalized onset with or without secondary generalization, lasting < 5 minutes with < 24 hours post ictal state	Seizure, generalized onset with or without secondary generalization, lasting 5 – 20 minutes with < 24 hours post ictal state	Seizure, generalized onset with or without secondary generalization, lasting > 20 minutes	Seizure, generalized onset with or without secondary generalization, requiring intubation and sedation
Syncope (not associated with a procedure)	NA	Present	NA	NA

Basic Self-care Functions – Adult: Activities such as bathing, dressing, toileting, transfer/movement, continence, and feeding.

Basic Self-care Functions – Young Children: Activities that are age and culturally appropriate (e.g., feeding self with culturally appropriate eating implement).

Usual Social & Functional Activities – Adult: Adaptive tasks and desirable activities, such as going to work, shopping, cooking, use of transportation, pursuing a hobby, etc.

Usual Social & Functional Activities – Young Children: Activities that are age and culturally appropriate (e.g., social interactions, play activities, learning tasks, etc.).

DIVISION OF AIDS TABLE FOR GRADING THE SEVERITY OF ADULT AND PEDIATRIC ADVERSE EVENTS PUBLISH DATE: DECEMBER, 2004

(With PTT and seizure grading criteria amended for VRC 012)

CLINICAL					
**PARAMETER**		**GRADE 1 MILD**	**GRADE 2 MODERATE**	**GRADE 3 SEVERE**	**GRADE 4 POTENTIALLY LIFE-THREATENING**
Vertigo		Vertigo causing no or minimal interference with usual social & functional activities	Vertigo causing greater than minimal interference with usual social & functional activities	Vertigo causing inability to perform usual social & functional activities	Disabling vertigo causing inability to perform basic self-care functions
**RESPIRATORY**					
Bronchospasm (acute)		FEV1 or peak flow reduced to 70 – 80%	FEV1 or peak flow 50 – 69%	FEV1 or peak flow 25 – 49%	Cyanosis OR FEV1 or peak flow < 25% OR Intubation
Dyspnea or respiratory distress					
	**Adult ≥ 14 years**	Dyspnea on exertion with no or minimal interference with usual social & functional activities	Dyspnea on exertion causing greater than minimal interference with usual social & functional activities	Dyspnea at rest causing inability to perform usual social & functional activities	Respiratory failure with ventilatory support indicated
	**Pediatric < 14 years**	Wheezing OR minimal increase in respiratory rate for age	Nasal flaring OR Intercostal retractions OR Pulse oximetry 90 – 95%	Dyspnea at rest causing inability to perform usual social & functional activities OR Pulse oximetry < 90%	Respiratory failure with ventilatory support indicated
**MUSCULOSKELETAL**					
Arthralgia<br>See also Arthritis		Joint pain causing no or minimal interference with usual social & functional activities	Joint pain causing greater than minimal interference with usual social & functional activities	Joint pain causing inability to perform usual social & functional activities	Disabling joint pain causing inability to perform basic self-care functions
Arthritis<br>See also Arthralgia		Stiffness or joint swelling causing no or minimal interference with usual social & functional activities	Stiffness or joint swelling causing greater than minimal interference with usual social & functional activities	Stiffness or joint swelling causing inability to perform usual social & functional activities	Disabling joint stiffness or swelling causing inability to perform basic self-care functions
Bone Mineral Loss					
	**Adult ≥ 21 years**	BMD t-score -2.5 to -1.0	BMD t-score < -2.5	Pathological fracture (including loss of vertebral height)	Pathologic fracture causing life-threatening consequences
	**Pediatric < 21 years**	BMD z-score -2.5 to -1.0	BMD z-score < -2.5	Pathological fracture (including loss of vertebral height)	Pathologic fracture causing life-threatening consequences

Basic Self-care Functions – Adult: Activities such as bathing, dressing, toileting, transfer/movement, continence, and feeding.

Basic Self-care Functions – Young Children: Activities that are age and culturally appropriate (e.g., feeding self with culturally appropriate eating implement).

Usual Social & Functional Activities – Adult: Adaptive tasks and desirable activities, such as going to work, shopping, cooking, use of transportation, pursuing a hobby, etc.

Usual Social & Functional Activities – Young Children: Activities that are age and culturally appropriate (e.g., social interactions, play activities, learning tasks, etc.).

CLINICAL				
**PARAMETER**	**GRADE 1<br>MILD**	**GRADE 2<br>MODERATE**	**GRADE 3<br>SEVERE**	**GRADE 4<br>POTENTIALLY LIFE-THREATENING**
Myalgia (non-injection site)	Muscle pain causing no or minimal interference with usual social & functional activities	Muscle pain causing greater than minimal interference with usual social & functional activities	Muscle pain causing inability to perform usual social & functional activities	Disabling muscle pain causing inability to perform basic self-care functions
Osteonecrosis	NA	Asymptomatic with radiographic findings AND No operative intervention indicated	Symptomatic bone pain with radiographic findings OR Operative intervention indicated	Disabling bone pain with radiographic findings causing inability to perform basic self-care functions
**GENITOURINARY**				
Cervicitis (symptoms)<br>(For use in studies evaluating topical study agents)<br>For other cervicitis see Infection: Infection (any other than HIV infection)	Symptoms causing no or minimal interference with usual social & functional activities	Symptoms causing greater than minimal interference with usual social & functional activities	Symptoms causing inability to perform usual social & functional activities	Symptoms causing inability to perform basic self-care functions
Cervicitis (clinical exam)<br>(For use in studies evaluating topical study agents)<br>For other cervicitis see Infection: Infection (any other than HIV infection)	Minimal cervical abnormalities on examination (erythema, mucopurulent discharge, or friability) OR Epithelial disruption < 25% of total surface	Moderate cervical abnormalities on examination (erythema, mucopurulent discharge, or friability) OR Epithelial disruption of 25 – 49% total surface	Severe cervical abnormalities on examination (erythema, mucopurulent discharge, or friability) OR Epithelial disruption 50 – 75% total surface	Epithelial disruption > 75% total surface
Inter-menstrual bleeding (IMB)	Spotting observed by participant OR Minimal blood observed during clinical or colposcopic examination	Inter-menstrual bleeding not greater in duration or amount than usual menstrual cycle	Inter-menstrual bleeding greater in duration or amount than usual menstrual cycle	Hemorrhage with life-threatening hypotension OR Operative intervention indicated
Urinary tract obstruction (e.g., stone)	NA	Signs or symptoms of urinary tract obstruction without hydronephrosis or renal dysfunction	Signs or symptoms of urinary tract obstruction with hydronephrosis or renal dysfunction	Obstruction causing life-threatening consequences

Basic Self-care Functions – Adult: Activities such as bathing, dressing, toileting, transfer/movement, continence, and feeding.

Basic Self-care Functions – Young Children: Activities that are age and culturally appropriate (e.g., feeding self with culturally appropriate eating implement).

Usual Social & Functional Activities – Adult: Adaptive tasks and desirable activities, such as going to work, shopping, cooking, use of transportation, pursuing a hobby, etc.

Usual Social & Functional Activities – Young Children: Activities that are age and culturally appropriate (e.g., social interactions, play activities, learning tasks, etc.).

CLINICAL				
**PARAMETER**	**GRADE 1 MILD**	**GRADE 2 MODERATE**	**GRADE 3 SEVERE**	**GRADE 4 POTENTIALLY LIFE-THREATENING**
Vulvovaginitis (symptoms) (Use in studies evaluating topical study agents) For other vulvovaginitis see Infection: Infection (any other than HIV infection)	Symptoms causing no or minimal interference with usual social & functional activities	Symptoms causing greater than minimal interference with usual social & functional activities	Symptoms causing inability to perform usual social & functional activities	Symptoms causing inability to perform basic self-care functions
Vulvovaginitis (clinical exam) (Use in studies evaluating topical study agents) For other vulvovaginitis see Infection: Infection (any other than HIV infection)	Minimal vaginal abnormalities on examination OR Epithelial disruption < 25% of total surface	Moderate vaginal abnormalities on examination OR Epithelial disruption of 25 - 49% total surface	Severe vaginal abnormalities on examination OR Epithelial disruption 50 - 75% total surface	Vaginal perforation OR Epithelial disruption > 75% total surface
**OCULAR/VISUAL**				
Uveitis	Asymptomatic but detectable on exam	Symptomatic anterior uveitis OR Medical intervention indicated	Posterior or pan-uveitis OR Operative intervention indicated	Disabling visual loss in affected eye(s)
Visual changes (from baseline)	Visual changes causing no or minimal interference with usual social & functional activities	Visual changes causing greater than minimal interference with usual social & functional activities	Visual changes causing inability to perform usual social & functional activities	Disabling visual loss in affected eye(s)
**ENDOCRINE/METABOLIC**				
Abnormal fat accumulation (e.g., back of neck, breasts, abdomen)	Detectable by study participant (or by caregiver for young children and disabled adults)	Detectable on physical exam by health care provider	Disfiguring OR Obvious changes on casual visual inspection	NA

Basic Self-care Functions – Adult: Activities such as bathing, dressing, toileting, transfer/movement, continence, and feeding.

Basic Self-care Functions – Young Children: Activities that are age and culturally appropriate (e.g., feeding self with culturally appropriate eating implement).

Usual Social & Functional Activities – Adult: Adaptive tasks and desirable activities, such as going to work, shopping, cooking, use of transportation, pursuing a hobby, etc.

Usual Social & Functional Activities – Young Children: Activities that are age and culturally appropriate (e.g., social interactions, play activities, learning tasks, etc.).

CLINICAL				
**PARAMETER**	**GRADE 1 MILD**	**GRADE 2 MODERATE**	**GRADE 3 SEVERE**	**GRADE 4 POTENTIALLY LIFE-THREATENING**
Diabetes mellitus	NA	New onset without need to initiate medication OR Modification of current medications to regain glucose control	New onset with initiation of medication indicated OR Diabetes uncontrolled despite treatment modification	Life-threatening consequences (e.g., ketoacidosis, hyperosmolar non-ketotic coma)
Gynecomastia	Detectable by study participant or caregiver (for young children and disabled adults)	Detectable on physical exam by health care provider	Disfiguring OR Obvious on casual visual inspection	NA
Hyperthyroidism	Asymptomatic	Symptomatic causing greater than minimal interference with usual social & functional activities OR Thyroid suppression therapy indicated	Symptoms causing inability to perform usual social & functional activities OR Uncontrolled despite treatment modification	Life-threatening consequences (e.g., thyroid storm)
Hypothyroidism	Asymptomatic	Symptomatic causing greater than minimal interference with usual social & functional activities OR Thyroid replacement therapy indicated	Symptoms causing inability to perform usual social & functional activities OR Uncontrolled despite treatment modification	Life-threatening consequences (e.g., myxedema coma)
Lipoatrophy (e.g., fat loss from the face, extremities, buttocks)	Detectable by study participant (or by caregiver for young children and disabled adults)	Detectable on physical exam by health care provider	Disfiguring OR Obvious on casual visual inspection	NA

Basic Self-care Functions – Adult: Activities such as bathing, dressing, toileting, transfer/movement, continence, and feeding.

Basic Self-care Functions – Young Children: Activities that are age and culturally appropriate (e.g., feeding self with culturally appropriate eating implement).

Usual Social & Functional Activities – Adult: Adaptive tasks and desirable activities, such as going to work, shopping, cooking, use of transportation, pursuing a hobby, etc.

Usual Social & Functional Activities – Young Children: Activities that are age and culturally appropriate (e.g., social interactions, play activities, learning tasks, etc.).

DIVISION OF AIDS TABLE FOR GRADING THE SEVERITY OF ADULT AND PEDIATRIC ADVERSE EVENTS
PUBLISH DATE: DECEMBER, 2004
(With PTT and seizure grading criteria amended for VRC 012)

LABORATORY				
**PARAMETER**	**GRADE 1 MILD**	**GRADE 2 MODERATE**	**GRADE 3 SEVERE**	**GRADE 4 POTENTIALLY LIFE-THREATENING**
**HEMATOLOGY**	***Standard International Units are listed in italics***			
Absolute CD4+ count **– Adult and Pediatric > 13 years** (HIV NEGATIVE ONLY)	300 – 400/mm^3 *300 – 400/µL*	200 – 299/mm^3 *200 – 299/µL*	100 – 199/mm^3 *100 – 199/µL*	< 100/mm^3 *< 100/µL*
Absolute lymphocyte count **– Adult and Pediatric > 13 years** (HIV NEGATIVE ONLY)	600 – 650/mm^3 *0.600 x 10^9 – 0.650 x 10^9/L*	500 – 599/mm^3 *0.500 x 10^9 – 0.599 x 10^9/L*	350 – 499/mm^3 *0.350 x 10^9 – 0.499 x 10^9/L*	< 350/mm^3 *< 0.350 x 10^9/L*
Absolute neutrophil count (ANC)				
**Adult and Pediatric, > 7 days**	1,000 – 1,300/mm^3 *1.000 x 10^9 – 1.300 x 10^9/L*	750 – 999/mm^3 *0.750 x 10^9 – 0.999 x 10^9/L*	500 – 749/mm^3 *0.500 x 10^9 – 0.749 x 10^9/L*	< 500/mm^3 *< 0.500 x 10^9/L*
**Infant**[*†]**, 2 – ≤ 7 days**	1,250 – 1,500/mm^3 *1.250 x 10^9 – 1.500 x 10^9/L*	1,000 – 1,249/mm^3 *1.000 x 10^9 – 1.249 x 10^9/L*	750 – 999/mm^3 *0.750 x 10^9 – 0.999 x 10^9/L*	< 750/mm^3 *< 0.750 x 10^9/L*
**Infant**[*†]**, 1 day**	4,000 – 5,000/mm^3 *4.000 x 10^9 – 5.000 x 10^9/L*	3,000 – 3,999/mm^3 *3.000 x 10^9 – 3.999 x10^9/L*	1,500 – 2,999/mm^3 *1.500 x 10^9 – 2.999 x 10^9/L*	< 1,500/mm^3 *< 1.500 x 10^9/L*
Fibrinogen, decreased	100 – 200 mg/dL *1.00 – 2.00 g/L* OR 0.75 – 0.99 x LLN	75 – 99 mg/dL *0.75 – 0.99 g/L* OR 0.50 – 0.74 x LLN	50 – 74 mg/dL *0.50 – 0.74 g/L* OR 0.25 – 0.49 x LLN	< 50 mg/dL *< 0.50 g/L* OR < 0.25 x LLN OR Associated with gross bleeding
Hemoglobin (Hgb)				
**Adult and Pediatric ≥ 57 days** (HIV POSITIVE ONLY)	8.5 – 10.0 g/dL *1.32 – 1.55 mmol/L*	7.5 – 8.4 g/dL *1.16 – 1.31 mmol/L*	6.50 – 7.4 g/dL *1.01 – 1.15 mmol/L*	< 6.5 g/dL *< 1.01 mmol/L*

Basic Self-care Functions – Adult: Activities such as bathing, dressing, toileting, transfer/movement, continence, and feeding.

Basic Self-care Functions – Young Children: Activities that are age and culturally appropriate (e.g., feeding self with culturally appropriate eating implement).

Usual Social & Functional Activities – Adult: Adaptive tasks and desirable activities, such as going to work, shopping, cooking, use of transportation, pursuing a hobby, etc.

Usual Social & Functional Activities – Young Children: Activities that are age and culturally appropriate (e.g., social interactions, play activities, learning tasks, etc.).

LABORATORY				
**PARAMETER**	**GRADE 1 MILD**	**GRADE 2 MODERATE**	**GRADE 3 SEVERE**	**GRADE 4 POTENTIALLY LIFE-THREATENING**
**Adult and Pediatric ≥ 57 days** (HIV NEGATIVE ONLY)	10.0 – 10.9 g/dL<br>*1.55 – 1.69 mmol/L*<br>OR<br>Any decrease<br>2.5 – 3.4 g/dL<br>*0.39 – 0.53 mmol/L*	9.0 – 9.9 g/dL<br>*1.40 – 1.54 mmol/L*<br>OR<br>Any decrease<br>3.5 – 4.4 g/dL<br>*0.54 – 0.68 mmol/L*	7.0 – 8.9 g/dL<br>*1.09 – 1.39 mmol/L*<br>OR<br>Any decrease<br>≥ 4.5 g/dL<br>*≥ 0.69 mmol/L*	< 7.0 g/dL<br>*< 1.09 mmol/L*
**Infant*†, 36 – 56 days** (HIV POSITIVE OR NEGATIVE)	8.5 – 9.4 g/dL<br>*1.32 – 1.46 mmol/L*	7.0 – 8.4 g/dL<br>*1.09 – 1.31 mmol/L*	6.0 – 6.9 g/dL<br>*0.93 – 1.08 mmol/L*	< 6.00 g/dL<br>*< 0.93 mmol/L*
**Infant*†, 22 – 35 days** (HIV POSITIVE OR NEGATIVE)	9.5 – 10.5 g/dL<br>*1.47 – 1.63 mmol/L*	8.0 – 9.4 g/dL<br>*1.24 – 1.46 mmol/L*	7.0 – 7.9 g/dL<br>*1.09 – 1.23 mmol/L*	< 7.00 g/dL<br>*< 1.09 mmol/L*
**Infant*†, 1 – 21 days** (HIV POSITIVE OR NEGATIVE)	12.0 – 13.0 g/dL<br>*1.86 – 2.02 mmol/L*	10.0 – 11.9 g/dL<br>*1.55 – 1.85 mmol/L*	9.0 – 9.9 g/dL<br>*1.40 – 1.54 mmol/L*	< 9.0 g/dL<br>*< 1.40 mmol/L*
International Normalized Ratio of prothrombin time (INR)	1.1 – 1.5 x ULN	1.6 – 2.0 x ULN	2.1 – 3.0 x ULN	> 3.0 x ULN
Methemoglobin	5.0 – 10.0%	10.1 – 15.0%	15.1 – 20.0%	> 20.0%
Prothrombin Time (PT)	1.1 – 1.25 x ULN	1.26 – 1.50 x ULN	1.51 – 3.00 x ULN	> 3.00 x ULN
Partial Thromboplastin Time (PTT)	1.1 – 1.25 x ULN	1.26 – 1.50 x ULN	1.51 – 3.00 x ULN	> 3.00 x ULN
Platelets, decreased	100,000 – 124,999/mm^3<br>*100.000 x 10^9 – 124.999 x 10^9/L*	50,000 – 99,999/mm^3<br>*50.000 x 10^9 – 99.999 x 10^9/L*	25,000 – 49,999/mm^3<br>*25.000 x 10^9 – 49.999 x 10^9/L*	< 25,000/mm^3<br>*< 25.000 x 10^9/L*
WBC, decreased	2,000 – 2,500/mm^3<br>*2.000 x 10^9 – 2.500 x 10^9/L*	1,500 – 1,999/mm^3<br>*1.500 x 10^9 – 1.999 x 10^9/L*	1,000 – 1,499/mm^3<br>*1.000 x 10^9 – 1.499 x 10^9/L*	< 1,000/mm^3<br>*< 1.000 x 10^9/L*
**CHEMISTRIES**	***Standard International Units are listed in italics***			
Acidosis	NA	pH < normal, but ≥ 7.3	pH < 7.3 without life-threatening consequences	pH < 7.3 with life-threatening consequences
Albumin, serum, low	3.0 g/dL – < LLN<br>*30 g/L – < LLN*	2.0 – 2.9 g/dL<br>*20 – 29 g/L*	< 2.0 g/dL<br>*< 20 g/L*	NA
Alkaline Phosphatase	1.25 – 2.5 x ULN†	2.6 – 5.0 x ULN†	5.1 – 10.0 x ULN†	> 10.0 x ULN†

Basic Self-care Functions – Adult: Activities such as bathing, dressing, toileting, transfer/movement, continence, and feeding.

Basic Self-care Functions – Young Children: Activities that are age and culturally appropriate (e.g., feeding self with culturally appropriate eating implement).

Usual Social & Functional Activities – Adult: Adaptive tasks and desirable activities, such as going to work, shopping, cooking, use of transportation, pursuing a hobby, etc.

Usual Social & Functional Activities – Young Children: Activities that are age and culturally appropriate (e.g., social interactions, play activities, learning tasks, etc.).

LABORATORY				
**PARAMETER**	**GRADE 1 MILD**	**GRADE 2 MODERATE**	**GRADE 3 SEVERE**	**GRADE 4 POTENTIALLY LIFE-THREATENING**
Alkalosis	NA	pH > normal, but ≤ 7.5	pH > 7.5 without life-threatening consequences	pH > 7.5 with life-threatening consequences
ALT (SGPT)	1.25 – 2.5 x ULN	2.6 – 5.0 x ULN	5.1 – 10.0 x ULN	> 10.0 x ULN
AST (SGOT)	1.25 – 2.5 x ULN	2.6 – 5.0 x ULN	5.1 – 10.0 x ULN	> 10.0 x ULN
Bicarbonate, serum, low	16.0 mEq/L – < LLN<br>*16.0 mmol/L – < LLN*	11.0 – 15.9 mEq/L<br>*11.0 – 15.9 mmol/L*	8.0 – 10.9 mEq/L<br>*8.0 – 10.9 mmol/L*	< 8.0 mEq/L<br>*< 8.0 mmol/L*
Bilirubin (Total)				
**Adult and Pediatric > 14 days**	1.1 – 1.5 x ULN	1.6 – 2.5 x ULN	2.6 – 5.0 x ULN	> 5.0 x ULN
**Infant*†, ≤ 14 days** (non-hemolytic)	NA	20.0 – 25.0 mg/dL<br>*342 – 428 μmol/L*	25.1 – 30.0 mg/dL<br>*429 – 513 μmol/L*	> 30.0 mg/dL<br>*> 513.0 μmol/L*
**Infant*†, ≤ 14 days** (hemolytic)	NA	NA	20.0 – 25.0 mg/dL<br>*342 – 428 μmol/L*	> 25.0 mg/dL<br>*> 428 μmol/L*
Calcium, serum, high (corrected for albumin)				
**Adult and Pediatric ≥ 7 days**	10.6 – 11.5 mg/dL<br>*2.65 – 2.88 mmol/L*	11.6 – 12.5 mg/dL<br>*2.89 – 3.13 mmol/L*	12.6 – 13.5 mg/dL<br>*3.14 – 3.38 mmol/L*	> 13.5 mg/dL<br>*> 3.38 mmol/L*
**Infant*†, < 7 days**	11.5 – 12.4 mg/dL<br>*2.88 – 3.10 mmol/L*	12.5 – 12.9 mg/dL<br>*3.11 – 3.23 mmol/L*	13.0 – 13.5 mg/dL<br>*3.245 – 3.38 mmol/L*	> 13.5 mg/dL<br>*> 3.38 mmol/L*
Calcium, serum, low (corrected for albumin)				
**Adult and Pediatric ≥ 7 days**	7.8 – 8.4 mg/dL<br>*1.95 – 2.10 mmol/L*	7.0 – 7.7 mg/dL<br>*1.75 – 1.94 mmol/L*	6.1 – 6.9 mg/dL<br>*1.53 – 1.74 mmol/L*	< 6.1 mg/dL<br>*< 1.53 mmol/L*
**Infant*†, < 7 days**	6.5 – 7.5 mg/dL<br>*1.63 – 1.88 mmol/L*	6.0 – 6.4 mg/dL<br>*1.50 – 1.62 mmol/L*	5.50 – 5.90 mg/dL<br>*1.38 – 1.51 mmol/L*	< 5.50 mg/dL<br>*< 1.38 mmol/L*
Cardiac troponin I (cTnI)	NA	NA	NA	Levels consistent with myocardial infarction or unstable angina as defined by the manufacturer
Cardiac troponin T (cTnT)	NA	NA	NA	≥ 0.20 ng/mL<br>OR<br>Levels consistent with myocardial infarction or unstable angina as defined by the manufacturer

Basic Self-care Functions – Adult: Activities such as bathing, dressing, toileting, transfer/movement, continence, and feeding.

Basic Self-care Functions – Young Children: Activities that are age and culturally appropriate (e.g., feeding self with culturally appropriate eating implement).

Usual Social & Functional Activities – Adult: Adaptive tasks and desirable activities, such as going to work, shopping, cooking, use of transportation, pursuing a hobby, etc.

Usual Social & Functional Activities – Young Children: Activities that are age and culturally appropriate (e.g., social interactions, play activities, learning tasks, etc.).

DIVISION OF AIDS TABLE FOR GRADING THE SEVERITY OF ADULT AND PEDIATRIC ADVERSE EVENTS
PUBLISH DATE: DECEMBER, 2004
(With PTT and seizure grading criteria amended for VRC 012)

LABORATORY

PARAMETER		GRADE 1 MILD	GRADE 2 MODERATE	GRADE 3 SEVERE	GRADE 4 POTENTIALLY LIFE-THREATENING
Cholesterol (fasting)					
	**Adult ≥ 18 years**	200 – 239 mg/dL<br>*5.18 – 6.19 mmol/L*	240 – 300 mg/dL<br>*6.20 – 7.77 mmol/L*	> 300 mg/dL<br>*> 7.77 mmol/L*	NA
	**Pediatric < 18 years**	170 – 199 mg/dL<br>*4.40 – 5.15 mmol/L*	200 – 300 mg/dL<br>*5.16 – 7.77 mmol/L*	> 300 mg/dL<br>*> 7.77 mmol/L*	NA
Creatine Kinase		3.0 – 5.9 x ULN[†]	6.0 – 9.9 x ULN[†]	10.0 – 19.9 x ULN[†]	≥ 20.0 x ULN[†]
Creatinine		1.1 – 1.3 x ULN[†]	1.4 – 1.8 x ULN[†]	1.9 – 3.4 x ULN[†]	≥ 3.5 x ULN[†]
Glucose, serum, high					
	Nonfasting	116 – 160 mg/dL<br>*6.44 – 8.88 mmol/L*	161 – 250 mg/dL<br>*8.89 – 13.88 mmol/L*	251 – 500 mg/dL<br>*13.89 – 27.75 mmol/L*	> 500 mg/dL<br>*> 27.75 mmol/L*
	Fasting	110 – 125 mg/dL<br>*6.11 – 6.94 mmol/L*	126 – 250 mg/dL<br>*6.95 – 13.88 mmol/L*	251 – 500 mg/dL<br>*13.89 – 27.75 mmol/L*	> 500 mg/dL<br>*> 27.75 mmol/L*
Glucose, serum, low					
	**Adult and Pediatric ≥ 1 month**	55 – 64 mg/dL<br>*3.05 – 3.55 mmol/L*	40 – 54 mg/dL<br>*2.22 – 3.06 mmol/L*	30 – 39 mg/dL<br>*1.67 – 2.23 mmol/L*	< 30 mg/dL<br>*< 1.67 mmol/L*
	**Infant[*†], < 1 month**	50 – 54 mg/dL<br>*2.78 – 3.00 mmol/L*	40 – 49 mg/dL<br>*2.22 – 2.77 mmol/L*	30 – 39 mg/dL<br>*1.67 – 2.21 mmol/L*	< 30 mg/dL<br>*< 1.67 mmol/L*
Lactate		< 2.0 x ULN without acidosis	≥ 2.0 x ULN without acidosis	Increased lactate with pH < 7.3 without life-threatening consequences	Increased lactate with pH < 7.3 with life-threatening consequences
LDL cholesterol (fasting)					
	**Adult ≥ 18 years**	130 – 159 mg/dL<br>*3.37 – 4.12 mmol/L*	160 – 190 mg/dL<br>*4.13 – 4.90 mmol/L*	≥ 190 mg/dL<br>*≥ 4.91 mmol/L*	NA
	**Pediatric > 2 - < 18 years**	110 – 129 mg/dL<br>*2.85 – 3.34 mmol/L*	130 – 189 mg/dL<br>*3.35 – 4.90 mmol/L*	≥ 190 mg/dL<br>*≥ 4.91 mmol/L*	NA
Lipase		1.1 – 1.5 x ULN	1.6 – 3.0 x ULN	3.1 – 5.0 x ULN	> 5.0 x ULN
Magnesium, serum, low		1.2 – 1.4 mEq/L<br>*0.60 – 0.70 mmol/L*	0.9 – 1.1 mEq/L<br>*0.45 – 0.59 mmol/L*	0.6 – 0.8 mEq/L<br>*0.30 – 0.44 mmol/L*	< 0.60 mEq/L<br>*< 0.30 mmol/L*
Pancreatic amylase		1.1 – 1.5 x ULN	1.6 – 2.0 x ULN	2.1 – 5.0 x ULN	> 5.0 x ULN
Phosphate, serum, low					

Basic Self-care Functions – Adult: Activities such as bathing, dressing, toileting, transfer/movement, continence, and feeding.

Basic Self-care Functions – Young Children: Activities that are age and culturally appropriate (e.g., feeding self with culturally appropriate eating implement).

Usual Social & Functional Activities – Adult: Adaptive tasks and desirable activities, such as going to work, shopping, cooking, use of transportation, pursuing a hobby, etc.

Usual Social & Functional Activities – Young Children: Activities that are age and culturally appropriate (e.g., social interactions, play activities, learning tasks, etc.).

LABORATORY				
**PARAMETER**	**GRADE 1 MILD**	**GRADE 2 MODERATE**	**GRADE 3 SEVERE**	**GRADE 4 POTENTIALLY LIFE-THREATENING**
**Adult and Pediatric > 14 years**	2.5 mg/dL – < LLN<br>*0.81 mmol/L – < LLN*	2.0 – 2.4 mg/dL<br>*0.65 – 0.80 mmol/L*	1.0 – 1.9 mg/dL<br>*0.32 – 0.64 mmol/L*	< 1.00 mg/dL<br>*< 0.32 mmol/L*
**Pediatric 1 year – 14 years**	3.0 – 3.5 mg/dL<br>*0.97 – 1.13 mmol/L*	2.5 – 2.9 mg/dL<br>*0.81 – 0.96 mmol/L*	1.5 – 2.4 mg/dL<br>*0.48 – 0.80 mmol/L*	< 1.50 mg/dL<br>*< 0.48 mmol/L*
**Pediatric < 1 year**	3.5 – 4.5 mg/dL<br>*1.13 – 1.45 mmol/L*	2.5 – 3.4 mg/dL<br>*0.81 – 1.12 mmol/L*	1.5 – 2.4 mg/dL<br>*0.48 – 0.80 mmol/L*	< 1.50 mg/dL<br>*< 0.48 mmol/L*
Potassium, serum, high	5.6 – 6.0 mEq/L<br>*5.6 – 6.0 mmol/L*	6.1 – 6.5 mEq/L<br>*6.1 – 6.5 mmol/L*	6.6 – 7.0 mEq/L<br>*6.6 – 7.0 mmol/L*	> 7.0 mEq/L<br>*> 7.0 mmol/L*
Potassium, serum, low	3.0 – 3.4 mEq/L<br>*3.0 – 3.4 mmol/L*	2.5 – 2.9 mEq/L<br>*2.5 – 2.9 mmol/L*	2.0 – 2.4 mEq/L<br>*2.0 – 2.4 mmol/L*	< 2.0 mEq/L<br>*< 2.0 mmol/L*
Sodium, serum, high	146 – 150 mEq/L<br>*146 – 150 mmol/L*	151 – 154 mEq/L<br>*151 – 154 mmol/L*	155 – 159 mEq/L<br>*155 – 159 mmol/L*	≥ 160 mEq/L<br>*≥ 160 mmol/L*
Sodium, serum, low	130 – 135 mEq/L<br>*130 – 135 mmol/L*	125 – 129 mEq/L<br>*125 – 129 mmol/L*	121 – 124 mEq/L<br>*121 – 124 mmol/L*	≤ 120 mEq/L<br>*≤ 120 mmol/L*
Triglycerides (fasting)	NA	500 – 750 mg/dL<br>*5.65 – 8.48 mmol/L*	751 – 1,200 mg/dL<br>*8.49 – 13.56 mmol/L*	> 1,200 mg/dL<br>*> 13.56 mmol/L*
Uric acid	7.5 – 10.0 mg/dL<br>*0.45 – 0.59 mmol/L*	10.1 – 12.0 mg/dL<br>*0.60 – 0.71 mmol/L*	12.1 – 15.0 mg/dL<br>*0.72 – 0.89 mmol/L*	> 15.0 mg/dL<br>*> 0.89 mmol/L*
**URINALYSIS**	***Standard International Units are listed in italics***			
Hematuria (microscopic)	6 – 10 RBC/HPF	> 10 RBC/HPF	Gross, with or without clots OR with RBC casts	Transfusion indicated
Proteinuria, random collection	1 +	2 – 3 +	4 +	NA
Proteinuria, 24 hour collection				
**Adult and Pediatric ≥ 10 years**	200 – 999 mg/24 h<br>*0.200 – 0.999 g/d*	1,000 – 1,999 mg/24 h<br>*1.000 – 1.999 g/d*	2,000 – 3,500 mg/24 h<br>*2.000 – 3.500 g/d*	> 3,500 mg/24 h<br>*> 3.500 g/d*
**Pediatric > 3 mo - < 10 years**	201 – 499 mg/m^2/24 h<br>*0.201 – 0.499 g/d*	500 – 799 mg/m^2/24 h<br>*0.500 – 0.799 g/d*	800 – 1,000 mg/m^2/24 h<br>*0.800 – 1.000 g/d*	> 1,000 mg/ m^2/24 h<br>*> 1.000 g/d*

Basic Self-care Functions – Adult: Activities such as bathing, dressing, toileting, transfer/movement, continence, and feeding.

Basic Self-care Functions – Young Children: Activities that are age and culturally appropriate (e.g., feeding self with culturally appropriate eating implement).

Usual Social & Functional Activities – Adult: Adaptive tasks and desirable activities, such as going to work, shopping, cooking, use of transportation, pursuing a hobby, etc.

Usual Social & Functional Activities – Young Children: Activities that are age and culturally appropriate (e.g., social interactions, play activities, learning tasks, etc.).
