# Supplementary material for: Safety and Immunogenicity of a rAd35-EnvA Prototype HIV-1 Vaccine in Combination with rAd5-EnvA in Healthy Adults (VRC 012)
Source: PLoS One. 2016 Nov 15;11(11):e0166393. doi: 10.1371/journal.pone.0166393 (PMC5112788; doi:10.1371/journal.pone.0166393)
Supplement: S1 Table — (PDF) [file pone.0166393.s006.pdf]

S1 Table Maximum Local Reactogenicity Summary by Vaccination Type

	Day 0 rAd35-EnvA 10^9	*Day 0 rAd35-EnvA 10^{10}*	*Day 0 rAd35-EnvA 10^{11}*	*Day 0 rAd5-EnvA 10^{10}*	*Week 12 rAd35-EnvA 10^{10}*	*Week 12 rAd35-EnvA 10^{11}*	*Week 12 rAd5-EnvA 10^{10}*	*All Subjects*
Symptoms Intensity	*(N=5)*	*(N=10)*	*(N=10)*	*(N=10)*	*(N=3)*	*(N=5)*	*(N=10)*	*(N=35)*
PAIN/TENDERNESS								
None	4 (80.0%)	6 (60.0%)	1 (10.0%)	2 (20.0%)	0 (0.0%)	1 (20.0%)	2 (20.0%)	10 (28.6%)
Mild	1 (20.0%)	4 (40.0%)	9 (90.0%)	8 (80.0%)	3 (100.0%)	4 (80.0%)	7 (70.0%)	24 (68.6%)
Moderate	0 (0.0%)	0 (0.0%)	0 (0.0%)	0 (0.0%)	0 (0.0%)	0 (0.0%)	1 (10.0%)	1 (2.9%)
Severe	0 (0.0%)	0 (0.0%)	0 (0.0%)	0 (0.0%)	0 (0.0%)	0 (0.0%)	0 (0.0%)	0 (0.0%)
Missing	0 (0.0%)	0 (0.0%)	0 (0.0%)	0 (0.0%)	0 (0.0%)	0 (0.0%)	0 (0.0%)	0 (0.0%)
SWELLING								
None	5 (100.0%)	9 (90.0%)	10 (100.0%)	9 (90.0%)	3 (100.0%)	3 (60.0%)	8 (80.0%)	31 (88.6%)
Mild	0 (0.0%)	1 (10.0%)	0 (0.0%)	1 (10.0%)	0 (0.0%)	2 (40.0%)	2 (20.0%)	4 (11.4%)
Moderate	0 (0.0%)	0 (0.0%)	0 (0.0%)	0 (0.0%)	0 (0.0%)	0 (0.0%)	0 (0.0%)	0 (0.0%)
Severe	0 (0.0%)	0 (0.0%)	0 (0.0%)	0 (0.0%)	0 (0.0%)	0 (0.0%)	0 (0.0%)	0 (0.0%)
Missing	0 (0.0%)	0 (0.0%)	0 (0.0%)	0 (0.0%)	0 (0.0%)	0 (0.0%)	0 (0.0%)	0 (0.0%)
REDNESS								
None	5 (100.0%)	9 (90.0%)	10 (100.0%)	9 (90.0%)	3 (100.0%)	4 (80.0%)	10 (100.0%)	33 (94.3%)
Mild	0 (0.0%)	1 (10.0%)	0 (0.0%)	1 (10.0%)	0 (0.0%)	1 (20.0%)	0 (0.0%)	2 (5.7%)
Moderate	0 (0.0%)	0 (0.0%)	0 (0.0%)	0 (0.0%)	0 (0.0%)	0 (0.0%)	0 (0.0%)	0 (0.0%)
Severe	0 (0.0%)	0 (0.0%)	0 (0.0%)	0 (0.0%)	0 (0.0%)	0 (0.0%)	0 (0.0%)	0 (0.0%)
Missing	0 (0.0%)	0 (0.0%)	0 (0.0%)	0 (0.0%)	0 (0.0%)	0 (0.0%)	0 (0.0%)	0 (0.0%)
ANY LOCAL SYMPTOM								
None	4 (80.0%)	6 (60.0%)	1 (10.0%)	2 (20.0%)	0 (0.0%)	1 (20.0%)	2 (20.0%)	10 (28.6%)
Mild	1 (20.0%)	4 (40.0%)	9 (90.0%)	8 (80.0%)	3 (100.0%)	4 (80.0%)	7 (70.0%)	24 (68.6%)
Moderate	0 (0.0%)	0 (0.0%)	0 (0.0%)	0 (0.0%)	0 (0.0%)	0 (0.0%)	1 (10.0%)	1 (2.9%)
Severe	0 (0.0%)	0 (0.0%)	0 (0.0%)	0 (0.0%)	0 (0.0%)	0 (0.0%)	0 (0.0%)	0 (0.0%)
Missing	0 (0.0%)	0 (0.0%)	0 (0.0%)	0 (0.0%)	0 (0.0%)	0 (0.0%)	0 (0.0%)	0 (0.0%)

MedDRA V14.0 Data as of August 5, 2011
