# Supplementary material for: Safety and Immunogenicity of a rAd35-EnvA Prototype HIV-1 Vaccine in Combination with rAd5-EnvA in Healthy Adults (VRC 012)
Source: PLoS One. 2016 Nov 15;11(11):e0166393. doi: 10.1371/journal.pone.0166393 (PMC5112788; doi:10.1371/journal.pone.0166393)
Supplement: S2 Table — (PDF) [file pone.0166393.s007.pdf]

S2 Table Maximum Subject Self-assessed Systemic Reactogenicity (i.e., Solicited Adverse Events) Summary by Vaccination Type

	Day 0 rAd35-EnvA 10^9	Day 0 rAd35-EnvA 10^{10}	Day 0 rAd35-EnvA 10^{11}	Day 0 rAd5-EnvA 10^{10}	Week 12 rAd35-EnvA 10^{10}	Week 12 rAd35-EnvA 10^{11}	Week 12 rAd5-EnvA 10^{10}	All Subjects
Symptoms Intensity	(N=5)	(N=10)	(N=10)	(N=10)	(N=3)	(N=5)	(N=10)	(N=35)
MALAISE								
None	4 (80.0%)	8 (80.0%)	6 (60.0%)	7 (70.0%)	1 (33.3%)	3 (60.0%)	5 (50.0%)	20 (57.1%)
Mild	1 (20.0%)	2 (20.0%)	4 (40.0%)	3 (30.0%)	2 (66.7%)	2 (40.0%)	2 (20.0%)	12 (34.3%)
Moderate	0 (0.0%)	0 (0.0%)	0 (0.0%)	0 (0.0%)	0 (0.0%)	0 (0.0%)	3 (30.0%)	3 (8.6%)
Severe	0 (0.0%)	0 (0.0%)	0 (0.0%)	0 (0.0%)	0 (0.0%)	0 (0.0%)	0 (0.0%)	0 (0.0%)
Missing	0 (0.0%)	0 (0.0%)	0 (0.0%)	0 (0.0%)	0 (0.0%)	0 (0.0%)	0 (0.0%)	0 (0.0%)
MYALGIA								
None	5 (100.0%)	7 (70.0%)	6 (60.0%)	9 (90.0%)	2 (66.7%)	3 (60.0%)	6 (60.0%)	24 (68.6%)
Mild	0 (0.0%)	3 (30.0%)	4 (40.0%)	1 (10.0%)	1 (33.3%)	2 (40.0%)	1 (10.0%)	8 (22.9%)
Moderate	0 (0.0%)	0 (0.0%)	0 (0.0%)	0 (0.0%)	0 (0.0%)	0 (0.0%)	3 (30.0%)	3 (8.6%)
Severe	0 (0.0%)	0 (0.0%)	0 (0.0%)	0 (0.0%)	0 (0.0%)	0 (0.0%)	0 (0.0%)	0 (0.0%)
Missing	0 (0.0%)	0 (0.0%)	0 (0.0%)	0 (0.0%)	0 (0.0%)	0 (0.0%)	0 (0.0%)	0 (0.0%)
HEADACHE								
None	3 (60.0%)	8 (80.0%)	6 (60.0%)	8 (80.0%)	3 (100.0%)	4 (80.0%)	6 (60.0%)	23 (65.7%)
Mild	2 (40.0%)	2 (20.0%)	4 (40.0%)	2 (20.0%)	0 (0.0%)	1 (20.0%)	2 (20.0%)	10 (28.6%)
Moderate	0 (0.0%)	0 (0.0%)	0 (0.0%)	0 (0.0%)	0 (0.0%)	0 (0.0%)	2 (20.0%)	2 (5.7%)
Severe	0 (0.0%)	0 (0.0%)	0 (0.0%)	0 (0.0%)	0 (0.0%)	0 (0.0%)	0 (0.0%)	0 (0.0%)
Missing	0 (0.0%)	0 (0.0%)	0 (0.0%)	0 (0.0%)	0 (0.0%)	0 (0.0%)	0 (0.0%)	0 (0.0%)
CHILLS								
None	5 (100.0%)	10 (100.0%)	9 (90.0%)	9 (90.0%)	3 (100.0%)	5 (100.0%)	6 (60.0%)	29 (82.9%)
Mild	0 (0.0%)	0 (0.0%)	1 (10.0%)	1 (10.0%)	0 (0.0%)	0 (0.0%)	3 (30.0%)	5 (14.3%)
Moderate	0 (0.0%)	0 (0.0%)	0 (0.0%)	0 (0.0%)	0 (0.0%)	0 (0.0%)	1 (10.0%)	1 (2.9%)
Severe	0 (0.0%)	0 (0.0%)	0 (0.0%)	0 (0.0%)	0 (0.0%)	0 (0.0%)	0 (0.0%)	0 (0.0%)
Missing	0 (0.0%)	0 (0.0%)	0 (0.0%)	0 (0.0%)	0 (0.0%)	0 (0.0%)	0 (0.0%)	0 (0.0%)

Symptoms Intensity	Day 0 rAd35-EnvA 10^9 (N=5)	Day 0 rAd35-EnvA 10^{10} (N=10)	Day 0 rAd35-EnvA 10^{11} (N=10)	Day 0 rAd5-EnvA 10^{10} (N=10)	Week 12 rAd35-EnvA 10^{10} (N=3)	Week 12 rAd35-EnvA 10^{11} (N=5)	Week 12 rAd5-EnvA 10^{10} (N=10)	All Subjects (N=35)
NAUSEA								
None	5 (100.0%)	9 (90.0%)	9 (90.0%)	10 (100.0%)	3 (100.0%)	5 (100.0%)	7 (70.0%)	32 (91.4%)
Mild	0 (0.0%)	1 (10.0%)	1 (10.0%)	0 (0.0%)	0 (0.0%)	0 (0.0%)	3 (30.0%)	3 (8.6%)
Moderate	0 (0.0%)	0 (0.0%)	0 (0.0%)	0 (0.0%)	0 (0.0%)	0 (0.0%)	0 (0.0%)	0 (0.0%)
Severe	0 (0.0%)	0 (0.0%)	0 (0.0%)	0 (0.0%)	0 (0.0%)	0 (0.0%)	0 (0.0%)	0 (0.0%)
Missing	0 (0.0%)	0 (0.0%)	0 (0.0%)	0 (0.0%)	0 (0.0%)	0 (0.0%)	0 (0.0%)	0 (0.0%)
TEMPERATURE								
None	5 (100.0%)	10 (100.0%)	9 (90.0%)	10 (100.0%)	3 (100.0%)	5 (100.0%)	9 (90.0%)	33 (94.3%)
Mild	0 (0.0%)	0 (0.0%)	1 (10.0%)	0 (0.0%)	0 (0.0%)	0 (0.0%)	1 (10.0%)	2 (5.7%)
Moderate	0 (0.0%)	0 (0.0%)	0 (0.0%)	0 (0.0%)	0 (0.0%)	0 (0.0%)	0 (0.0%)	0 (0.0%)
Severe	0 (0.0%)	0 (0.0%)	0 (0.0%)	0 (0.0%)	0 (0.0%)	0 (0.0%)	0 (0.0%)	0 (0.0%)
Missing	0 (0.0%)	0 (0.0%)	0 (0.0%)	0 (0.0%)	0 (0.0%)	0 (0.0%)	0 (0.0%)	0 (0.0%)
ANY SYSTEMIC SYMPTOM								
None	3 (60.0%)	7 (70.0%)	5 (50.0%)	7 (70.0%)	1 (33.3%)	3 (60.0%)	5 (50.0%)	17 (48.6%)
Mild	2 (40.0%)	3 (30.0%)	5 (50.0%)	3 (30.0%)	2 (66.7%)	2 (40.0%)	2 (20.0%)	15 (42.9%)
Moderate	0 (0.0%)	0 (0.0%)	0 (0.0%)	0 (0.0%)	0 (0.0%)	0 (0.0%)	3 (30.0%)	3 (8.6%)
Severe	0 (0.0%)	0 (0.0%)	0 (0.0%)	0 (0.0%)	0 (0.0%)	0 (0.0%)	0 (0.0%)	0 (0.0%)
Missing	0 (0.0%)	0 (0.0%)	0 (0.0%)	0 (0.0%)	0 (0.0%)	0 (0.0%)	0 (0.0%)	0 (0.0%)

MedDRA V14.0 Data as of August 5, 2011
